# Spatial Dynamics and Fine-Scale Vertical Behaviour of Immature Eastern Australasian White Sharks (*Carcharodon carcharias*)

**DOI:** 10.3390/biology11121689

**Published:** 2022-11-22

**Authors:** Julia L. Y. Spaet, Paul A. Butcher, Andrea Manica, Chi Hin Lam

**Affiliations:** 1 Evolutionary Ecology Group, Department of Zoology, University of Cambridge, Downing Street, Cambridge CB2 3EJ, UK; 2 Southern Cross University, Coffs Harbour, NSW 2450, Australia; 3 Fisheries NSW, NSW Department of Primary Industries, National Marine Science Centre, Coffs Harbour, NSW 2450, Australia; 4 Large Pelagics Research Center, Gloucester, MA 01931, USA

**Keywords:** archival tag, satellite telemetry, elasmobranch, diel behaviour, deep diving, migration, vertical migration, migration, Pacific Ocean, Australia

## Abstract

**Simple Summary:**

To understand how ecosystems function and to be able to better protect large marine predators, like sharks, it is important to understand how these animals move and navigate through their ocean environment. For white sharks in Australian and New Zealand waters, horizontal movement has been relatively well studied, but their diving behaviour is less well understood. We tagged 27 immature white sharks with three different tag types that collect positional, temperature and depth data. Tagged sharks travelled from southern Queensland to southern Tasmania and New Zealand. All sharks frequently dove up and down the water column (0–632 m) and experienced temperatures ranging from 7.8–28.9 °C. Importantly, there was a noticeable difference in the sharks’ diving behaviour between day and nighttime. During the day, white sharks remained at relatively constant depths, while at night, they constantly moved vertically through the water column in a high frequency. These findings help us to better understand how Eastern Australasian (EA) white sharks navigate through the ocean and provide new information on the ecology of juvenile white sharks.

**Abstract:**

Knowledge of the 3-dimensional space use of large marine predators is central to our understanding of ecosystem dynamics and for the development of management recommendations. Horizontal movements of white sharks, *Carcharodon carcharias*, in eastern Australian and New Zealand waters have been relatively well studied, yet vertical habitat use is less well understood. We dual-tagged 27 immature white sharks with Pop-Up Satellite Archival Transmitting (PSAT) and acoustic tags in New South Wales coastal shelf waters. In addition, 19 of these individuals were also fitted with Smart Position or Temperature Transmitting (SPOT) tags. PSATs of 12 sharks provided useable data; four tags were recovered, providing highly detailed archival data recorded at 3-s intervals. Horizontal movements ranged from southern Queensland to southern Tasmania and New Zealand. Sharks made extensive use of the water column (0–632 m) and experienced a broad range of temperatures (7.8–28.9 °C). Archival records revealed pronounced diel-patterns in distinct fine-scale oscillatory behaviour, with sharks occupying relatively constant depths during the day and exhibiting pronounced yo-yo diving behaviour (vertical zig-zag swimming through the water column) during the night. Our findings provide valuable new insights into the 3-dimensional space use of Eastern Australasian (EA) white sharks and contribute to the growing body on the general ecology of immature white sharks.

## 1. Introduction

Movement, defined as displacement of an organism from one place to another, is one of the key characteristics defining life [1]. A comprehensive understanding of the patterns, mechanisms, causes, and consequences of animal movement is fundamental to managing and restoring degraded ecosystems and conserving biodiversity [2]. In the face of worldwide decreasing marine predator populations, it is important to address the behavioural strategies of these animals and to understand the role of movement in determining the fate of individuals and the structure and dynamics of populations, communities, and entire ecosystems. Yet, despite extensive technological advances in aquatic telemetry [3], drivers and consequences of individual movement behaviour in oceanic habitats remain poorly understood for most species.

In juvenile and adult white sharks, *Carcharodon carcharias* (Linnaeus 1758), shelf-oriented movements and occasional oceanic migrations are well-documented globally [4,5,6]. Similarly, vertical movements of the species have been relatively well studied in the Pacific and Indian Ocean e.g., [5,7,8], and to a lesser extent in the Atlantic [9,10,11,12]. Reported dive and horizontal patterns show a wide range of inter-individual, geographic and temporal variation in both ocean- and shelf-phases and are temporally inconsistent, complicating the development and verification of hypotheses about their ecological role. A more cohesive understanding of the 3-dimensional movements of white sharks across all life stages has thus been identified as one of the top 10 research priorities for this species globally [13] and as a key research priority under a national recovery plan for white sharks in Australian waters [14].

White sharks are listed as threatened in Australia’s Environment Protection and Biodiversity Conservation Act of 1999 and as ‘Vulnerable’ and ‘Moderately Depleted’ globally, based on International Union for Conservation of Nature (IUCN) Red and Green List criteria, respectively [15,16]. The waters surrounding eastern Australia and New Zealand harbour a single population of white sharks [17], hereafter referred to as eastern Australasian (EA) white sharks. Juvenile and sub-adult (hereafter referred to immature) individuals of this population regularly frequent coastal habitats along the Australian east coast [6,18,19,20,21,22,23,24,25]. Movements of this population have been relatively well studied, yet most tagging studies have focused on horizontal rather than vertical patterns, paying little attention to the 3-dimensional nature of the marine environment. Satellite and acoustic tracking data revealed complex spatial dynamics comprising an inshore continental-shelf phase as well as extensive oceanic travel, resulting in connectivity across their entire Australasian range [6,18,19,20,21,22,23,24,25]. Vertical movements in coastal habitats were typically concentrated in the upper 100 m of the water column [6,21,25]. Yet, reported vertical behaviours were highly variable during oceanic travel, including bimodal depth distributions, deep-diving and surface-oriented swimming [6,26,27]. Similarly, reported diel-depth patterns of EA white sharks are inconsistent across and within individuals, ranging from highly diel-oriented behaviour to a complete lack thereof. Diel-oriented patterns identified by previous studies on juvenile, sub-adult, and adult EA white sharks have typically been limited to short periods (days) and were not well correlated with location. Observations of rapid switching of behaviour from one mode to another, even when sharks were in the same location, were common [6,24,26,27]. In contrast, archival data of adult white sharks in the Pacific revealed clear spatial patterning of diel vertical migration (DVM) behaviour [28,29]. Although most authors have attributed observed diving patterns to prey searching and navigation e.g., [10,26], drivers of vertical movement patterns in white sharks remain speculative across their circumglobal range.

To facilitate the recovery of white shark populations in Australasian waters and to further elucidate how white sharks use coastal as well as pelagic environments, additional empirical data and a more detailed examination of their complex movement behaviours is needed. Here, we integrated multiple complementary tag technologies and conventional fishery-dependent tag/recapture data (recapture location, time, and day) to characterize space use, temporal trends, and fine-scale vertical behaviour in relation to white shark ecology. We employed Pop-Up Satellite Archival Transmitting tags (PSATs), Smart Position or Temperature Transmitting (SPOT) tags, and acoustic tagging technologies to: (1) examine timing and extent of broad-scale horizontal movements; (2) identify regional and diel patterns in vertical movement patterns; and (3) determine intra- and inter-individual differences in fine-scale vertical behaviour. Here we provide an initial, description of this information-rich dataset. 

## 2. Materials and Methods

### 2.1. Tag Deployment

Twenty-seven white sharks were dual-tagged with PSATs (MiniPAT; Wildlife Computers Inc., Redmond, WA, USA) and V16-6L acoustic transmitters (InnovaSea, Marine Systems Canada, Inc., Halifax, Nova Scotia; transmission intervals 40–80 s, 10-year battery life). Nineteen of the 27 sharks were also tagged with SPOT tags (Wildlife Computers, Inc, Redmond, WA, USA) as part of a wider research program. Transmitters were fitted to sharks between 17 August 2017 and 27 July 2019. Tagging operations were conducted in NSW coastal shelf waters between Lennox Head (28.80° S 153.59° E) and Forster (32.18° S 152.51° E) within ~0.5 km of the coast. Sharks were caught using Shark Management Alert in Real Time (SMART) drumlines [30], brought alongside the boat and secured with belly and tail ropes following capture. Prior to release, sharks were sexed (based on the presence or absence of claspers) and measured to the nearest centimetre for fork length (FL) using a measuring tape stretched from the tip of the snout to the fork of the tail. Typically, sharks were restrained for less than 20 min before release. Each shark was assigned a unique ID number. Since all sharks in this study are part of a jurisdictional dataset, the numbering of individual sharks is not consecutive. Acoustic transmitters were either surgically implanted into the abdominal cavity following the general procedure of Heupel et al. [31] (*n* = 13) or externally attached by embedding nylon umbrella Domeier anchors into the dorsal musculature using applicator needles mounted on a hand-shaft (*n* = 12). Three sharks that were originally tagged internally were recaptured and fitted with an additional external acoustic transmitter as part of a wider tracking program. PSATs (12 cm length, volume: 60 cm^3^) were tethered using a 15 cm filament of 1.3 mm diameter stainless steel wire covered with black heat shrink and crimped at either end. On sharks that were also tagged with SPOT tags (*n* = 19), PSATs were deployed in two different ways; in conjunction with (*n* = 7) or separate from SPOT tags (*n* = 12). For the former, a template was used to make four holes (4 mm diameter) on the leading edge of the fin, as high up as possible while retaining support from the fin structure of each shark. Stainless steel bolts (4 mm × 50 mm) were then pushed through these holes. The lower right bolt was simultaneously passed through the tether loop of the MiniPAT and the device was secured on the opposite side of the fin with lockers and lock nuts (Figure S1A). For the latter, the PSATs were deployed either by pushing stainless steel bolts through a hole, ca. 2 cm from the base of the SPOT tag and securing the tag on the opposite side of the fin with a locker and a lock nut (*n* = 6) (Figure S1B), or by implanting a Domeier anchor into the basolateral dorsal musculature using a handheld tagging pole (*n* = 6) (Figure S1C). PSATs on sharks that were not tagged with SPOT tags were secured by the same methods; i.e., either lockers and nuts or Domeier anchors. PSATs were inserted at an angle of 45 towards the shark’s head, which ensured that the tag trailed behind the fin.

### 2.2. Tag Details and Programming

Acoustic signals were tracked by 11 arrays, each comprising up to 36 passive acoustic receivers (VR2W and VR4G; InnovaSea, Marine Systems Canada, Inc., Halifax, Nova Scotia), in 10 different regions off the east/south-east and west coast of Australia and the east coast of Tasmania, (see [23] for details on receiver locations, configurations and operational periods). SPOT tags were programmed to transmit location data to the Argos satellite constellation while at the surface a maximum of 250 times per day. PSATs were programmed to archive light intensity, water depth (±0.5 m) and temperature (±0.05 °C) data every 3-s and to release from the sharks after 180 d. The archival data was compressed into 24 h temporal bins for satellite data transmission.

### 2.3. Track Reconstruction

Geolocation protocols are detailed in Lipscombe et al. [32], and are briefly described here. Argos location estimates provided by SPOT tags were all opportunistic in nature, as a shark’s fin needed to break the sea surface long enough to communicate with an orbiting satellite. As a result, positional information was often clustered in space and time, yet varied over the course of a deployment. SPOT tag location class Z positions were considered invalid and were removed from further analysis [33]. Transmitted data from PSATs were decoded with the manufacturer’s cloud-based portal software. Positioned via light-based geolocation estimation, these tags offered the least accurate positions [34], yet given a shark’s occupancy in the epipelagic layer, good light-level readings were usually available throughout the deployment period. To estimate positions provided by PSATs, we performed a geolocation analysis using the manufacturer’s proprietary Hidden Markov Model (HMM, WC-GPE3, Global Position Estimator Program suite, Wildlife Computers, Redmond, WA, USA). Alongside the positions estimated from the archived PSAT datasets, available known locations from SPOT tags, recapture events and acoustic detections were also included in the model. Acoustic positions were usually obtained in clusters, as a shark tended to be detected by the same receiver at a particular location. To reduce such clustering, we only included a single daily acoustic position when multiple detections were made. Although most published white shark speed estimates range between 0.3 to 1.3 ms^−1^, we ran the HMM with 1, 2, 3, 4 and 5 ms^−1^ as the prior to avoid spatial constraint of model likelihoods should sharks in this study move a larger distance for any period of the track. Solution scores among models using different swimming speeds were similar, indicating model results were convergent. We hence set the speed filter at 5 ms^−1^. The final track for each shark was generated by selecting positions for each day under the following order of priority: (1) Argos/acoustic, (2) either dusk or dawn, (3) positions interpolated by the model. For each tagged shark, we estimated the distance travelled (in kilometres) by calculating Euclidean distance between waypoints.

### 2.4. Depth and Temperature Data Processing

Transmitted depth and temperature data were analysed using the R package ‘RchivalTag’ [35]. We defined time-series depth and temperature records as day or night, based on local time calculated by time difference from the Greenwich Mean Time, i.e., GMT + 10. Data were trimmed to reflect the time period when a shark was clearly carrying the tag. To investigate potential regional differences in depth occupation, we divided the study area into 3 latitudinal zones, based on the habitat use and residency patterns of juvenile Australasian white sharks established in [22]. Zone 1 extends from 25° S to 31° S and represents the northernmost area of the study region, characterised by an overall lower habitat use compared to the other two zones; Zone 2 extends from 31° S to 38° S, and includes a ~300 km stretch of coastline representing an extensive white shark nursery area [22,24]; Zone 3 extends from 38° S to 45° S across the Corner Inlet nursery area [24] and the eastern Bass Strait to waters south of Tasmania. The three zones together represent the main coastal habitats of the EA white shark population. 

### 2.5. Statistical Analysis

Descriptive statistics (mean, min, max, SD) were derived using base functions in R (version 4.0.5) [36]. To characterize changes in swimming depth, depths were averaged hourly over the 24-h period for each shark over the entire deployment period. As measure of temporal variability of swimming depth, a coefficient of variation was calculated on each recovered time series, which is a useful measurement of temporal variability because it is unitless and independent of the number of samples contained in a time series. 

## 3. Results

### 3.1. Deployment Summary

Based on the life history definitions provided by Bruce and Bradford [24], all 27 tagged sharks were immature at the time of tagging, ranging in fork length (FL) from 174 to 320 cm, with a mean of 228 ± 40 cm (SD) (Table 1). Fourteen sharks (52%) were females, 26 sharks were juveniles (155–280 cm FL; 13 females, 13 males), one shark was a sub-adult (281–350 cm FL; 1 female) (Table 1). Of the 27 deployed PSATs, 12 provided data (44%) (Table 1). Of these three released prematurely after 93, 140 and 146 d, respectively (Table 1). Thirteen tags did not report. In addition, one tag spontaneously deleted the entire archive after the tag was retrieved, and another one provided a pop-up location but no other useable data (Table 1). PSATs of sharks 180, 356, 365 and 366, were physically recovered, thus providing complete archived datasets. For each of those tags the archived data contained > 4 million time series data points for recorded depth, temperature, and light levels at 3-s intervals over the deployment period. Across all sharks for which PSAT data was received, a total of 3595 acoustic detections were retrieved throughout the receiver array. Deployment durations of the PSATs (*n* = 12) ranged from 93 to 180 d (mean = 164 d) and totalled 1965 tracking days during which individuals moved an estimated 11,573 km (mean = 8399 ± 3147 (SD) km; range 2989−12,673 km) and dove to a maximum depth of 632 m (4 ind. > 500 m) (Table 1). Of the 12 sharks for which data was received, eight were also tagged with SPOT tags, of which 100% provided data.

### 3.2. Horizontal Movements

**Figure 1:**
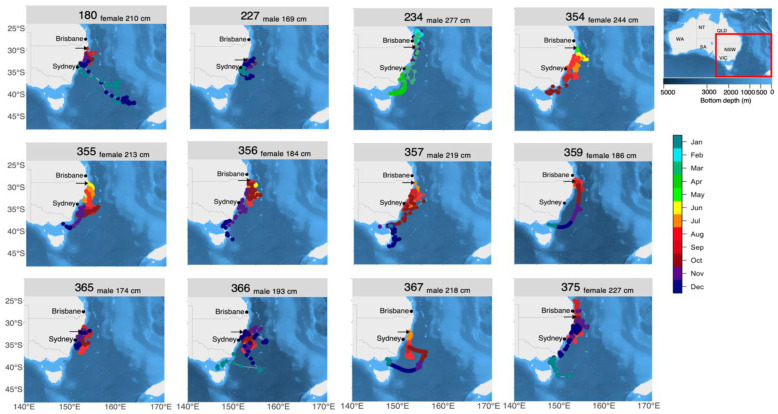
**Spatial scale of individual movements.** Interpolated telemetry tracks of 12 juvenile white sharks tagged off eastern Australia from a combination of satellite-linked radio, pop-up satellite archival, acoustic and recapture records collected between 12 September 2017 and 1 March 2020. Positions are colour-coded by month. Arrows indicate tagging locations. Red square indicates the study area. ID numbers, sex and fork length of tagged sharks are indicated above each map. All maps were generated using the marmap package in R [37].

Estimated latitudinal movements of PSAT tagged white sharks along the Australian east coast spanned from Fraser Island (25° S) to southern Tasmania (44° S) (Figure 1). The estimated longitudinal range generally extended from the coast to approximately 154° E (Figure 1). One of the tracked sharks (shark 180) travelled further southeast towards New Zealand’s South island, during the PSAT tracking period (Figure 1). It remained in waters approximately 500 km off southwest South Island New Zealand for 18 d, before travelling in a northern direction to an area west of the Lord Howe Rise, where it remained for approximately 18 d before returning to eastern Australia on a directed path.

Seasonal movement patterns were apparent (Figure S2) with tagged sharks dispersing north and south along the NSW coast between June and August during the austral winter. In the austral spring (September to November) they further extended their latitudinal range, which reached a maximum in December, with sharks occurring across ~2000 km of coastline from southern Queensland waters, along the NSW coast and into waters off southern Tasmania (Figure S2).

### 3.3. Vertical Activity

**Figure 2:**
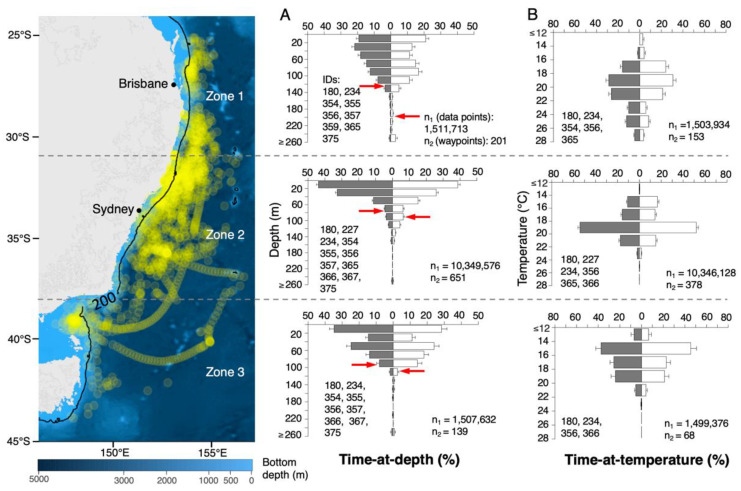
**Depth and temperature occupation by latitudinal zones (north, central and south).** Yellow dots represent positional data of all individual tracks (*n* = 12) displayed in Figure 1. (**A**,**B**) indicate percent time at depth and temperature by day (white bars) and night (grey bars) from Pop-Up Satellite Archival Transmitting tags (PSAT) data for each latitudinal zone. Error bars represent SDs. ‘IDs’ indicates the ID numbers of sharks for which depth and temperature records were included in each histogram. n_1_ and n_2_ indicate the number of depth and temperature records and the number of waypoints (points along estimated tracks) included in each histogram, respectively. Sharks for which depth or temperature data were missing for more than 25% of the total deployment days were excluded from the analyses. Red arrows indicate depth above which 95% of data occur for both day and night. Depth > 250 m were included into the 240–260 m bin. Temperatures < 12 °C were included into the 12–14 °C bin. For shark 180, all available depth and temperature data were included into the analysis, even though the full track of this shark is not shown in the zoomed-in map. The map was generated using the marmap package in R [37].

PSATs transmitted >1200 d of depth and >1000 d of temperature data, respectively. Based on recovered time-series data for sharks 180, 356, 365 and 366, mean and minimum depths per day were significantly deeper during the day than at night (mean depth: day 34.2 m ± 30.9, night 31.0 ± 26.2; Mann-Whitney rank-sum test, U = 3.9877 × 10^13^, n_1_ = 9,076,219, n_2_ = 8,780,746, *p* < 0.0001); (minimum depth: day 16.0 m ± 22.8, night 1.7 m ± 6.2; Mann-Whitney rank-sum test, U = 132,774, n_1_ = 8,780,746, n_2_ = 9,076,219, *p* < 0.0001), while a significant difference between maximum depth per day and night could not be demonstrated (day 55.0 m ± 34.79, night 55.36 ± 41.51) (Mann-Whitney rank-sum test, U = 209,677, n_1_ = 648, n_2_ = 646, *p* < 0.966). In general, sharks were surface-oriented, spending, on average, 22% and 81% of their time in the top 10 m and 50 m, respectively. Yet, depth preferences varied among individual sharks (Figure S3) and among regions (Figure 2).

Three regional depth patterns were identifiable: Region 1—broad distribution between the surface and 100 m; Region 2—most time (mean 76%) was spent in shallow water <40 m; Region 3—bimodal distribution, most time (mean 27%) was spent at the surface (0–20 m), with a second mode at 40–80 m (mean 25%). Water temperatures spanned a 21.1 °C range from 7.8–28.9 °C. Temperature ranges traversed by sharks continuously decreased from Zone 1 (7.8−28.9 °C) to Zone 2 (9.2–24.3 °C) to Zone 3 (16.3−22.1 °C).

**Figure 3:**
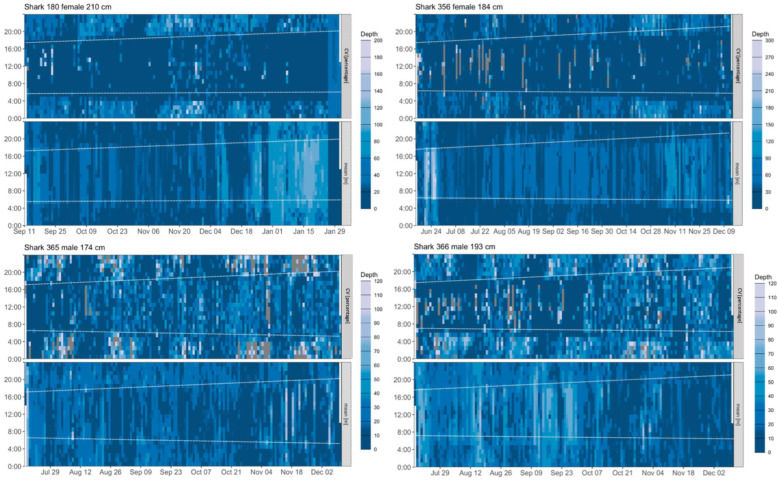
**Vertical activity analysis based on recovered tag data.** Coefficient of variation values and associated mean depth occupation for each of the four datasets displayed over 24 h for the entire tracking period for sharks 180, 356 and 365, 366. The same colour/numeric scale is shared by mean depth (in meters) and coefficient of variation in depth (expressed as a percentage). Dotted lines indicate times of local sunrise and sunset.

**Figure 4:**
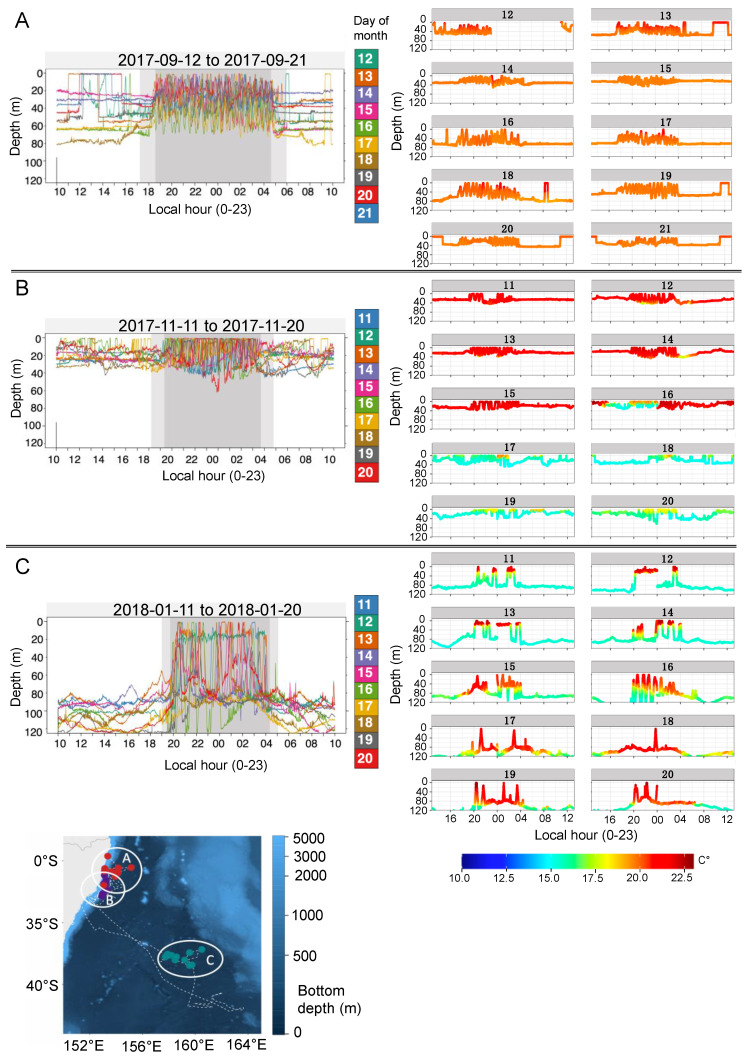
**Fine–scale diving behaviour.** Representative archival time-series depth and temperature data from shark 180 (210 cm FL, female). (**A**–**C**) Each plot on the left displays ten days of diving data collected at 3-s intervals and colour-coded by day. Areas shaded in grey indicate approximate night-time (darker grey) and twilight periods (lighter grey). (**A**–**C**) Plots on the right display the associated daily temperature records collected at 3-s intervals. Locations of track periods are indicated in the map. The map was generated using the marmap package in R [37]. Full archival time-series depth and temperature data for all four recovered archival tags are available in the supplement (Figure S6).

Archival depth and temperature data sampled at 3-s intervals revealed distinct fine-scale oscillatory behaviours for sharks 180, 356, 365 and 366 throughout the deployment records. These individuals primarily stayed at relatively constant depths throughout the day, with limited vertical movements. At night, they continuously oscillated up and down through the water column (as reflected by a high coefficient of variation) (Figure 3). Similar patterns were also visible in the transmitted data from non-recovered tags, except for 4 sharks that had insufficient amounts of depth data transmitted. In shark 180, distinct intra- and inter-regional variations in fine-scale vertical behaviour were visible (Figure 4). Within coastal shelf areas vertical behaviours included (1) multi-day periods during which the shark spent most of each day at a fairly constant depth level, with these constant depth levels varying among days from 20–80 m (Figure 4A), and (2) multi-day periods in which the shark occupied an overall shallower and narrower depth range (0–40 m) and showed an increased vertical activity during the day (Figure 4B). In offshore areas, the depth distribution during the day was generally deeper (80–120 m) compared to coastal habitats (Figure 4C). Nocturnal movements during all periods were characterised by an oscillatory or yo-yo swimming motion, during which the shark repeatedly ascended and less frequently descended and dived back to the respective daytime depth level (Figure 4A–C).

Of the 12 sharks that provided data, six (sharks 234, 354, 356, 357, 366, 375) dived to maximum depths of 424–632 m (Table 1). Deep-dives (beyond 200 m) occurred during all months of the year in 4 geographic clusters along the coast: (1) between Brisbane (27.47° S 153.02° E) and Fraser Island (25.23° S 153.13° E); (2) between Coffs Harbour (30.29° S 153.11° E) and Yamba (29.43° S 153.36° E); (3) offshore Jervis Bay (35.04° S 150.74° E) to the NSW Victoria border (37.50° S 149.97° E); (4) along the Tasmanian east coast (Figure S4). Deep-dives were initiated at all times of the day and night, with a peak in the morning between 0700–0800 h (25% of all deep-dives) (Figure S5).

## 4. Discussion

This study significantly expands our understanding of the 3-dimensional fine-scale movement ecology of immature white sharks along the Australian east coast. Based on a combination of satellite, acoustic and recapture data, we demonstrate complex horizontal and vertical movement patterns. These include unique diel behaviour, differing markedly from behavioural patterns described for juvenile and adult white sharks tracked in other regions around the globe, and pronounced spatial variability in depth preferences. Very fine-scale archival records (3-s archiving interval) provided the opportunity to examine vertical behaviour patterns that are challenging to infer from transmitted data summaries, including daily histograms. While elucidating the precise mechanisms driving these patterns is beyond the focus of this study, the results can be used as a basis for future research on, e.g., short duration behaviours, such as foraging events.

### 4.1. Horizontal Movements

Corroborating previous research on EA white sharks, tracked individuals spent most of their time in coastal waters, underlining the importance of the NSW coast for immature white sharks [18,22,23,24,38,39]. Contrary to previous studies suggesting on-shelf shark activity to be primarily shoreward of the 120-m depth contour [22,24], most positional estimates in our study were located further offshore (Figure 1 and map in Figure 2). Yet the large majority of these positions are likely an artefact resulting from the relatively high uncertainty exhibited by light-based geolocations (root mean square errors within ~80–150 km [40]). These positions should hence be treated with caution. During the PSAT tracking period the frequency of long-distance migrations was low (8%) but similar to that reported in previous tracking studies on EA white sharks [22]. Only one shark (shark 180) traversed from eastern Australia to waters southwest off New Zealand’s South Island, using one of the three north-south migratory corridors previously proposed for the EA white shark population [6,20,22]. While another shark (shark 234) also crossed the Tasman Sea, the transit occurred after the 180 d PSAT tracking period and is not presented here (but see [22]).

Consistent with previous tracking studies [18,22,23,24], seasonal movement patterns were evident, with peak abundances in NSW coastal areas from October through December (Figure S2). However, when interpreting these results, effects of tag deployment date and track duration must be considered. Deployment of tags was biased towards the months when white sharks were present in the tagging area; i.e., May–December (Table 1). Additionally, 57% of tags deployed in this study did not report. Having more than half of the total number of purchased tags as non-reporting was not only financially expensive but also resulted in significant loss of scientific opportunity, as has repeatedly been reported in PSAT studies e.g., [11,41,42]. In terms of seasonal analysis, it prevented us from attaining equal numbers of tracks per month, which would have been necessary to fully characterise seasonal patterns. The sharks that provided data were tracked for periods <1 year (93–180 d). The resulting variation in tracked individuals per month might have caused an upward bias on abundance for the months with the highest numbers of tracked individuals, i.e., October–December (11–12 individuals cf. 1 individual for the months February–May).

### 4.2. Diving Behaviour

Vertical movement patterns were complex, showing pronounced spatial variability in depth preferences and spatio-temporal shifts in behaviour. During continental-shelf phases, sharks spent most of their time between the surface and depths of 20–50 m. This corroborates previous research, suggesting that these depths are preferred by all life stages of the species across its range [4,6,7,10,11,21,24,25,26,43,44,45,46]. Habitat choice is largely the result of matching abiotic preferences, e.g., temperature, with ecological factors, such as prey availability, predation risk and competition. Juvenile EA white shark occurrence and movement patterns have previously been shown to be influenced by chlorophyll-a concentration (higher probability of occurrence in areas with moderate to high surface chl *a* concentration) and sea surface temperature (SST) (highest probability of occurrence at ~20 °C), but not by mesoscale eddies (in contrast to mature, female white sharks in the North Atlantic, which showed a high affinity to clockwise-rotating anticyclonic eddies) [39]. The observed variability in the vertical distribution of tracked sharks within and among individuals (Figure 4 and Figure S3) and among latitudinal areas along the Australian east coast (Figure 2) is likely driven by a complex of factors comprising the physiology of immature white sharks and abiotic and biotic environmental factors. Along the northern and central Australian east coast, tracked sharks displayed a preference for water between 18–20 °C, similar to previous studies in these regions [23,24,39]. Movement to lower latitudes coincided with the beginning of summer and increasing water temperatures along the Australian east coast. Yet, a tolerance for cooler water was evident through use of water between 14–16 °C south of 38° S, and occasional forays into the deep where temperatures as low as 7.8 °C were encountered.

An examination of high-resolution archival data revealed oscillatory dive behaviour with V-and U-shaped dives and pronounced diel changes in oscillatory movements. Repetitive oscillatory swimming is a common feature of the tracks of nearly all epipelagic top-order predators for which fine-scale depth traces have been recorded [47], including young-of-the-year, juvenile and adult white sharks e.g., [8,43,48]. Studies using biologging tags on tiger sharks (*Galeocerdo cuvier*) and oceanic whitetip sharks (*Carcharhinus longimanus*) suggested that these dives are likely related to prey searching behaviour [49,50,51]. Stomach content analyses suggest that teleost fishes are the predominant prey of EA immature white sharks [52], including pelagic eastern Australian salmon (*Arripis trutta*), which mainly occur in the upper 50 m of the water column [53]. Surface waters, which show significantly higher micronekton net biomass during the night than during the day along the Australian east coast [54], may hence attract sharks searching for silhouetted teleost prey on ascent. Foraging on benthopelagic batoids and an array of demersal and reef-associated species also seems to be common [52], and likely occurs during daytime based on the depth profiles observed in our study, which are similar to those reported for other species documented to be at the bottom, including white sharks [43,55,56].

High frequency oscillations during the night coupled with an occupation of relatively constant depths during the day is a unique behaviour displayed by our tracked sharks. This depth pattern differs from those of juvenile and adult white sharks tracked in other regions, which demonstrated deeper vertical oscillatory movements during the day rather than during the night [8,48,57]. Oscillatory behaviour of young-of-the-year (YOY) and juvenile white sharks in the northwest Atlantic, for example, was restricted to depths of ~30–50 m and lacked any clear diel patterns [44]. Archival data of YOY and juvenile white sharks in the eastern Pacific revealed diel patterns opposite to those described in our study, with sharks conducting high frequency oscillations during the day, while occupying surface mixed layers during the night [48,57]. In our study, nocturnal high frequency oscillatory movements and relatively constant depth during the day (including occasional ascents to the surface followed by surface swimming periods of up to 3 h) were characteristic of the entire tracking period in all sharks and did not noticeably change even during across-ocean migrations (see shark 180, Figure 4). The consistency in this behaviour across individuals and tracking periods might indicate that individuality in dietary preferences and foraging behaviour, as observed in adult white sharks [58,59], may develop later in life.

In our study, deep-diving behaviour was observed infrequently in six sharks, suggesting that individual sharks employ diverse strategies for deep pelagic habitat use, which varies across space and time. Mesopelagic excursions or occupation of deep pelagic habitats are common features of nearly all oceanic taxa studied with electronic tags [60]. Although direct evidence remains sparse, most shark tracking studies suggest deep-diving behaviour to be largely related to foraging, often within and below sound scattering layers e.g., [28,61,62]. The importance of mesopelagic prey for white sharks has recently been established in the Mexican Pacific using mercury isotopes yet considering that sharks could potentially access mesopelagic prey that migrate to the surface, direct evidence for the consumption of mesopelagic prey currently does not exist [63]. To clearly link mesopelagic excursions and successful foraging, future studies should leverage innovative tag technologies alongside emerging tools for studying food webs, e.g., ecological tracers such as Amino-Acid Compound-Specific Isotope Analysis (AA-CSIA) [63], trace elements [64], or radiogenic isotope analysis [65], The most likely alternative explanation for deep-diving behaviour suggests that large marine predators use occasional forays into the deep to probe the water column for navigational cues, e.g., geophysical gradients such as magnetic field intensity and/or inclination [66]. Such gradients may be up to 25 times stronger vertically than horizontally [60,67]. While in reality, it is still unknown whether sharks use information from the Earth’s field for navigation, results of several studies indicate that migratory sharks have the sensory capabilities to detect geomagnetic elements and use deep dives to orient to a change in geomagnetic intensity gradient [68,69]. Deep dives occurred in offshore waters along the eastern coasts of Australia and Tasmania (Figure S4). The angle of geomagnetic inclination contour lines in this region intersects at 30° (in the NE direction). Although the majority of deep-dive locations in our dataset are subjected to the limitations of light-based geolocations, most of them either align with the major contour lines or build clusters around them (Figure S4). If sharks are able to detect magnetic declination, predictable variation in inclination angle may allow them to maintain a steady compass heading and to determine whether their position is north or south of a particular area [70]. However, the infrequent occurrence of deep dives suggests that navigation via geomagnetic topotaxis is likely only a response to exceptional circumstances, e.g., before the onset of a directed migratory movement or a change from the current course of travel. Deep dive initiation times peaked in the early morning hours after dawn, suggesting that at least a portion of deep-dives might be triggered by environmental cues or a circadian clock. Twilight activity peaks have been observed in various large pelagic fishes, e.g., tunas (*Thunnini*) [71,72,73], mako sharks (*Isurus oxyrinchus*) [74], and billfishes (*Istiompax indica*) [74], and may be associated with foraging on vertically migrating prey species [75,76,77]. Future studies on white sharks would benefit from the use of stomach temperature data [78] or animal-borne imaging systems [79] to investigate a potential ecological importance of this behaviour and confirm whether it is indeed related to feeding activities.

While previous studies have demonstrated ontogenetic variation in deep-diving behaviour [80], the body size range in our dataset is insufficient to investigate whether variation in deep-diving behaviour is based on among-individual variation or ontogeny. To fully explore the role that deep-diving plays in the navigation of sharks, inter-study and inter-specific comparisons and meta-analysis of deep-diving behaviours, alongside significant advances in our understanding of sensory biology and technological developments in animal-borne sensors, will be necessary [81].

## 5. Conclusions

Our tracking results provide a more complete understanding of the 3-dimensional habitat use of immature white sharks in eastern Australian and New Zealand waters. Owing to the very fine-scale PSAT sampling interval used and the recovery of four archival tags, we were able to establish so far unreported consistent vertical diel patterns among tracked individuals. To determine whether the observed patterns are characteristic of all age classes within the EA white shark population, tagging of adult EA white sharks with high-sampling interval PSATs will be required. Overall, our results provide a sound basis for future studies disentangling the drivers behind the observed movement patterns, which will be critical for future conservation and management efforts.

## Figures and Tables

**Table 1:** Details and tracking periods of 27 tagged white sharks. Total time at liberty refers to the time period between the date of tagging and last detection by any of the three tag types (MiniPat tags [PSAT]; Spot–6 tags [Satellite Linked Radio Transmitting tags—SLRT]; acoustic tags), values in brackets indicate tracking period of successful PSAT deployments. FL: fork length; n/a: not applicable (shark was not fitted with tag type). ND: No detections recorded. Shading indicates sharks that were excluded from the analysis due to failed PSAT reporting; * spontaneous deletion of archive after tag was retrieved; ** only one transmission.

Shark ID	PSAT MiniPat Serial #	SLRT [Spot-6] Serial #	Acoustic Serial #	FL [cm]	Sex	Release Date	Release Latitude [° S]	Release Longitude [° E]	Pop Off Latitude [° S]	Pop Off Longitude [° E]	Pop Off Date	First Detection		Last Detection		Time at Liberty [d]	Depth Max. [m]	Temp Min. [°C]	Temp Max. [°C]
												SLRT	Acoustic	SLRT	Acoustic				
60	16P2399	16U0795	1241668	320	F	2018-11-16	−28.84	153.59	−	-	-	ND	2016-12-01	ND	2018-01-23	298 [-]	-	-	-
106	16P2195	16U2270	1241647	247	F	2017-08-17	−32.16	152.52	−	-	-	2017-08-14	2017-06-21	2018-02-26	2017-11-04	193 [-]	-	-	-
160	16P2288	16U2391	1268708	235	M	2017-08-17	−32.18	152.55	−	-	-	2017-08-17	ND	2019-02-14	ND	546 [-]	-	-	-
179	16P2362	16U2771	1268684	250	M	2017-09-13	−29.49	153.37	−	-	-	2017-11-17	2017-10-09	2019-06-18	2017-10-09	644 [-]	-	-	-
180	16P2232	12S1390	1241628	210	F	2017-09-12	−29.45	153.37	−33.53	151.37	2018-02-02	2017-09-12	2019-10-19	2018-01-08	2020-02-15	886 [140]	136	14.7	24.8
227	16P2397	12S1393	1268704	269	M	2017-10-24	−32.17	152.52	−33.91	151.51	2018-01-25	2017-10-24	2017-11-24	2018-03-22	2019-07-12	595 [93]	188	13.1	23.4
229	16P2400	16U2392	1268678	224	M	2017-10-25	−29.11	153.45	−	-	-	2017-11-01	2017-12-03	2018-09-05	2017-12-03	316 [-]	-	-	-
234	16P2189	16U2387	1268683	277	M	2017-10-28	−28.89	153.59	−35.13	150.87	2018-04-26	2017-11-20	2018-08-22	2019-04-18	2019-10-14	726 [180]	600	8.6	28.9
251 *	16P2398	12S1391	1268705	224	M	2017-11-15	−31.83	152.75	−32.77	152.24	2017-12-28	2017-11-15	2017-12-25	2018-01-10	2018-03-31	137 [-]	-	-	-
290	16P2203	16U2390	1241625	252	M	2018-07-27	−30.33	153.14	−	-	-	2018-09-18	2018-09-18	2019-02-09	2018-09-18	197 [-]	-	-	-
315	16P2314	16U2706	1268672	176	M	2018-08-29	−29.10	153.45	−	-	-	2018-08-29	2018-08-16	2019-07-30	2019-08-16	352 [-]	-	-	-
338	16P2186	n/a	1277240	236	F	2018-11-11	−29.08	153.45	−	-	-	n/a	2019-08-18	n/a	2019-08-18	337 [-]	-	-	-
341	16P2248	16U2393	1268671	252	F	2018-12-04	−31.81	152.75	−	-	-	2018-12-04	2018-12-12	2019-06-09	2020-01-31	423 [-]	-	-	-
343	16P2204	n/a	1268695	175	M	2018-12-04	−32.16	152.51	−	-	-	n/a	2019-05-03	n/a	2020-05-20	533 [-]	-	-	-
345	16P2190	n/a	1241614	202	F	2018-12-05	−32.16	152.51	−	-	-	n/a	2018-12-29	n/a	2020-02-19	441 [-]	-	-	-
354	18P1880	n/a	1304664	244	F	2019-05-30	−29.10	153.45	−37.84	148.46	2019-10-22	n/a	2019-07-03	n/a	2019-08-25	146 [146]	520	9.6	23.5
355	18P1877	n/a	1304663	213	F	2019-06-11	−29.11	153.46	−38.38	147.97	2019-12-07	n/a	2019-08-25	n/a	2019-08-25	180 [180]	128	14.2	22.5
356	18P1950	n/a	1304668	184	F	2019-06-16	−28.83	153.61	−41.23	148.70	2019-12-13	n/a	2019-06-16	n/a	2019-11-03	140 [180]	424	10.8	22.9
357	18P1881	n/a	1304673	219	M	2019-07-09	−29.08	153.45	−39.20	148.43	2020-01-05	n/a	2019-08-13	n/a	2020-06-19	346 [180]	632	8.1	21.6
358	18P1486	16U2769	1304630	260	F	2019-07-11	−28.79	153.61	−	-	-	2018-04-19	2019-10-14	2020-05-27	2019-10-18	322 [-]	-	-	-
359	18P1485	16U1461	1304633	186	F	2019-07-11	−28.88	153.61	−38.74	147.69	2020-02-10	2018-12-03	2019-09-19	2020-05-19	2019-09-19	313 [180]	79.5	19.0	21.5
360 **	18P1879	n/a	1304674	230	F	2019-07-12	−29.09	153.44	−47.68	122.13	-	n/a	2019-09-29	n/a	2019-10-03	84 [-]	-	-	-
364	18P1876	19U0148	1268692	177	F	2019-07-18	−32.16	152.52	−	-	-	2019-07-18	2019-07-30	2020-06-26	2019-08-25	345 [-]	-	-	-
365	18P1878	16U2701	1241627	174	M	2019-07-18	−32.16	152.51	−32.17	152.52	2019-12-11	2019-11-07	2019-07-28	2019-09-04	2020-03-06	232 [146]	116	14.2	29.3
366	18P1951	16U2704	1268679	193	M	2019-07-18	−32.17	152.52	−37.05	150.13	2020-01-16	2019-07-18	2019-08-08	2019-11-07	2020-03-11	238 [180]	528	9.9	23.6
367	18P1873	16U2516	1268679	218	M	2019-07-18	−32.16	152.51	−38.04	147.64	2020-01-14	2019-07-08	2019-08-11	2019-08-20	2020-06-19	337 [180]	144	12.8	20.6
375	18P1875	16U2388	1304649	227	F	2019-07-27	−28.86	153.61	−42.05	152.77	2020-03-01	2019-07-31	2019-10-12	2019-12-30	2019-12-19	156 [180]	568	7.8	24.5

## Data Availability

The datasets used and/or analysed during the current study are available from the corresponding author on reasonable request.

## Supplementary Materials

The following supporting information can be downloaded at: https://www.mdpi.com/article/10.3390/biology11121689/s1, Figure S1: PSAT attachment methods; Figure S2: Spatial scale of individual movements by month; Figure S3: Depth occupation of four sharks from recovered PSAT data; Figure S4: Deep dive locations (depth > 200 m). Figure S5: Frequency of deep dive initiation times. Figure S6: Dive profiles.
