# Supplementary material for: Spatial Dynamics and Fine-Scale Vertical Behaviour of Immature Eastern Australasian White Sharks (Carcharodon carcharias)
Source: Biology (Basel). 2022 Nov 22;11(12):1689. doi: 10.3390/biology11121689 (PMC9774733; doi:10.3390/biology11121689)
Supplement: Supplementary file 1 [file biology-11-01689-s001.zip › biology-1985814-supplementary.pdf]

Additional file 1

The following supplement accompanies the article:

Spatial dynamics and fine-scale vertical behaviour of immature Australasian white sharks (*Carcharodon carcharias*)

Julia L.Y. Spaet[1, 2*], Paul A. Butcher[2,3], A. Manica[1], Chi Hin Lam[4]

[1]Evolutionary Ecology Group, Department of Zoology, University of Cambridge, Downing Street, Cambridge CB2 3EJ, UK
[2]Southern Cross University, Coffs Harbour, NSW 2450, Australia
[3] Fisheries NSW, NSW Department of Primary Industries, National Marine Science Centre, Coffs Harbour, NSW 2450, Australia
[4] Large Pelagics Research Center, Gloucester, MA 01931, USA

* Corresponding Author: juliaspaet@gmail.com

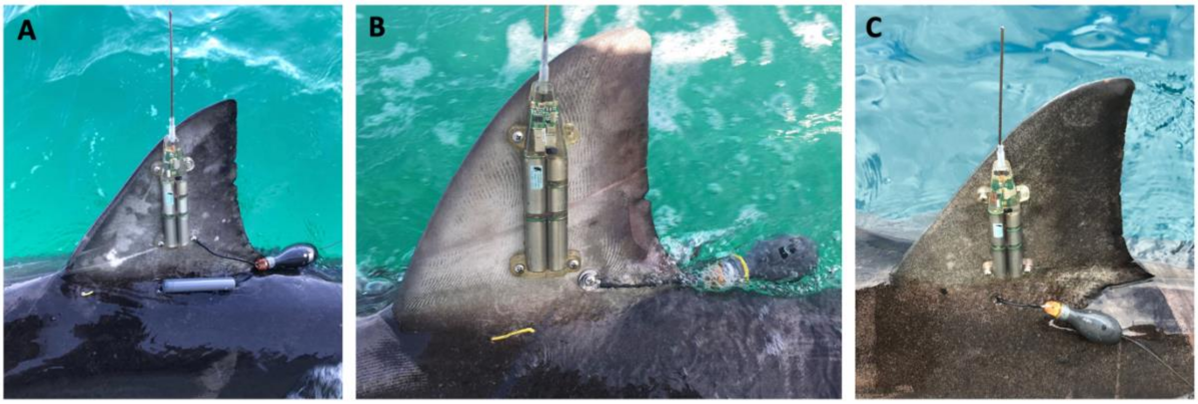

Figure S1. PSAT attachment methods.
(A) in conjunction with SPOT tags (n=7). (B) Separate from SPOT tags using stainless steel bolts (n = 6). (C) Separate from SPOT tags by implanting a 5 cm titanium plate 5 cm into the basolateral dorsal musculature (n = 14).

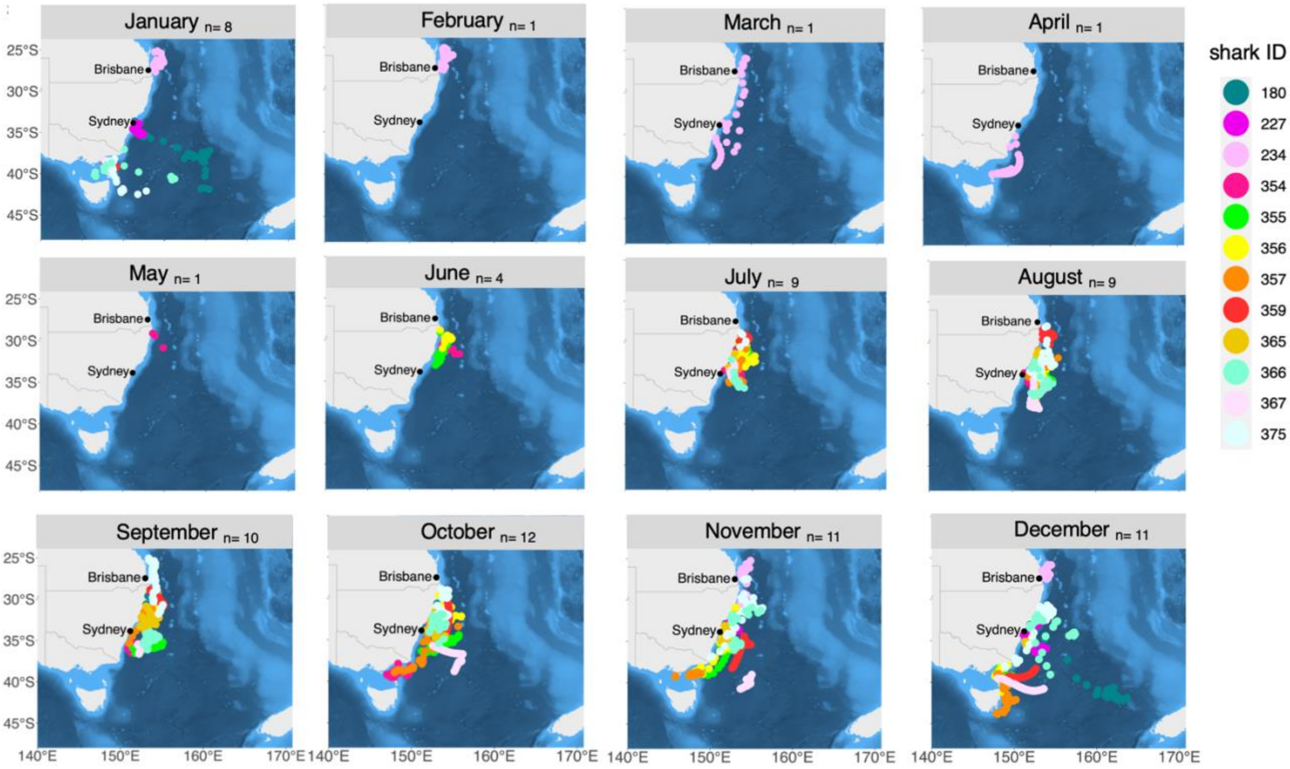

Figure S2. Spatial scale of individual movements by month. Interpolated telemetry tracks of 12 juvenile white sharks tagged off eastern Australia from a combination of satellite-linked radio, pop-up satellite archival, acoustic and recapture records collected between 12 September 2017 and 01 March 2020. Positions are colour-coded by individual. n = the number of individual tracks displayed in each monthly plot. Maps were generated using the marmap package in R (Pante & Simon-Bouhet 2013).

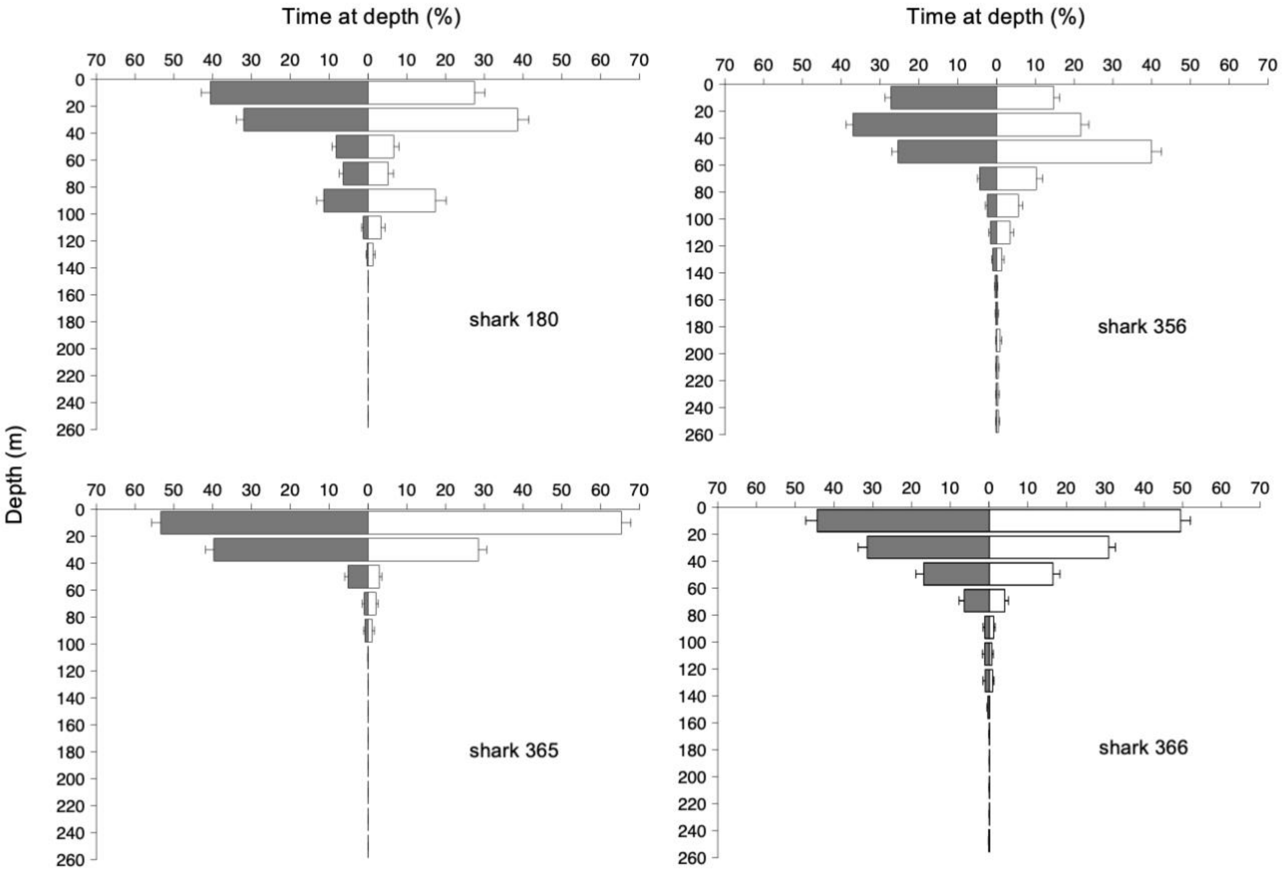

Figure S3. Depth occupation of 4 sharks from recovered PSAT data. Percent time at depth across the entire study area from recovered PSAT data from sharks 180, 356, 365 and 366. Day and night are indicated by white and grey bars, respectively. Depth > 250 m were included into the 240 – 260 m bin.

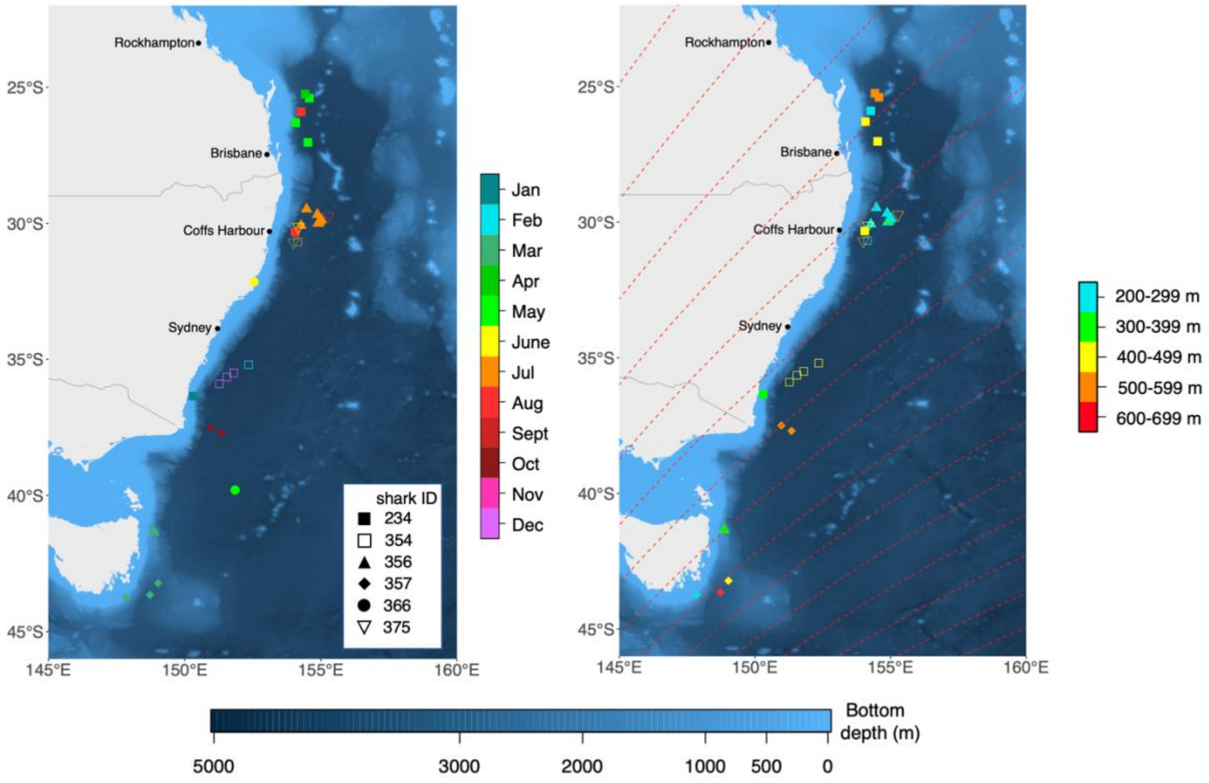

Figure S4. Deep dive locations (depth > 200m). (A) Colour coded by month. (B) Colour coded by depth. Dashed lines in red indicate isolines for magnetic declination. Maps were generated using the marmap package in R (Pante & Simon-Bouhet 2013).

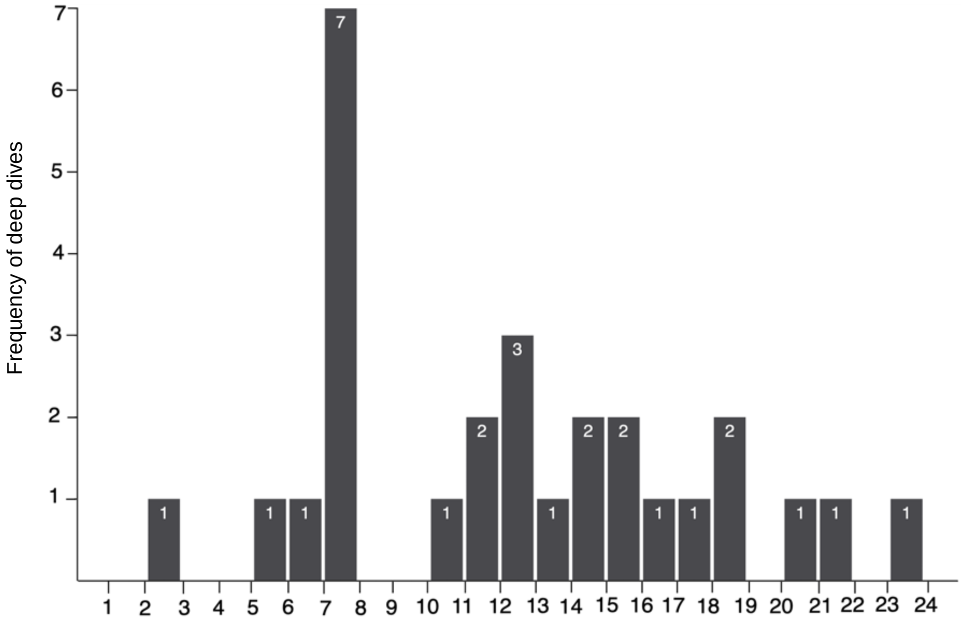

Figure S5. Frequency of deep dive initiation times. Approximate local deep dive (> 200m) initiation times for 6 sharks (234, 354, 356, 357, 366, 375).

Shark 180

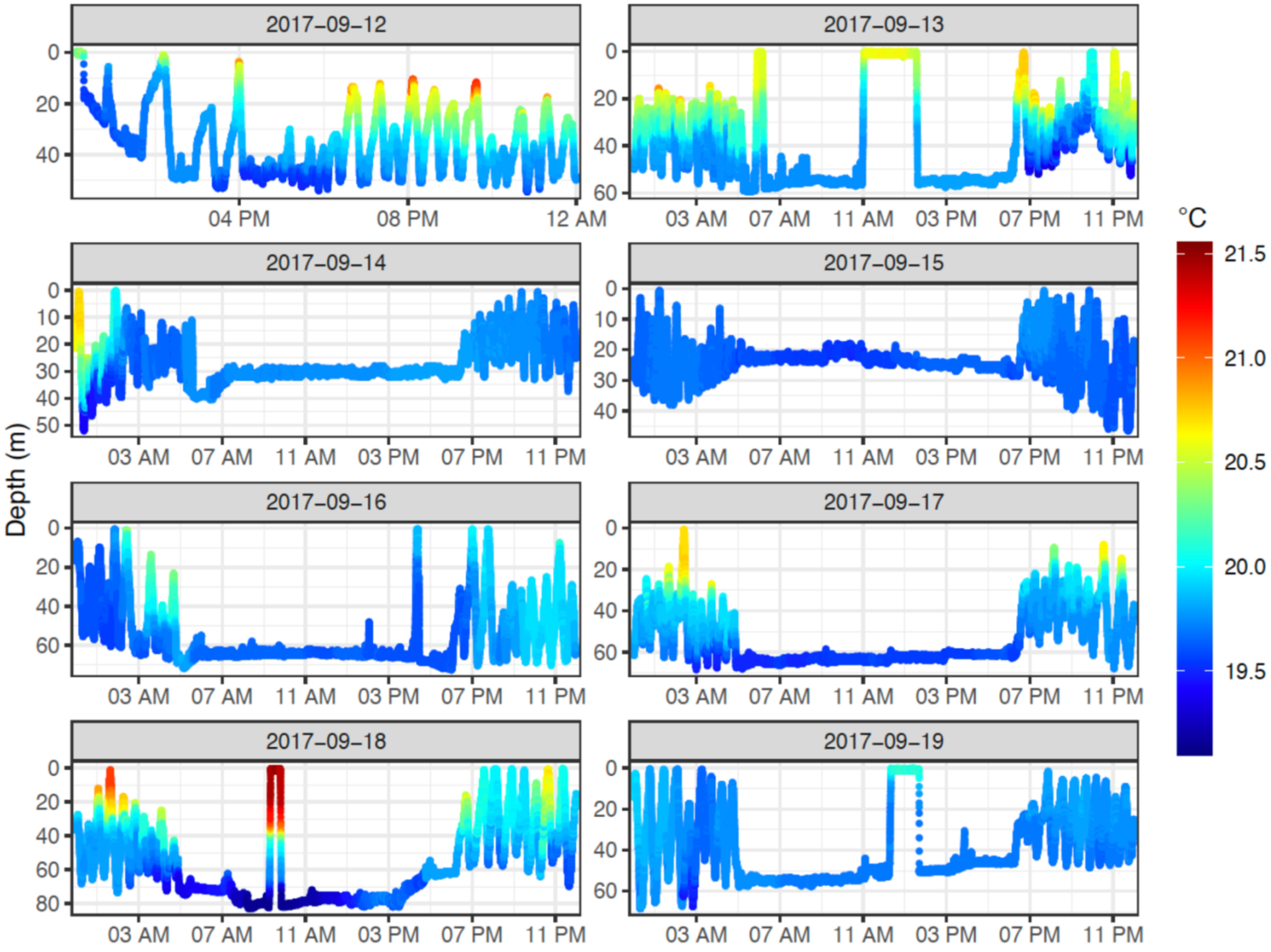
2017–09–12
2017–09–13
2017–09–14
2017–09–15
2017–09–16
2017–09–17
2017–09–18
2017–09–19
Depth (m)
04 PM
08 PM
12 AM
03 AM
07 AM
11 AM
03 PM
07 PM
11 PM
°C
21.5
21.0
20.5
20.0
19.5

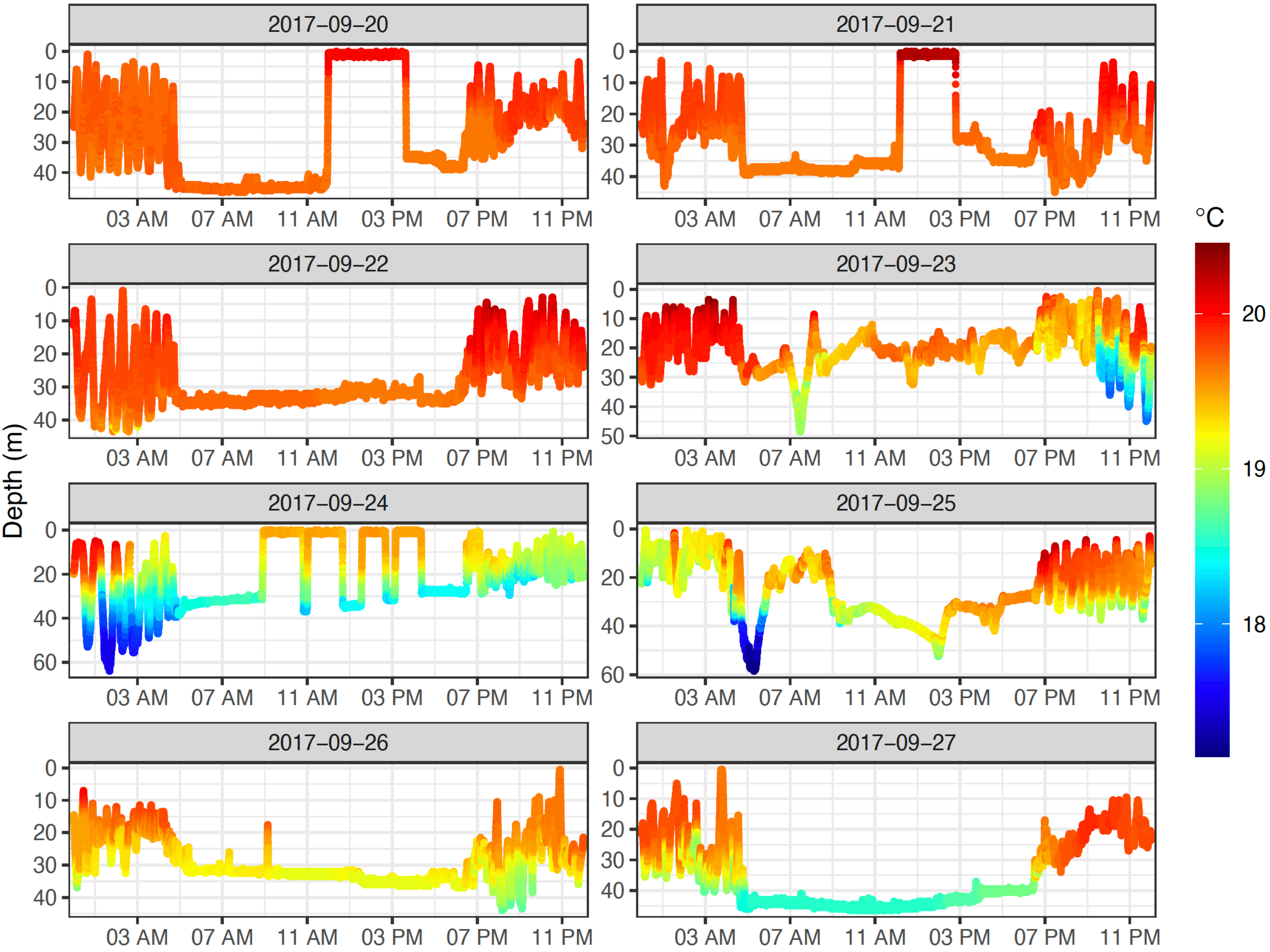
2017−09−20
2017−09−21
2017−09−22
2017−09−23
2017−09−24
2017−09−25
2017−09−26
2017−09−27
Depth (m)
°C
20
19
18
0
10
20
30
40
50
60
03 AM
07 AM
11 AM
03 PM
07 PM
11 PM

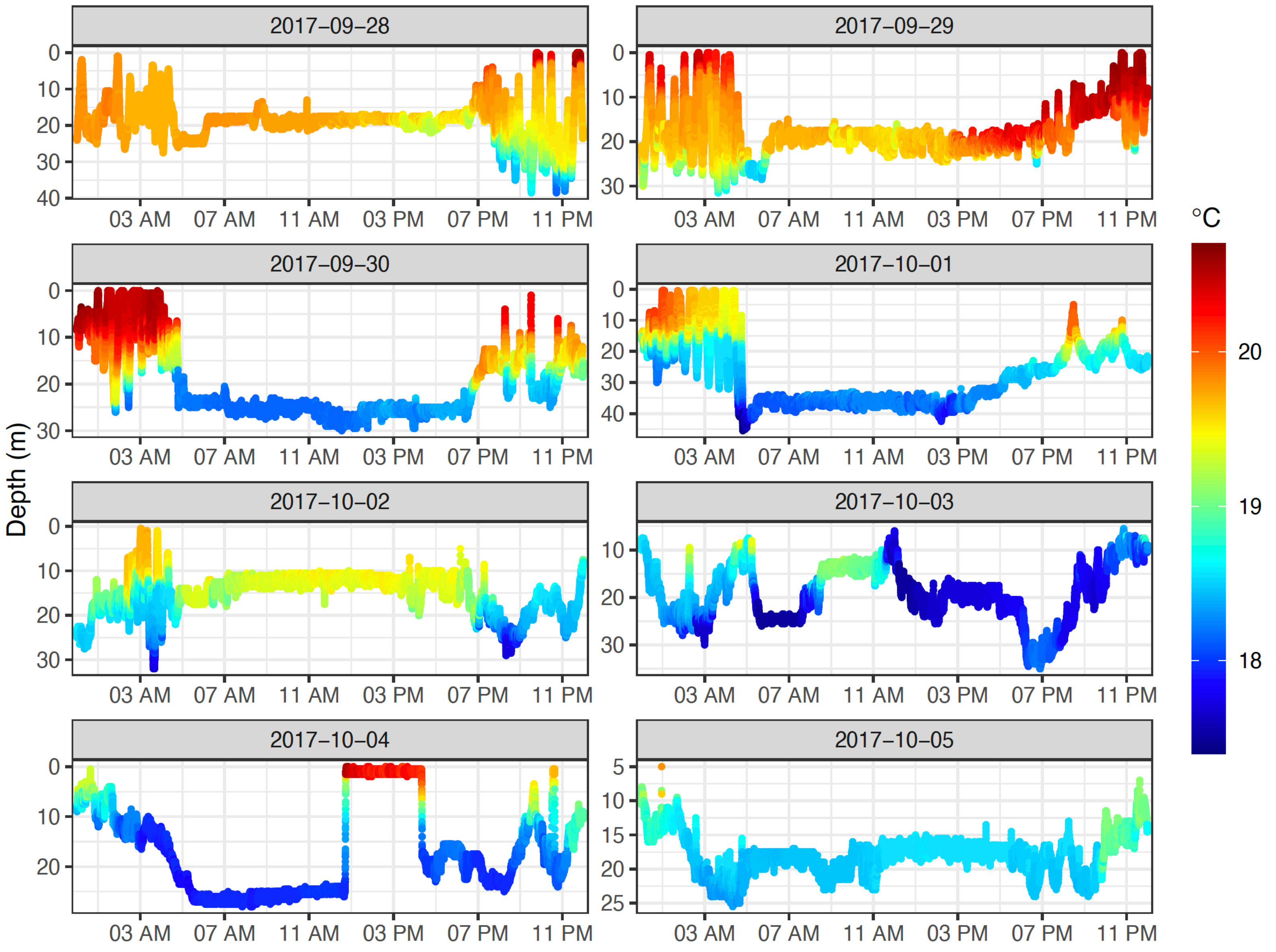

2017−09−28
2017−09−29
2017−09−30
2017−10−01
2017−10−02
2017−10−03
2017−10−04
2017−10−05
Depth (m)
03 AM
07 AM
11 AM
03 PM
07 PM
11 PM
°C
20
19
18

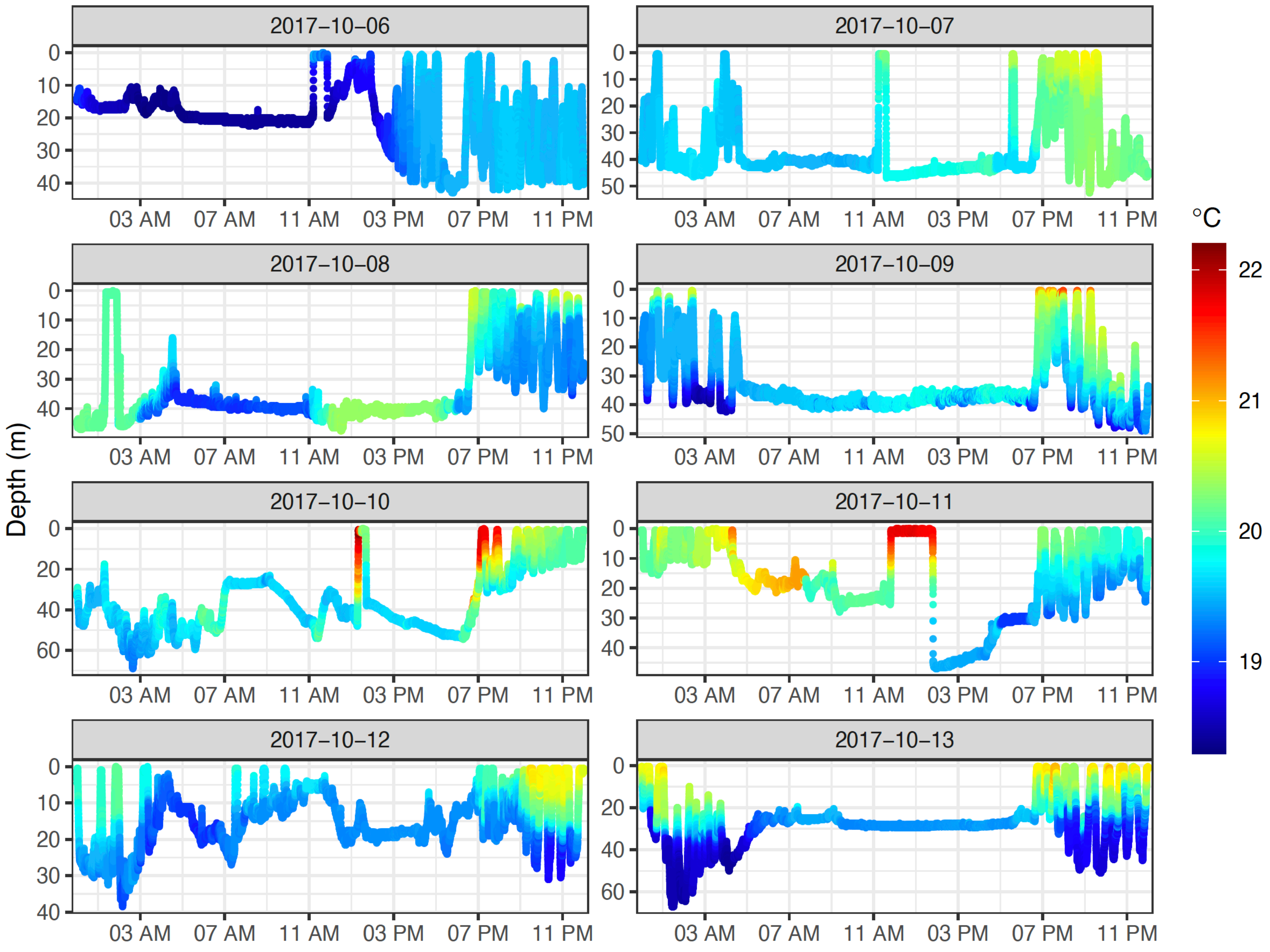

2017−10−06
2017−10−07
2017−10−08
2017−10−09
2017−10−10
2017−10−11
2017−10−12
2017−10−13
Depth (m)
03 AM
07 AM
11 AM
03 PM
07 PM
11 PM
°C
22
21
20
19

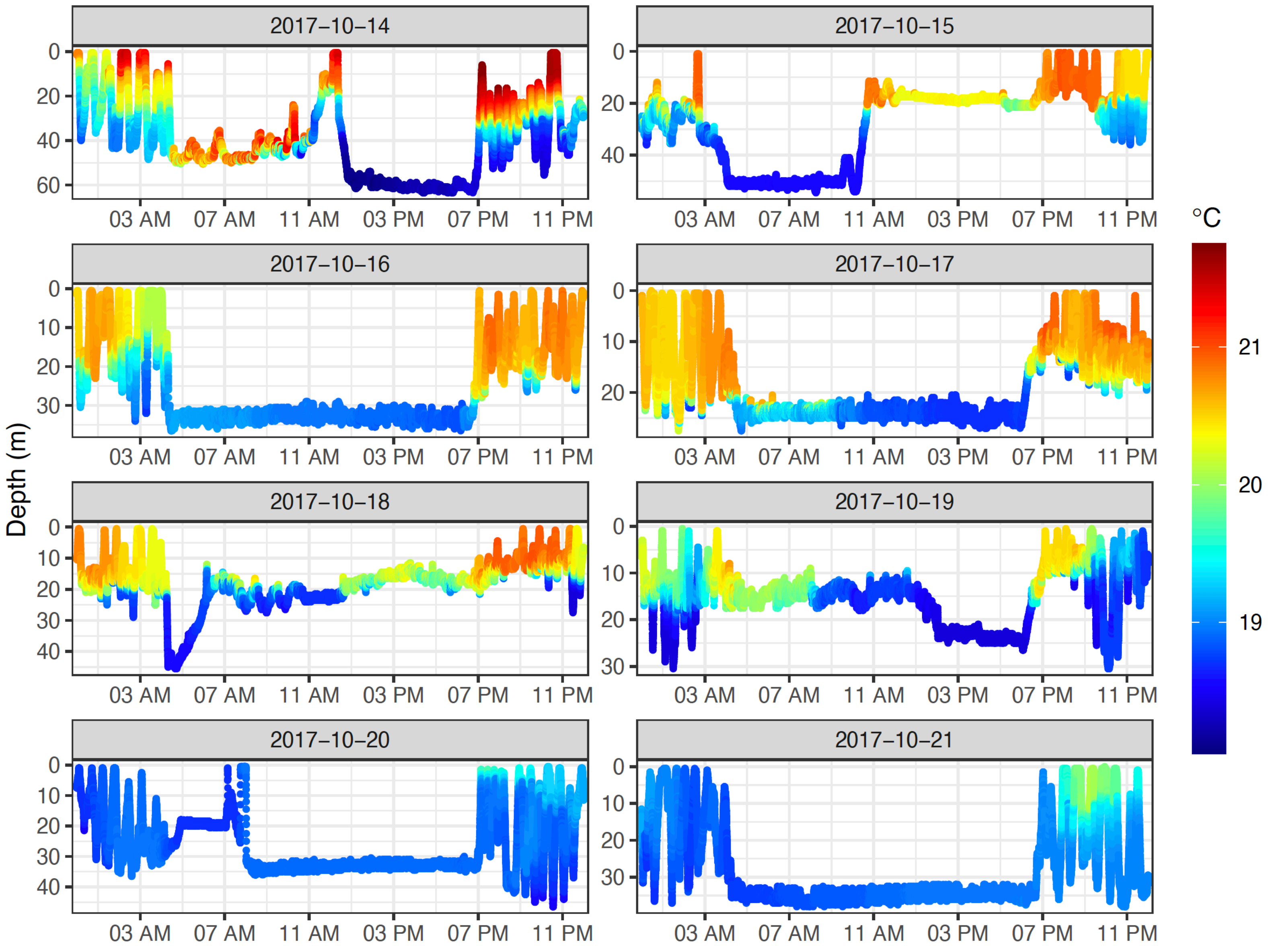

2017−10−14
2017−10−15
2017−10−16
2017−10−17
2017−10−18
2017−10−19
2017−10−20
2017−10−21
Depth (m)
03 AM
07 AM
11 AM
03 PM
07 PM
11 PM
°C
21
20
19

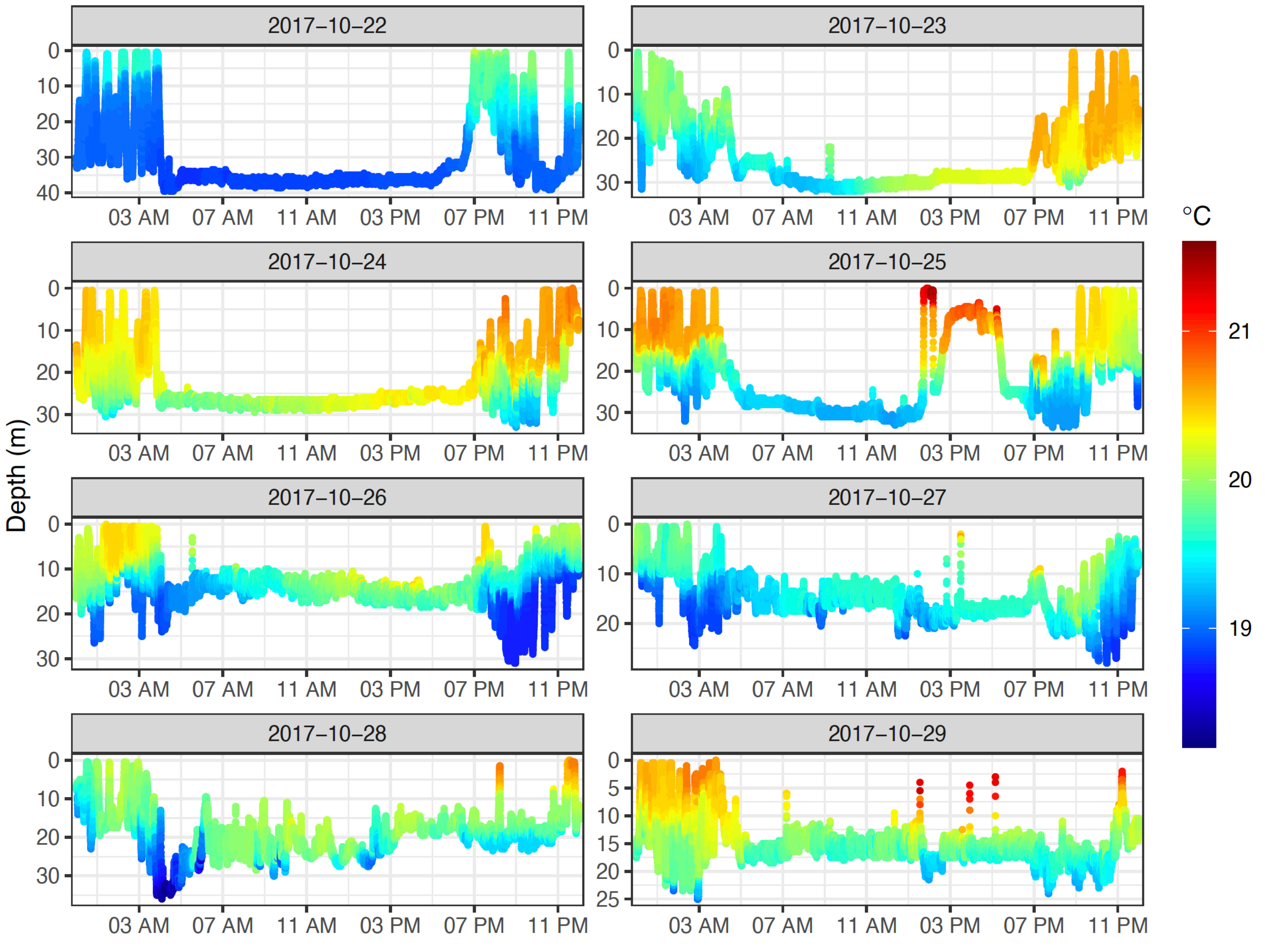

2017–10–22
2017–10–23
2017–10–24
2017–10–25
2017–10–26
2017–10–27
2017–10–28
2017–10–29
Depth (m)
03 AM
07 AM
11 AM
03 PM
07 PM
11 PM
°C
21
20
19

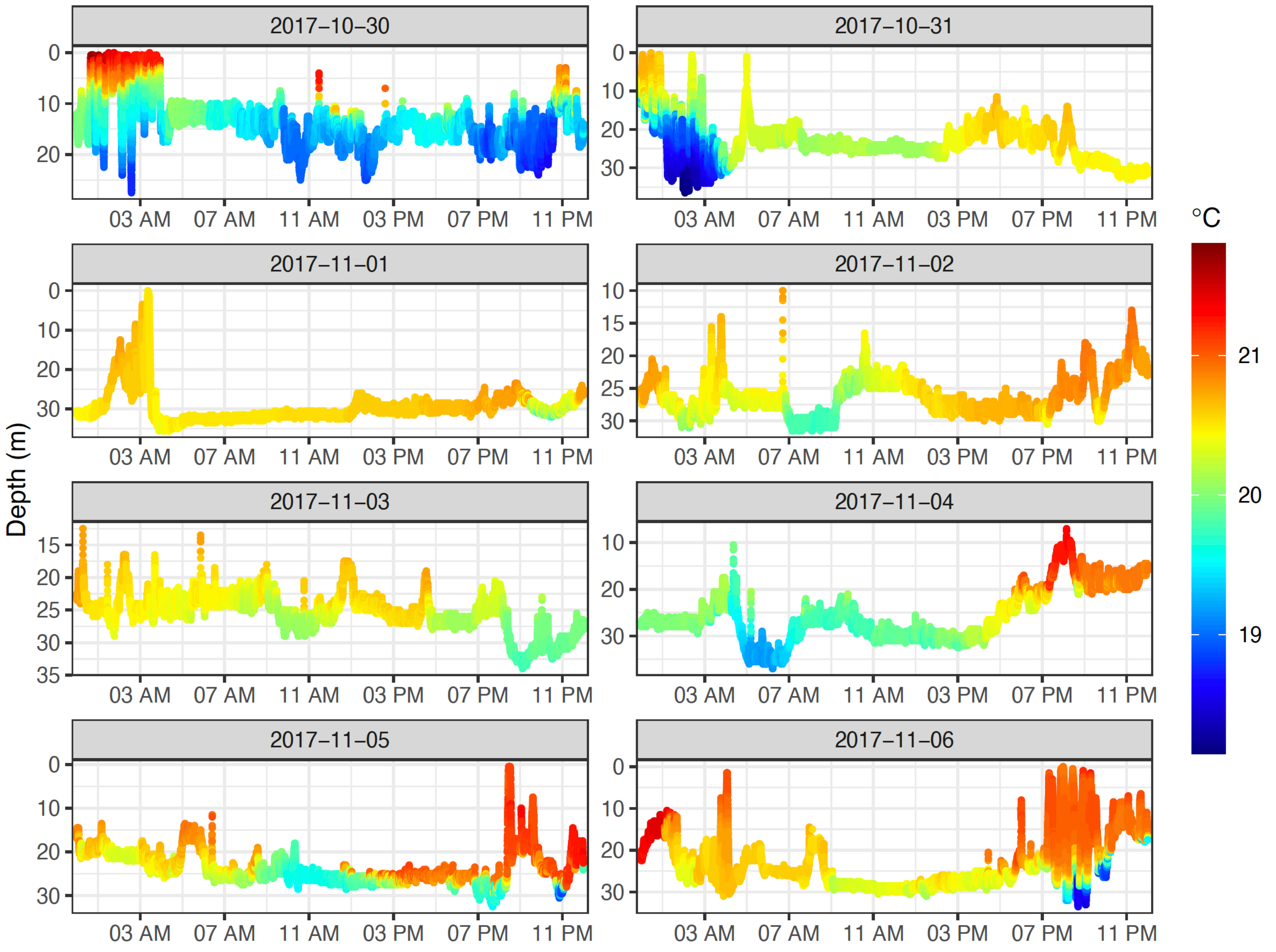

2017–10–30
2017–10–31
2017–11–01
2017–11–02
2017–11–03
2017–11–04
2017–11–05
2017–11–06
03 AM
07 AM
11 AM
03 PM
07 PM
11 PM
Depth (m)
°C
21
20
19

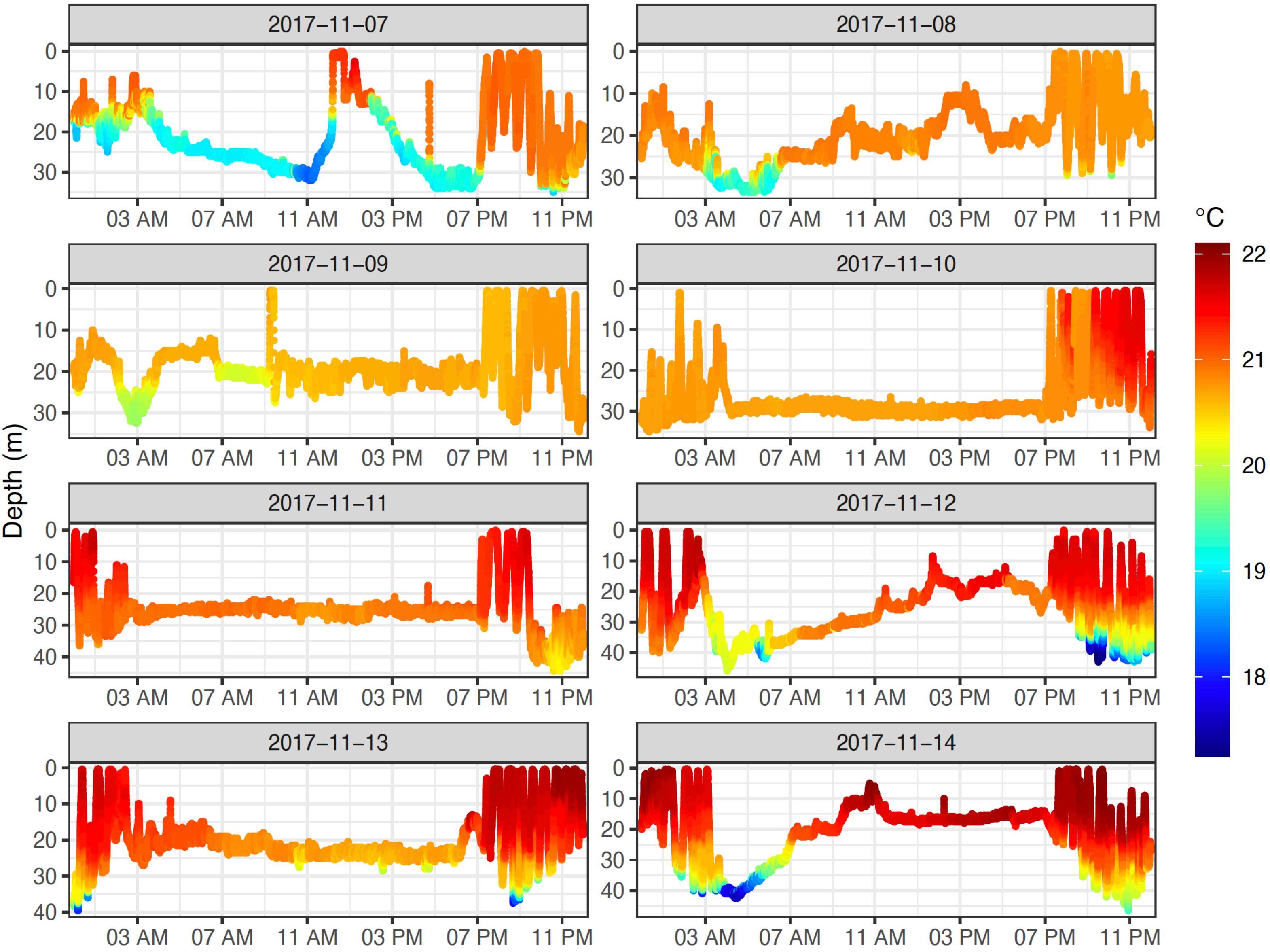
2017−11−07
2017−11−08
2017−11−09
2017−11−10
2017−11−11
2017−11−12
2017−11−13
2017−11−14
Depth (m)
03 AM
07 AM
11 AM
03 PM
07 PM
11 PM
°C
22
21
20
19
18

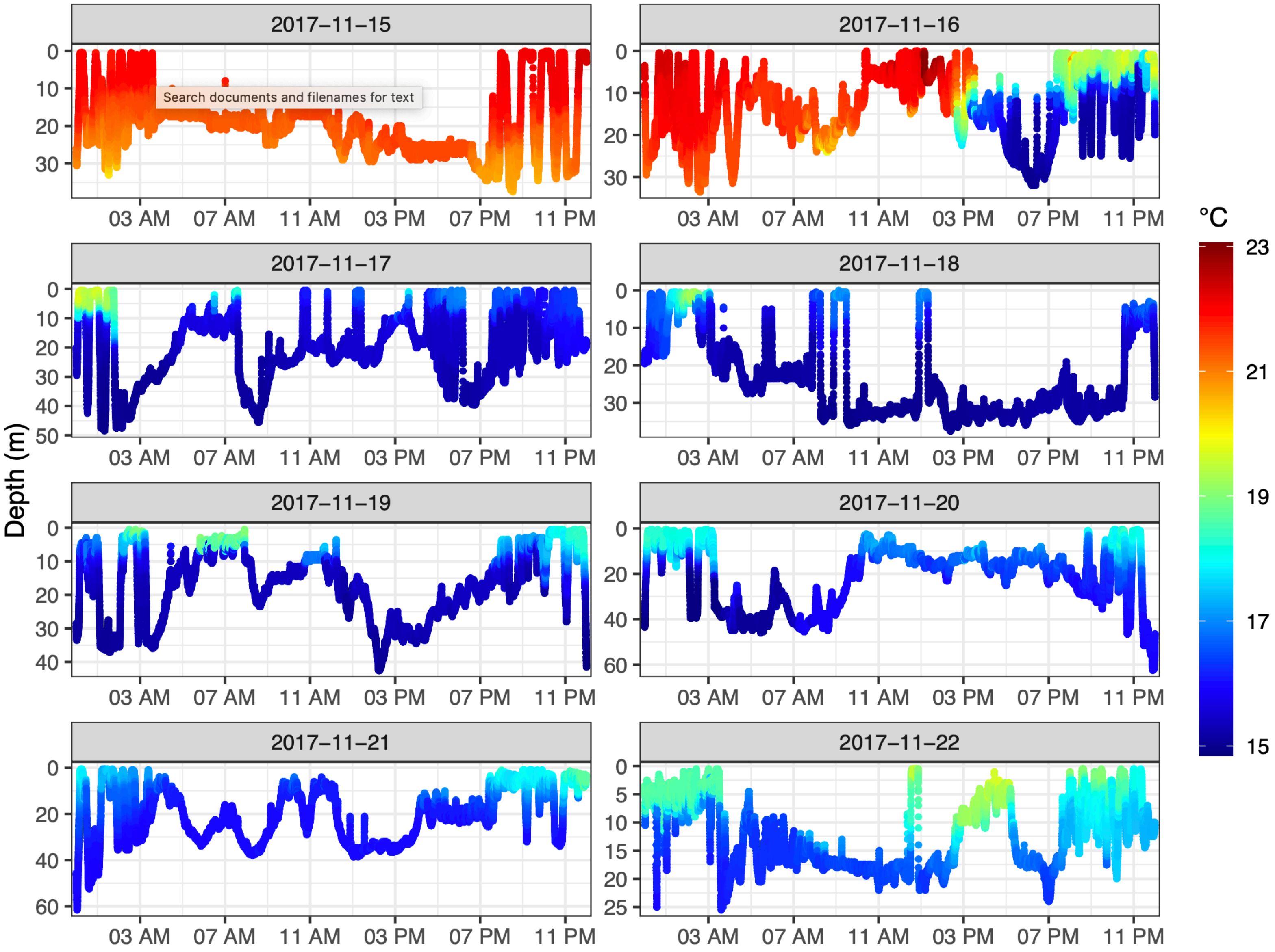
2017–11–15
2017–11–16
2017–11–17
2017–11–18
2017–11–19
2017–11–20
2017–11–21
2017–11–22
Depth (m)
03 AM
07 AM
11 AM
03 PM
07 PM
11 PM
°C
23
21
19
17
15
Search documents and filenames for text

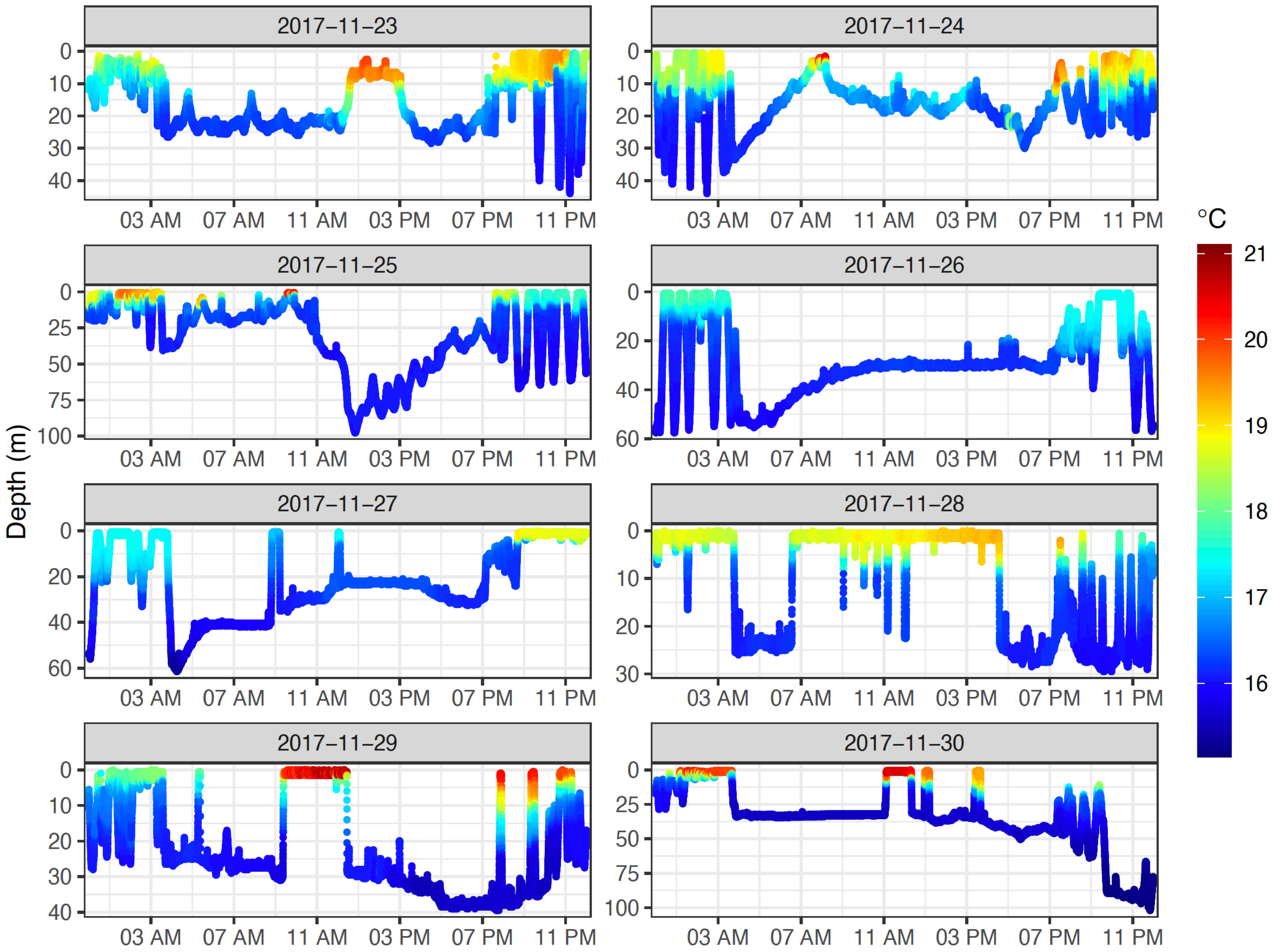

2017−11−23
2017−11−24
2017−11−25
2017−11−26
2017−11−27
2017−11−28
2017−11−29
2017−11−30
Depth (m)
03 AM 07 AM 11 AM 03 PM 07 PM 11 PM
°C
21
20
19
18
17
16

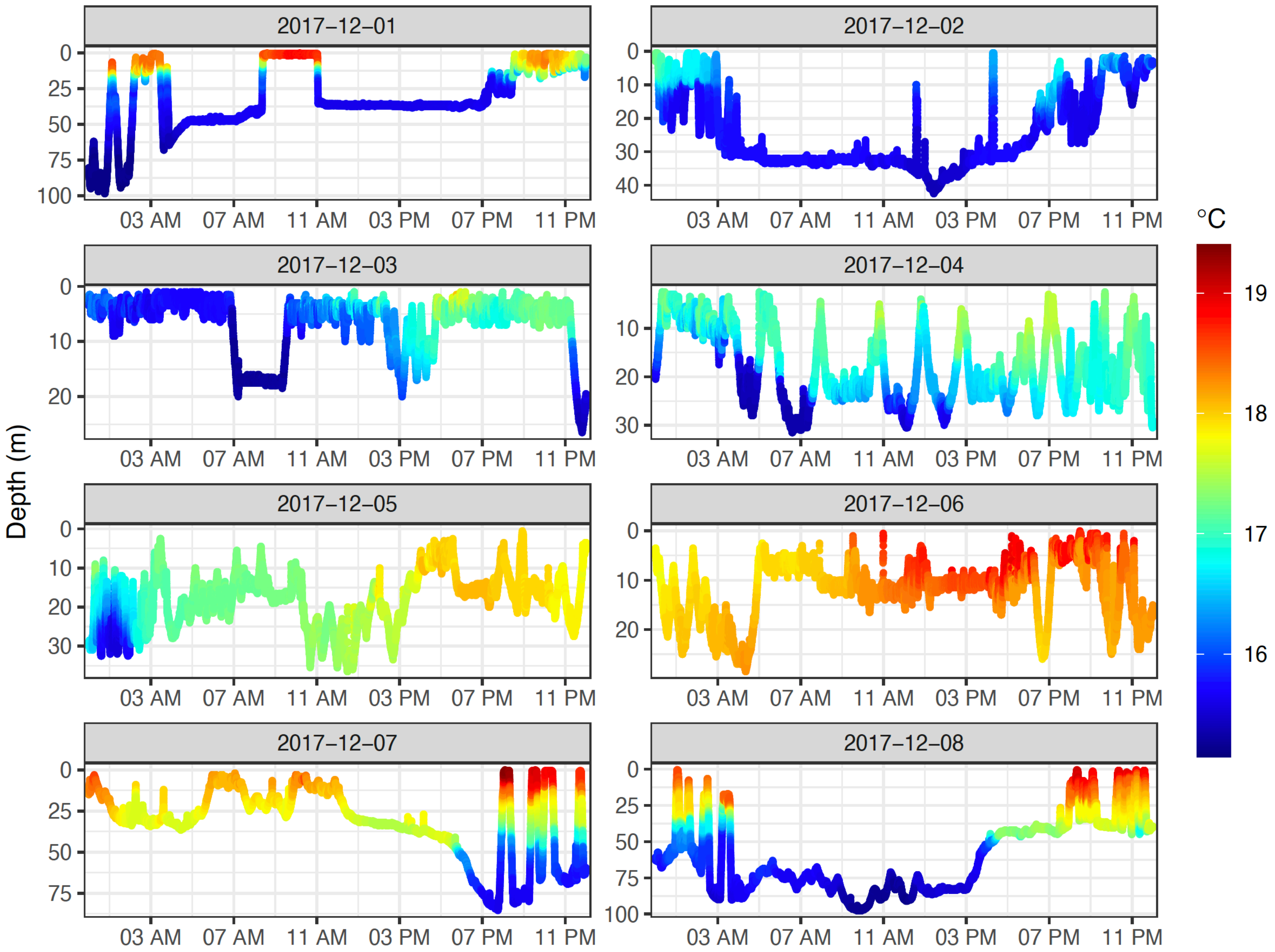
2017–12–01
2017–12–02
2017–12–03
2017–12–04
2017–12–05
2017–12–06
2017–12–07
2017–12–08
Depth (m)
°C
19
18
17
16
03 AM
07 AM
11 AM
03 PM
07 PM
11 PM

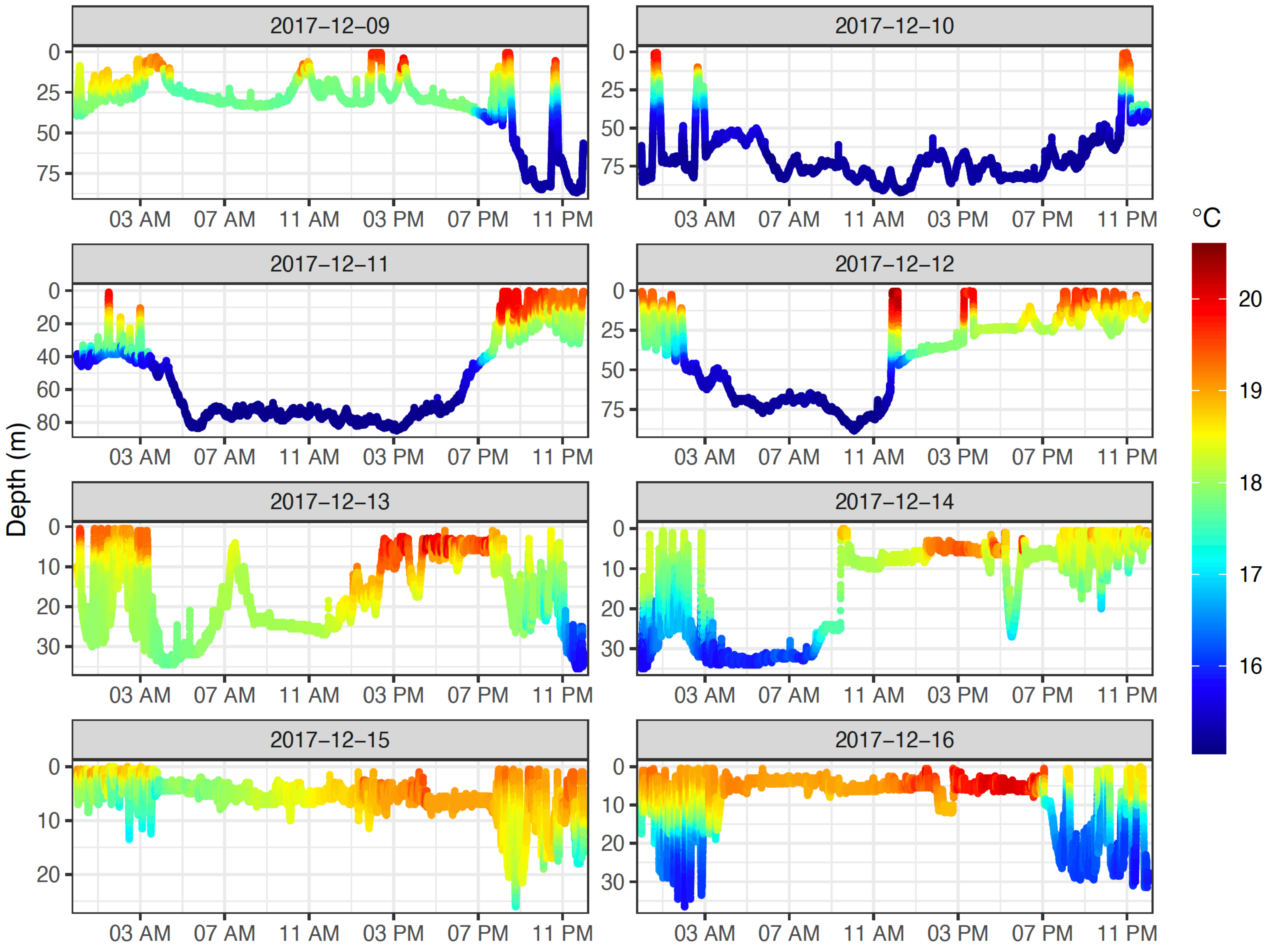

2017−12−09
2017−12−10
2017−12−11
2017−12−12
2017−12−13
2017−12−14
2017−12−15
2017−12−16
Depth (m)
03 AM
07 AM
11 AM
03 PM
07 PM
11 PM
°C
20
19
18
17
16

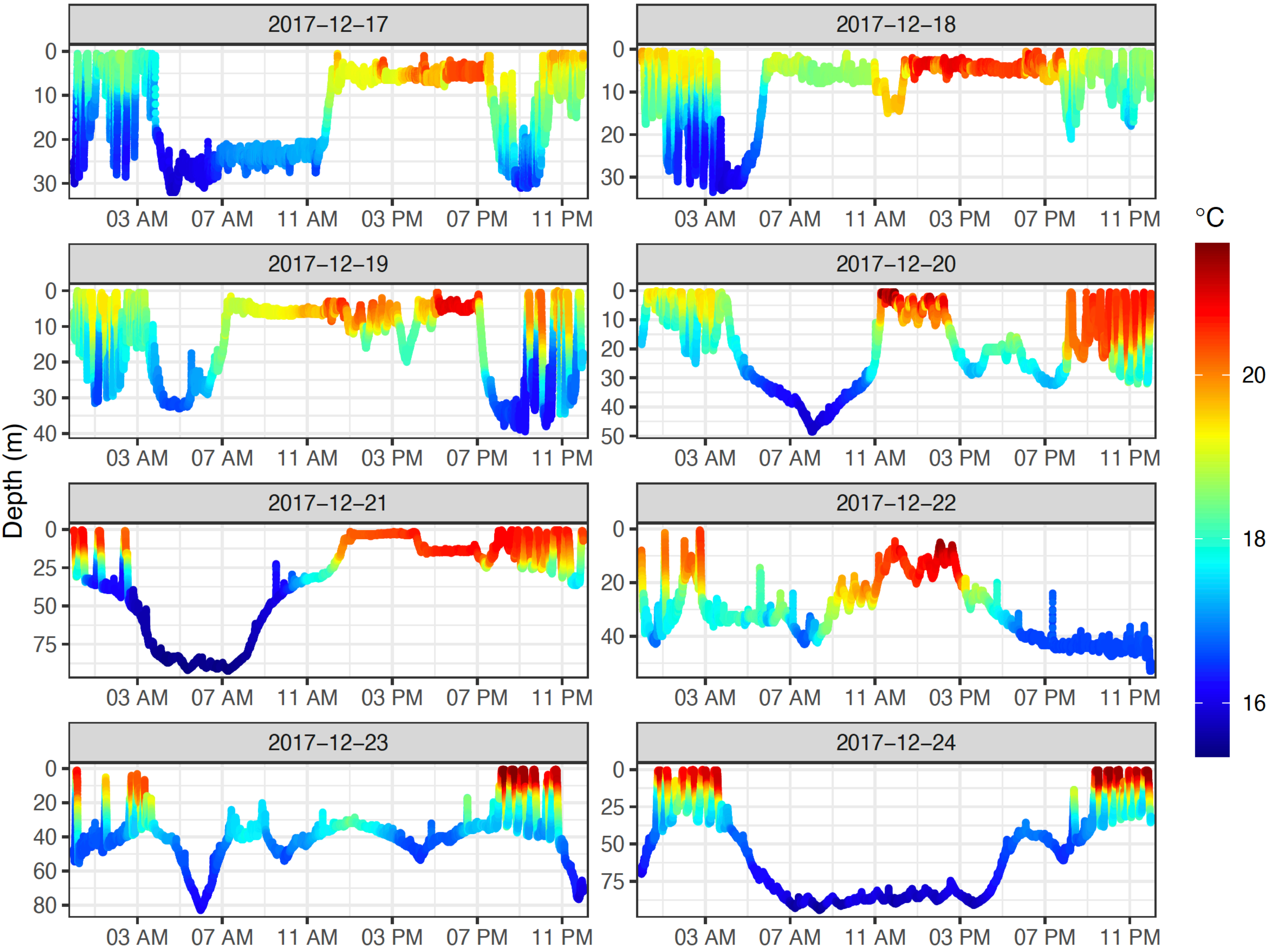

2017−12−17
2017−12−18
2017−12−19
2017−12−20
2017−12−21
2017−12−22
2017−12−23
2017−12−24
Depth (m)
03 AM
07 AM
11 AM
03 PM
07 PM
11 PM
°C
20
18
16

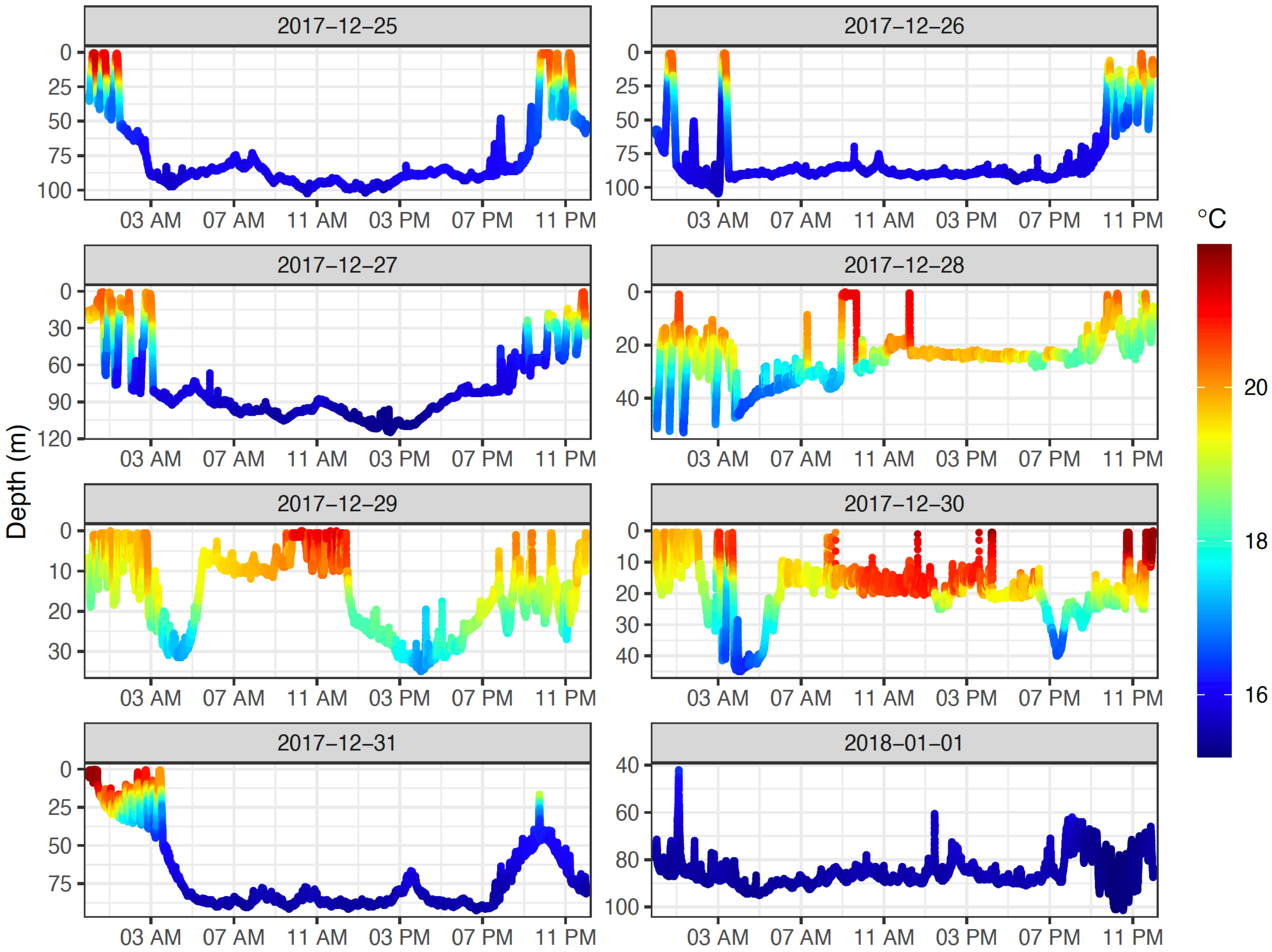

2017–12–25
2017–12–26
2017–12–27
2017–12–28
2017–12–29
2017–12–30
2017–12–31
2018–01–01
Depth (m)
°C
20
18
16
03 AM
07 AM
11 AM
03 PM
07 PM
11 PM

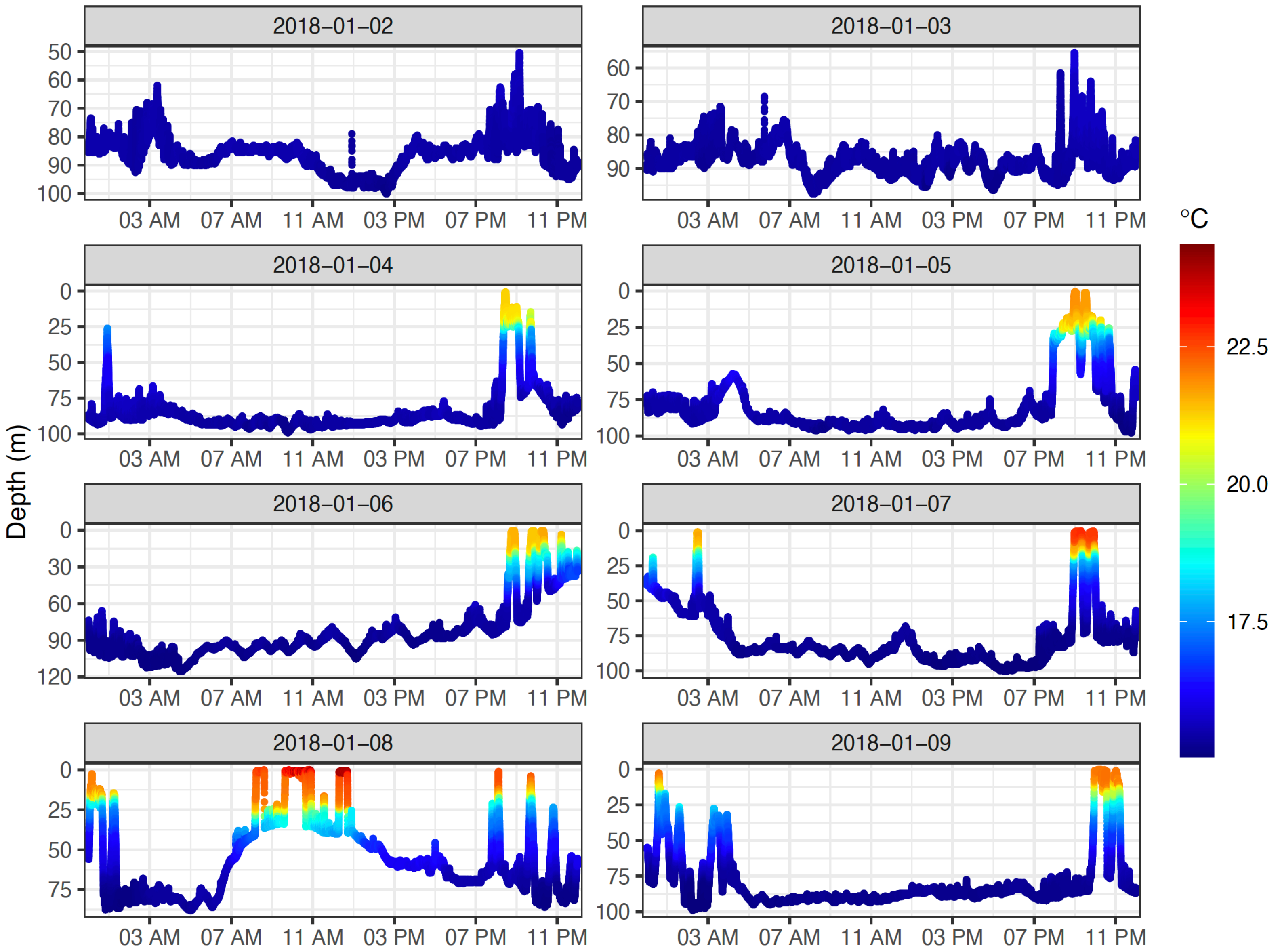

2018–01–02
2018–01–03
2018–01–04
2018–01–05
2018–01–06
2018–01–07
2018–01–08
2018–01–09
Depth (m)
°C
22.5
20.0
17.5
03 AM
07 AM
11 AM
03 PM
07 PM
11 PM

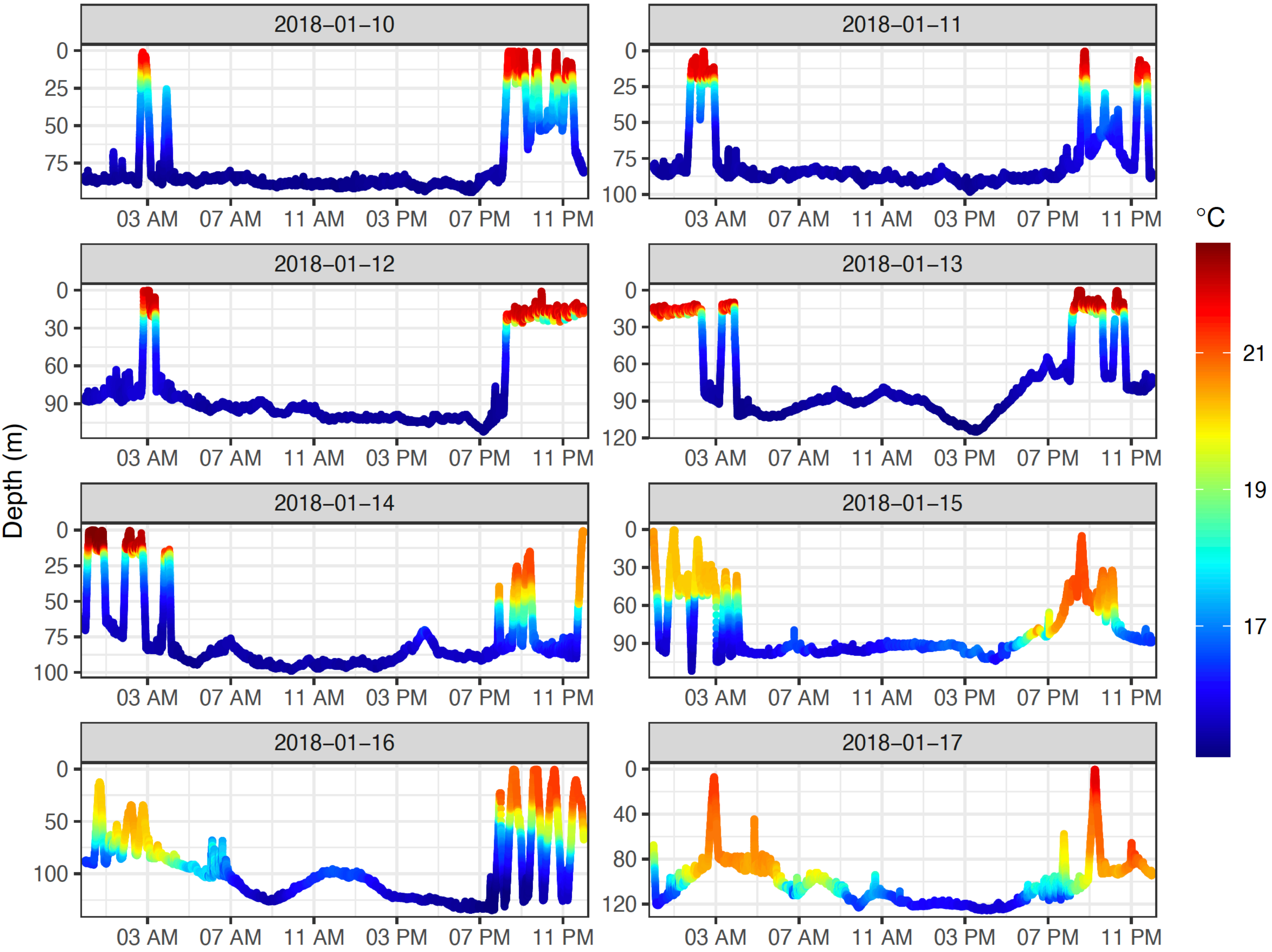

2018–01–10
2018–01–11
2018–01–12
2018–01–13
2018–01–14
2018–01–15
2018–01–16
2018–01–17
Depth (m)
03 AM
07 AM
11 AM
03 PM
07 PM
11 PM
°C
21
19
17

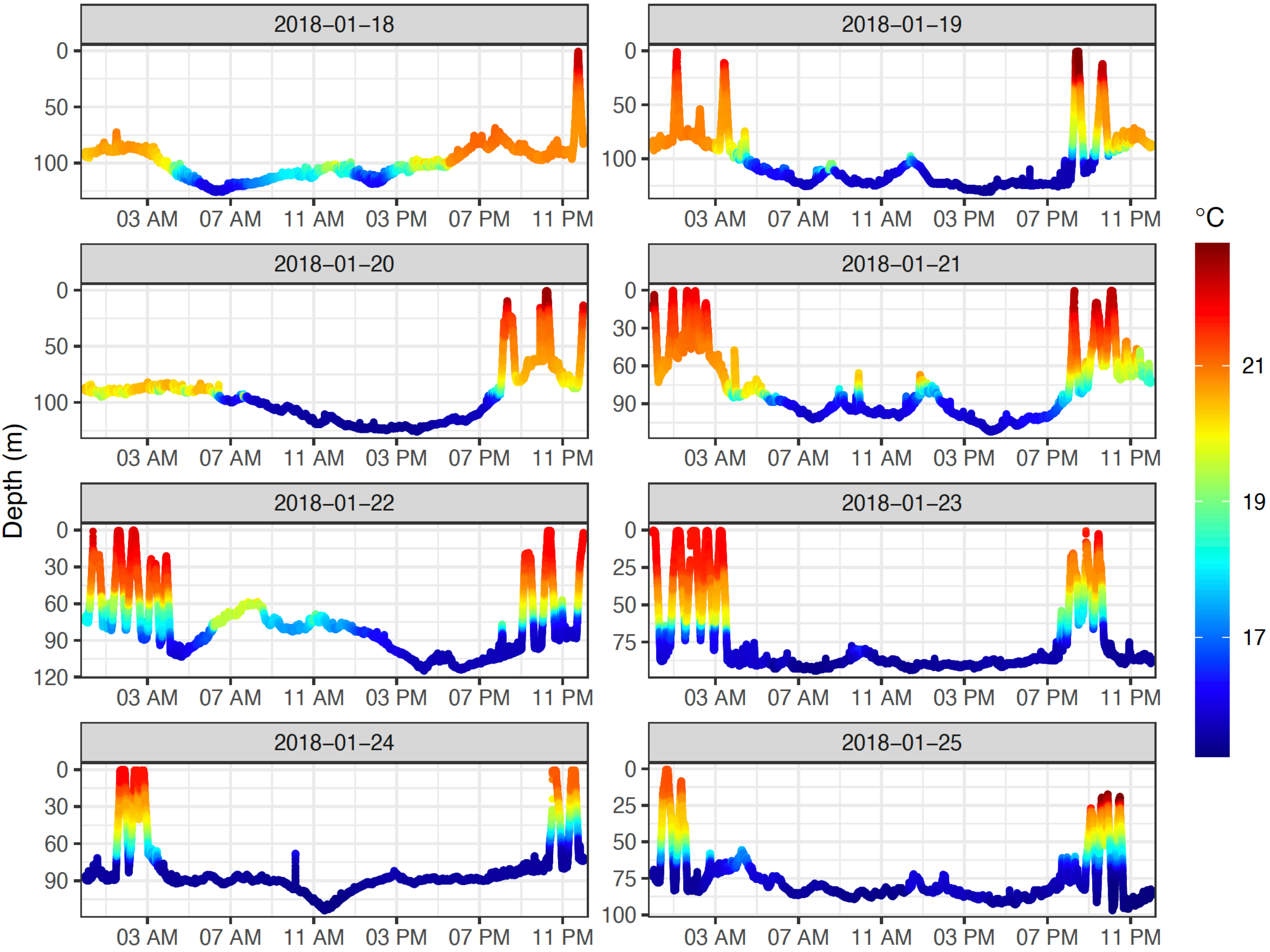

2018−01−18
2018−01−19
2018−01−20
2018−01−21
2018−01−22
2018−01−23
2018−01−24
2018−01−25
Depth (m)
03 AM
07 AM
11 AM
03 PM
07 PM
11 PM
°C
21
19
17

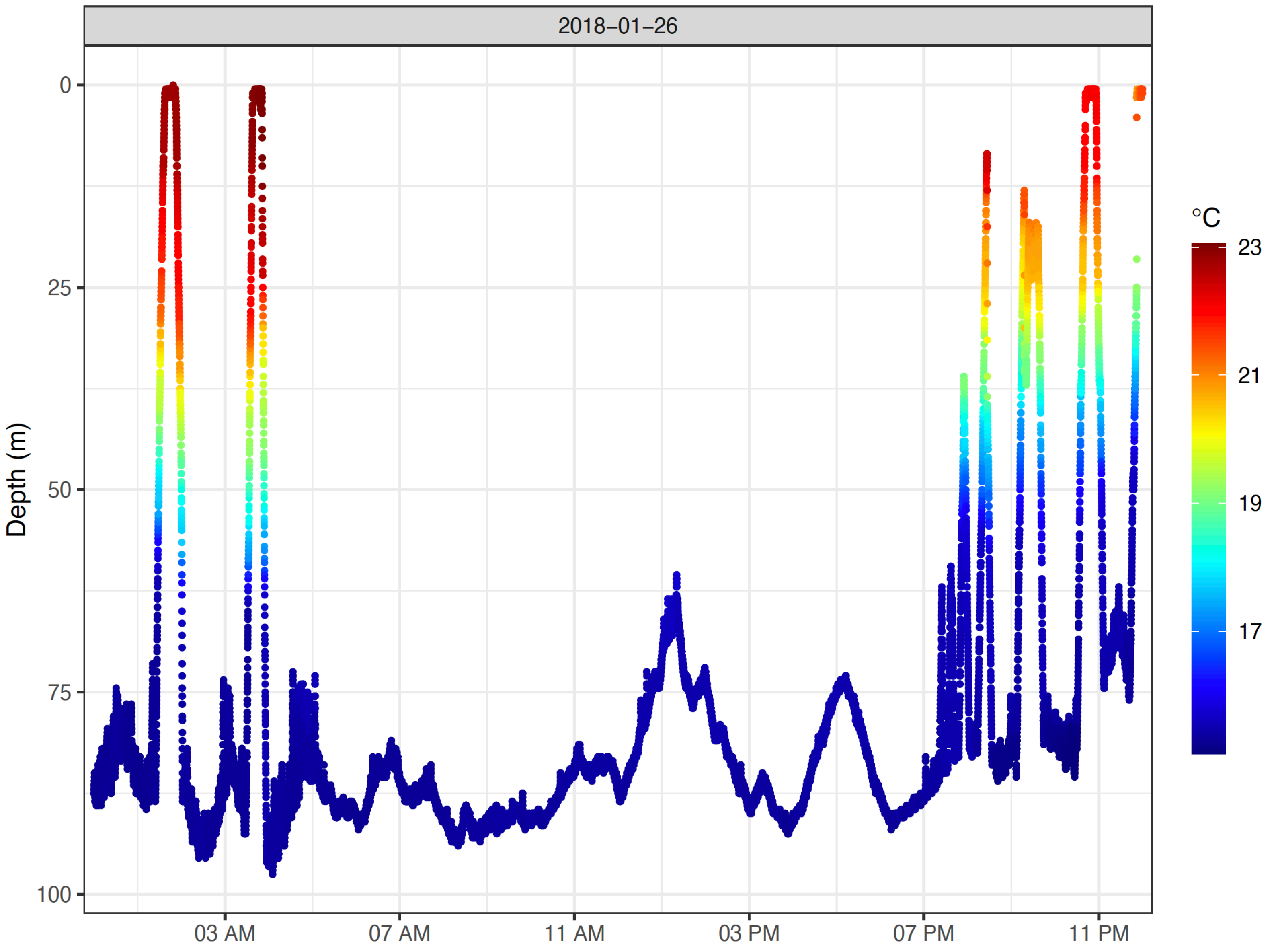
2018–01–26
Depth (m)
0
25
50
75
100
03 AM
07 AM
11 AM
03 PM
07 PM
11 PM
°C
23
21
19
17

Shark 356

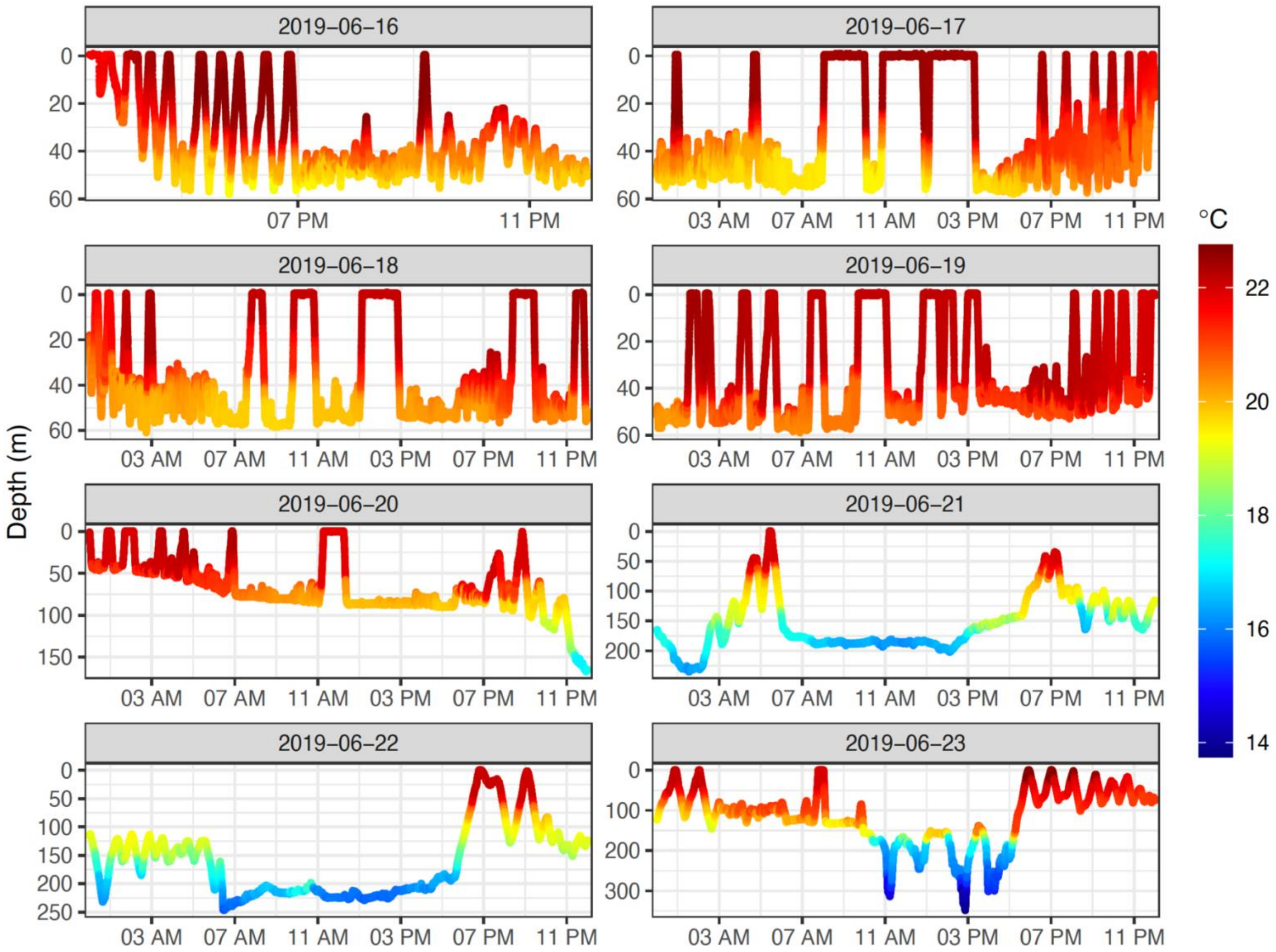

Depth (m)
°C
22
20
18
16
14
2019–06–16
0
20
40
60
07 PM
11 PM
2019–06–17
03 AM
07 AM
11 AM
03 PM
07 PM
11 PM
2019–06–18
2019–06–19
2019–06–20
0
50
100
150
2019–06–21
200
2019–06–22
250
2019–06–23
300

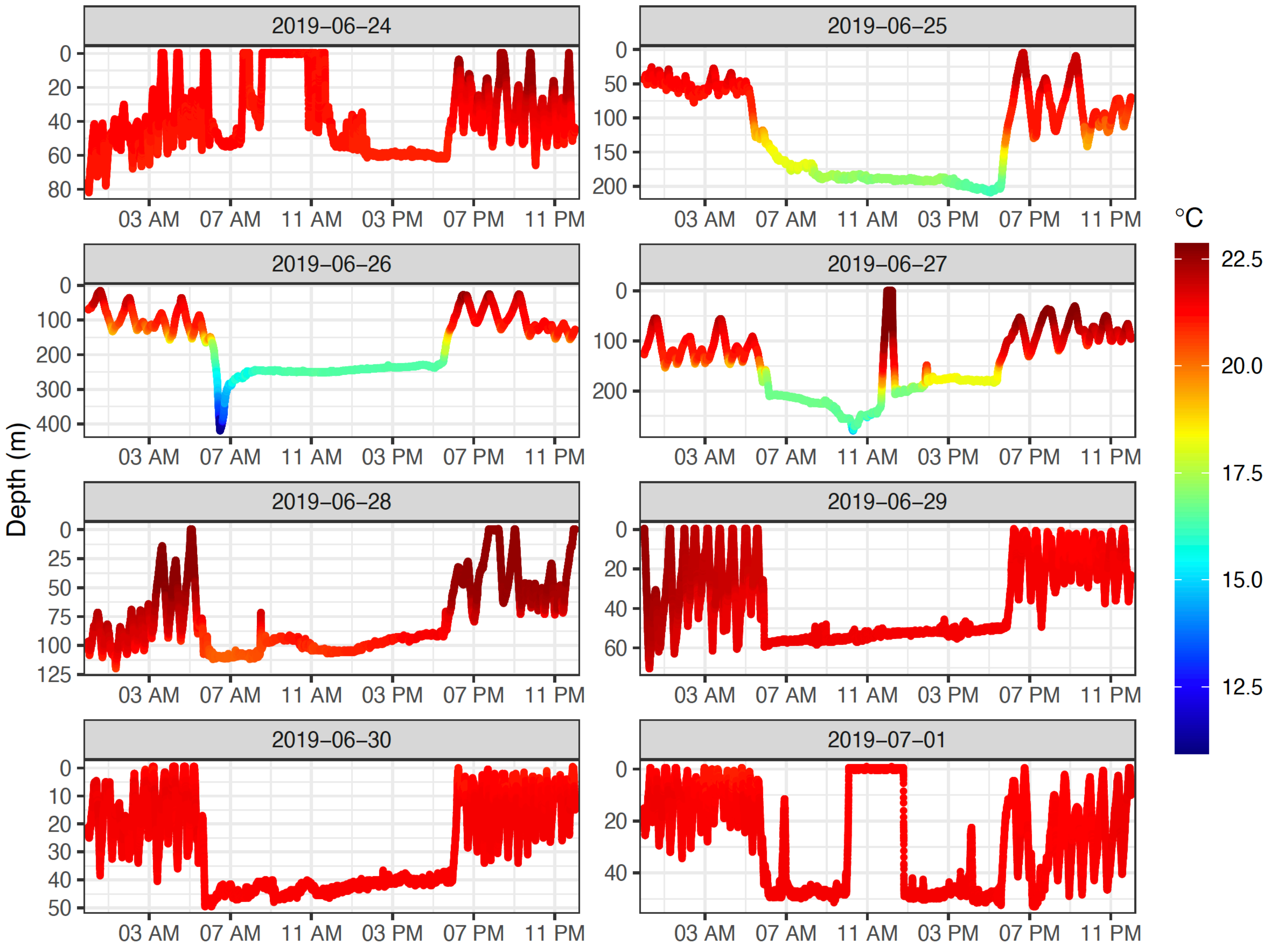
2019–06–24
2019–06–25
2019–06–26
2019–06–27
2019–06–28
2019–06–29
2019–06–30
2019–07–01
Depth (m)
03 AM
07 AM
11 AM
03 PM
07 PM
11 PM
°C
22.5
20.0
17.5
15.0
12.5

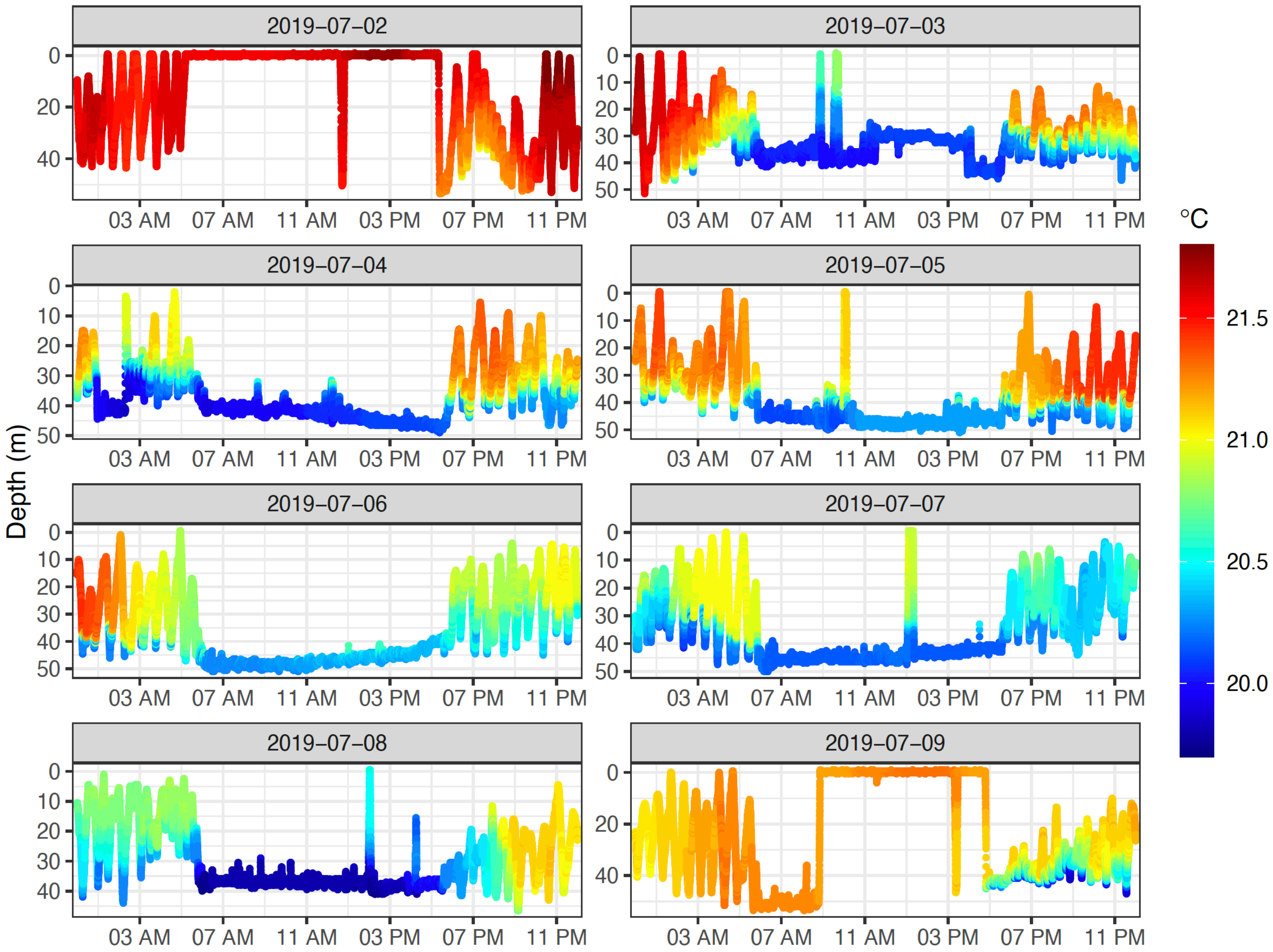

2019–07–02
2019–07–03
2019–07–04
2019–07–05
2019–07–06
2019–07–07
2019–07–08
2019–07–09
Depth (m)
03 AM
07 AM
11 AM
03 PM
07 PM
11 PM
°C
21.5
21.0
20.5
20.0

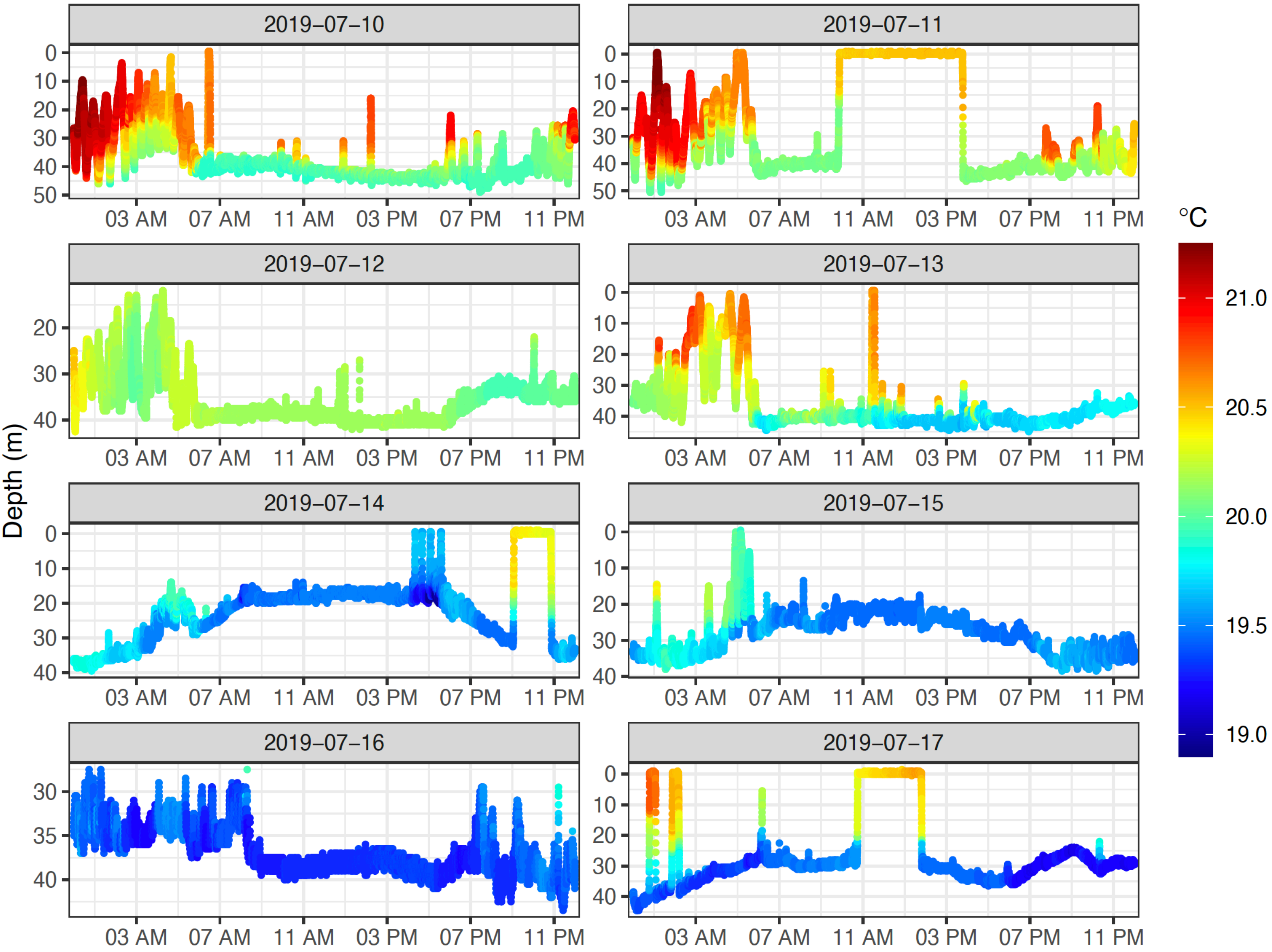

2019–07–10
2019–07–11
2019–07–12
2019–07–13
2019–07–14
2019–07–15
2019–07–16
2019–07–17
Depth (m)
03 AM
07 AM
11 AM
03 PM
07 PM
11 PM
°C
21.0
20.5
20.0
19.5
19.0

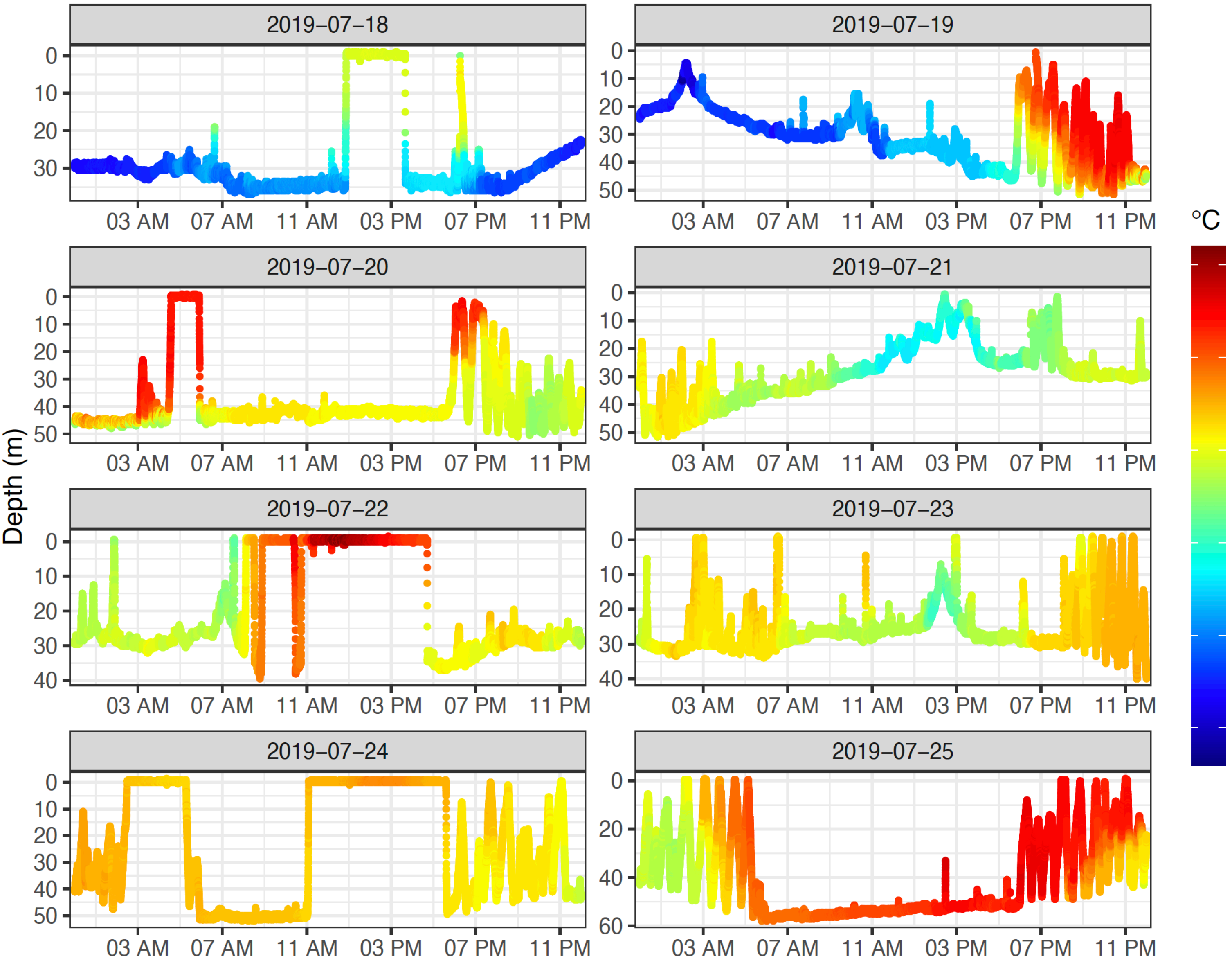

2019–07–18
2019–07–19
2019–07–20
2019–07–21
2019–07–22
2019–07–23
2019–07–24
2019–07–25
03 AM
07 AM
11 AM
03 PM
07 PM
11 PM
Depth (m)
°C
21.5
21.0
20.5
20.0
19.5
19.0

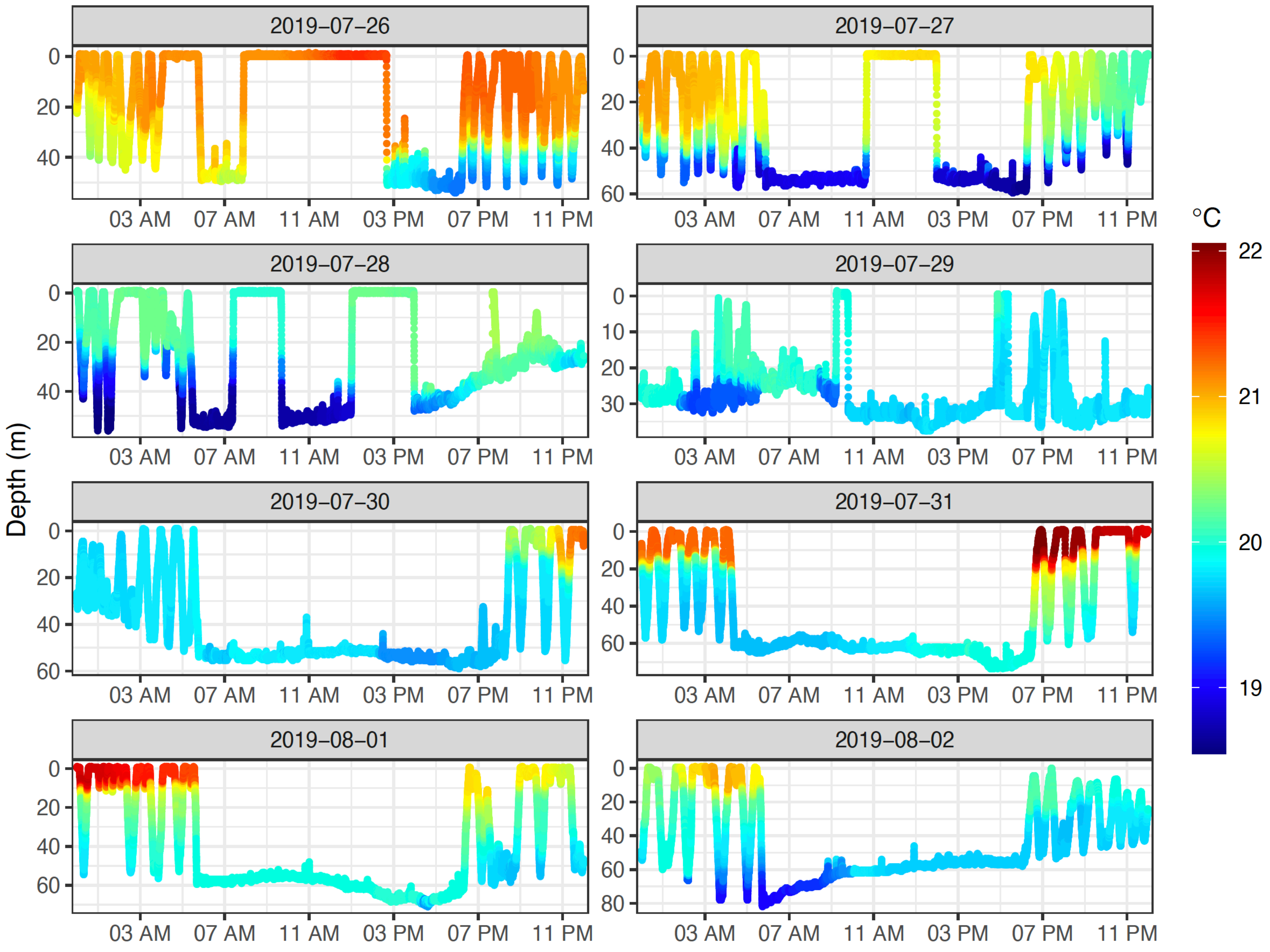

2019–07–26
2019–07–27
2019–07–28
2019–07–29
2019–07–30
2019–07–31
2019–08–01
2019–08–02
03 AM
07 AM
11 AM
03 PM
07 PM
11 PM
Depth (m)
°C
22
21
20
19

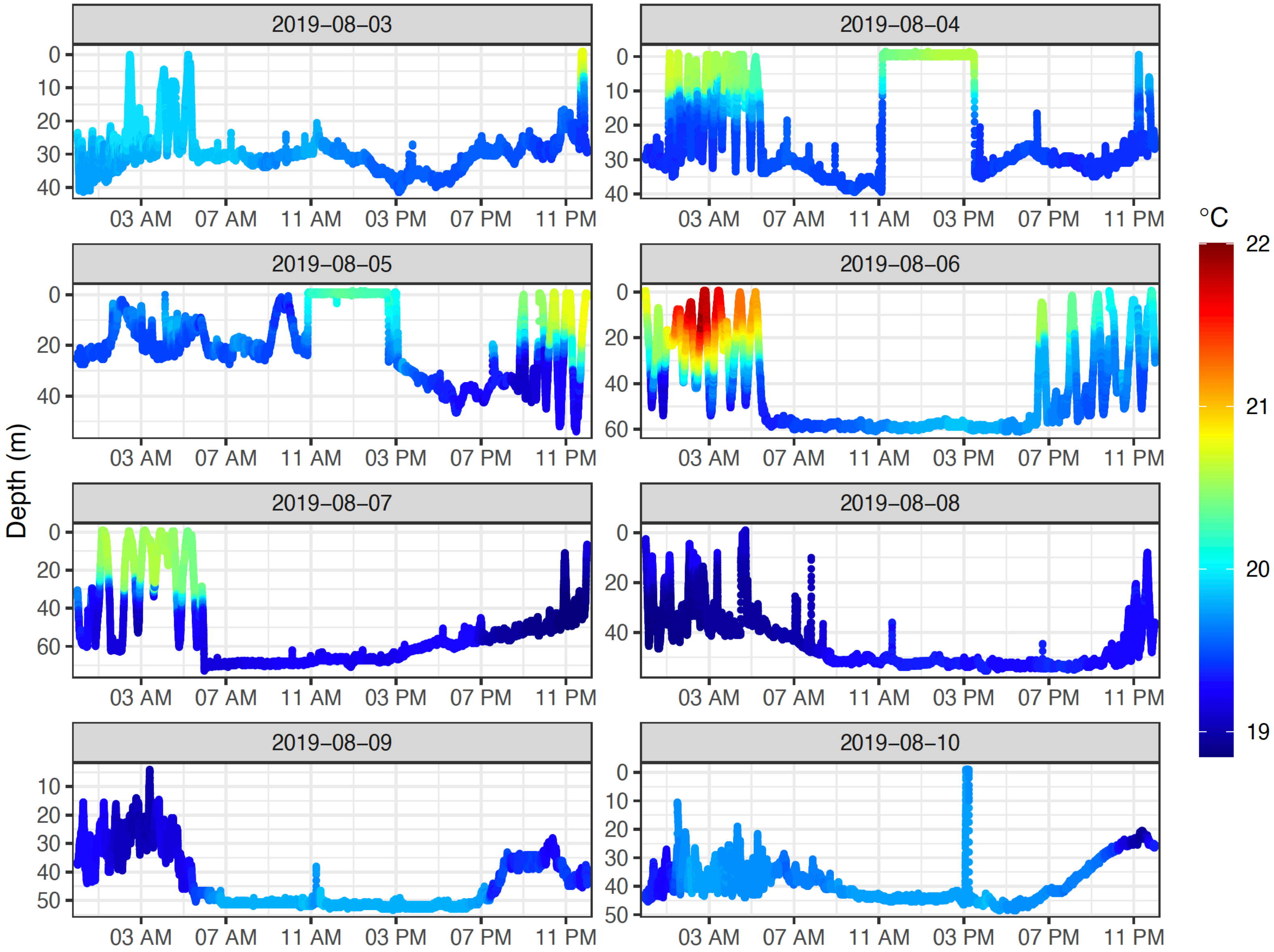
2019–08–03
2019–08–04
2019–08–05
2019–08–06
2019–08–07
2019–08–08
2019–08–09
2019–08–10
Depth (m)
03 AM
07 AM
11 AM
03 PM
07 PM
11 PM
°C
22
21
20
19

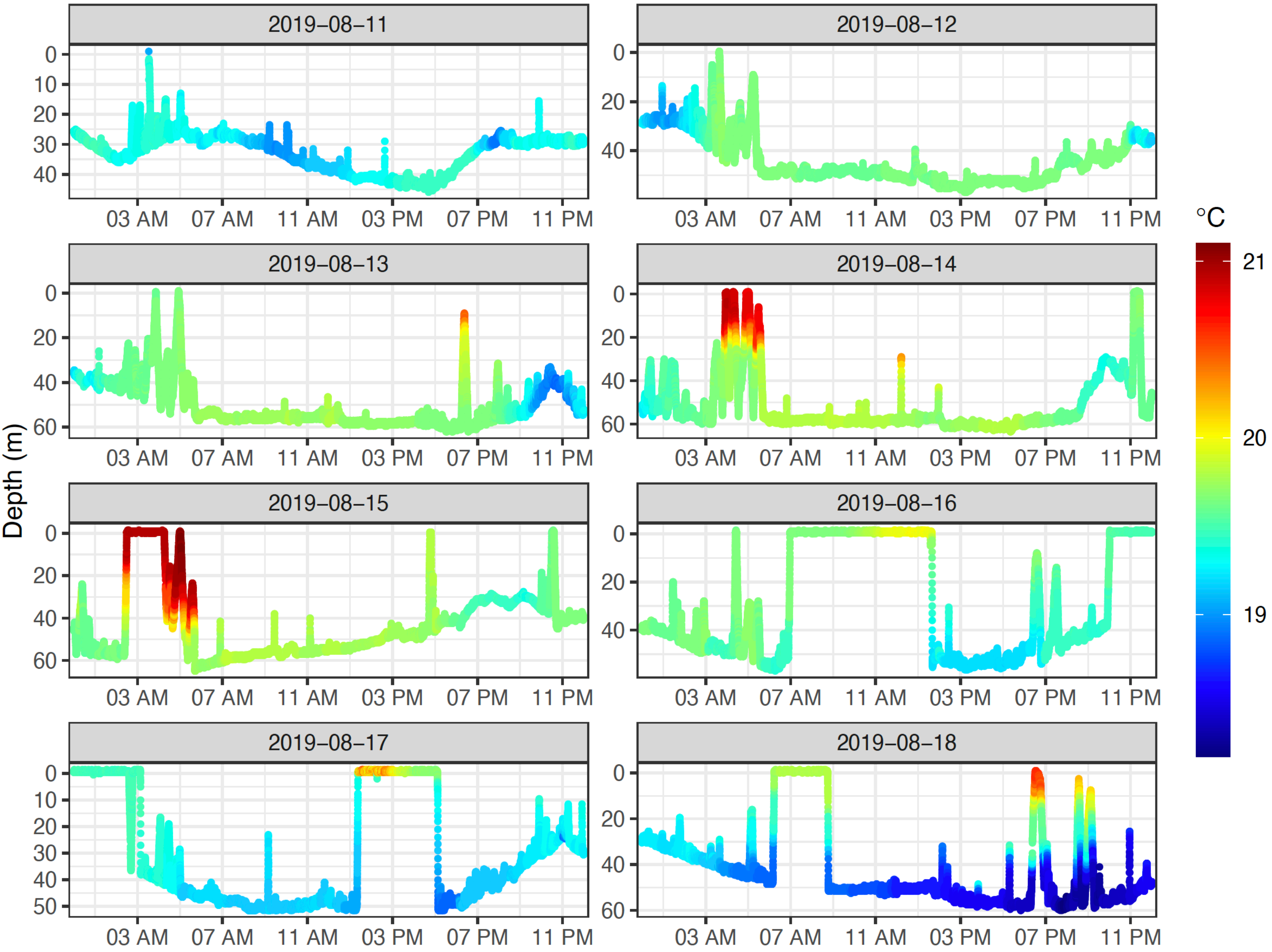
2019−08−11
2019−08−12
2019−08−13
2019−08−14
2019−08−15
2019−08−16
2019−08−17
2019−08−18
Depth (m)
03 AM
07 AM
11 AM
03 PM
07 PM
11 PM
°C
21
20
19

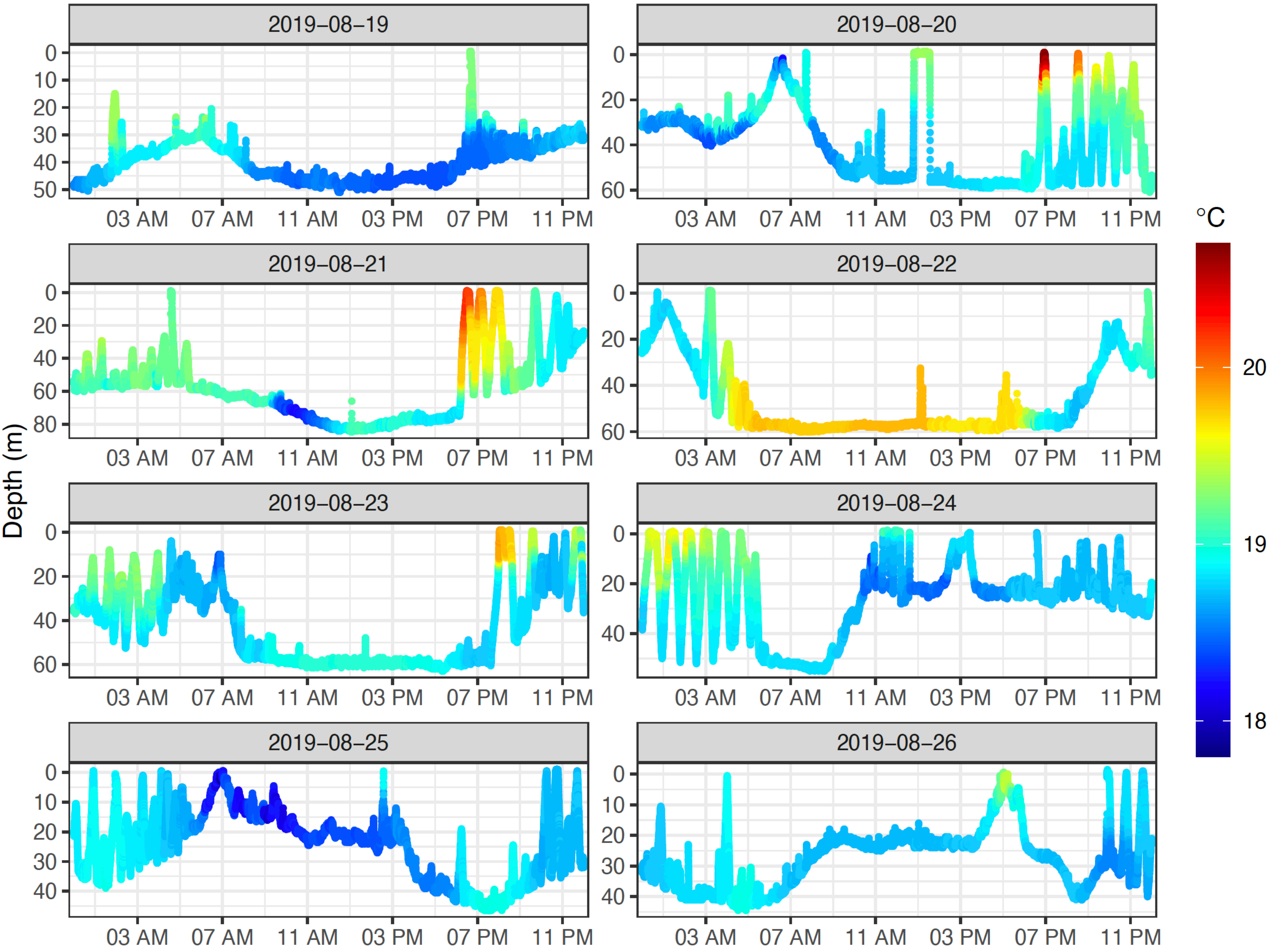
2019−08−19
2019−08−20
2019−08−21
2019−08−22
2019−08−23
2019−08−24
2019−08−25
2019−08−26
Depth (m)
03 AM
07 AM
11 AM
03 PM
07 PM
11 PM
°C
20
19
18

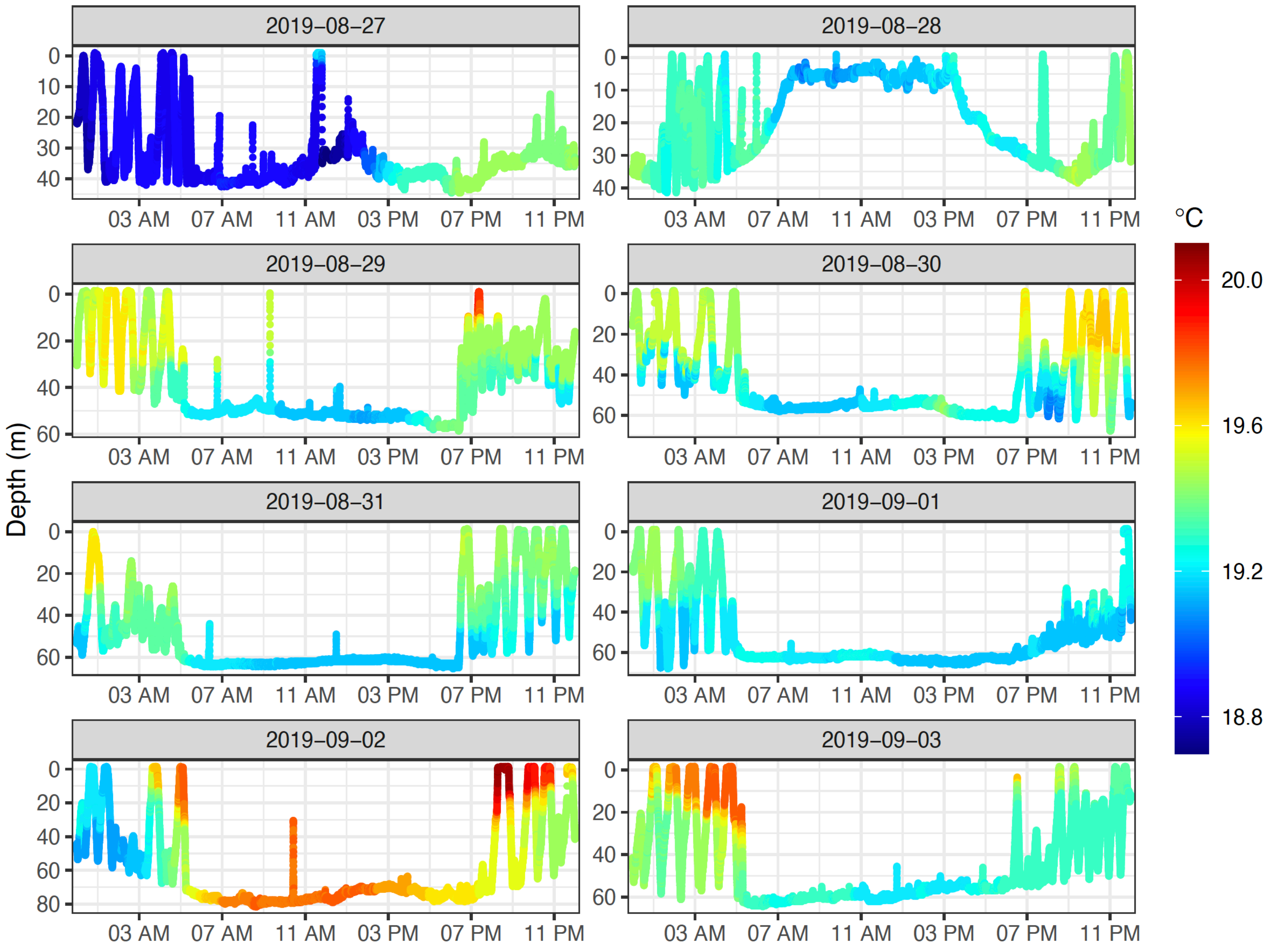
2019−08−27
2019−08−28
2019−08−29
2019−08−30
2019−08−31
2019−09−01
2019−09−02
2019−09−03
Depth (m)
03 AM
07 AM
11 AM
03 PM
07 PM
11 PM
°C
20.0
19.6
19.2
18.8

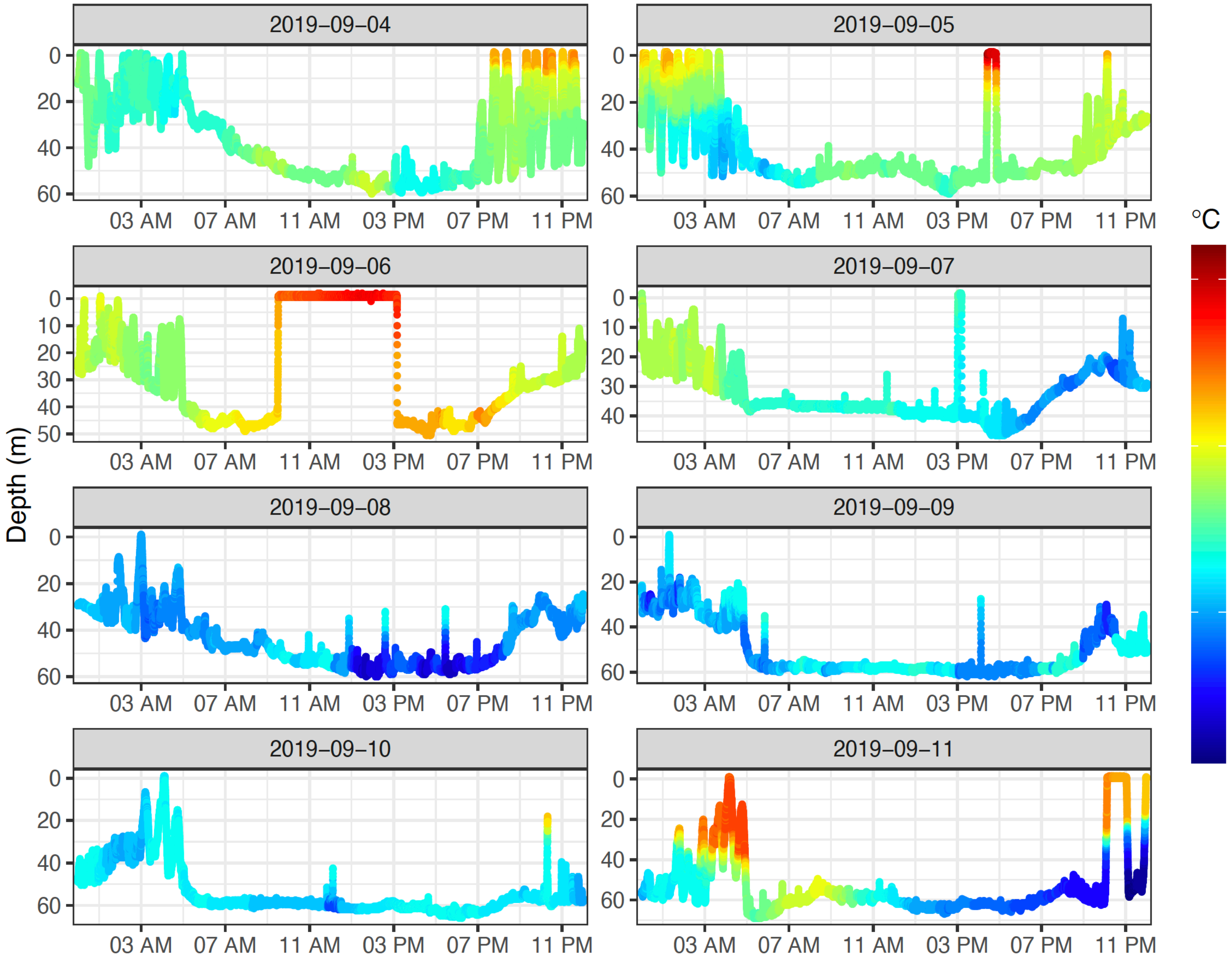
2019–09–04
2019–09–05
2019–09–06
2019–09–07
2019–09–08
2019–09–09
2019–09–10
2019–09–11
Depth (m)
°C
20.0
19.5
19.0
03 AM
07 AM
11 AM
03 PM
07 PM
11 PM

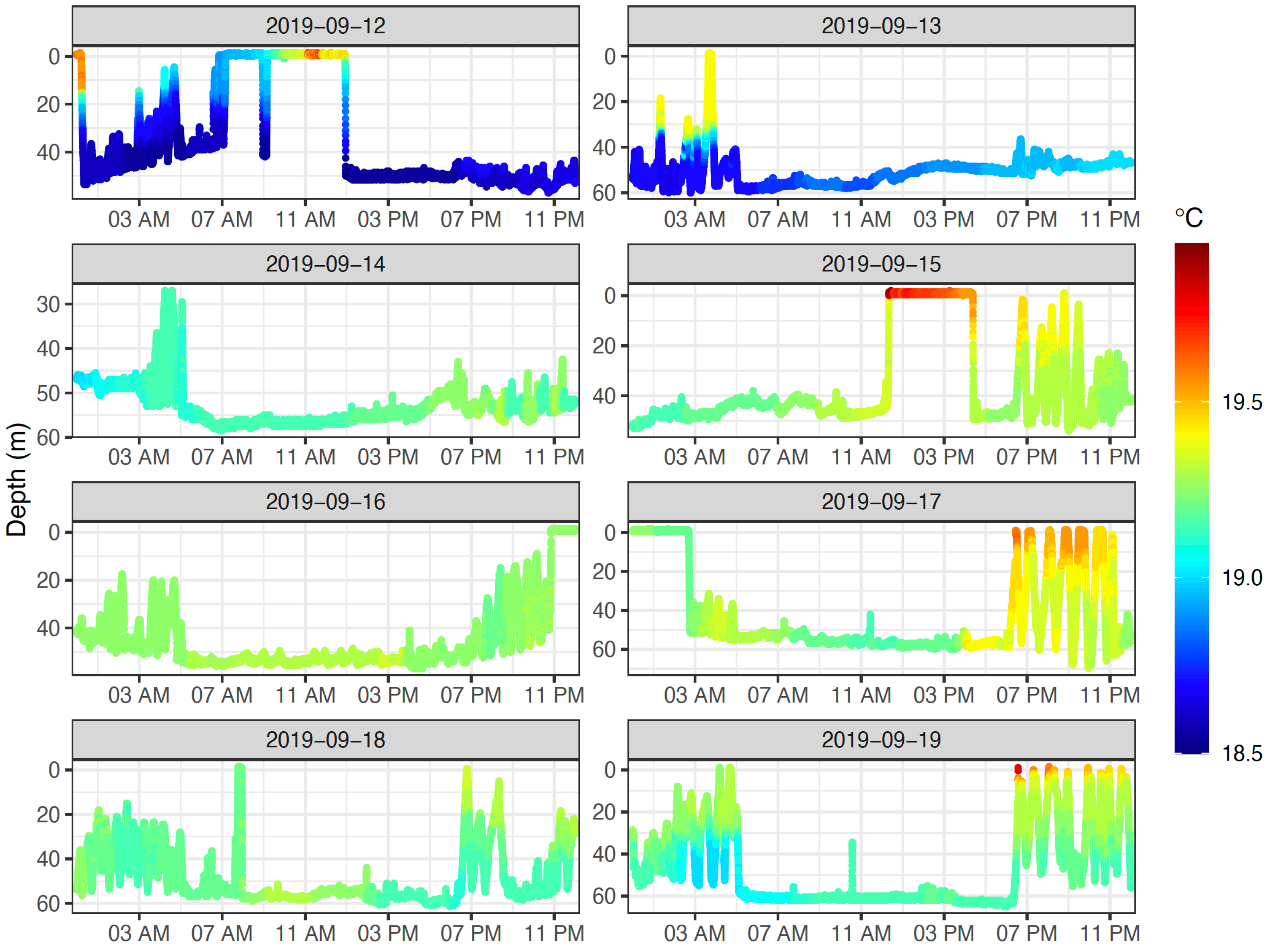

2019–09–12
2019–09–13
2019–09–14
2019–09–15
2019–09–16
2019–09–17
2019–09–18
2019–09–19
Depth (m)
03 AM
07 AM
11 AM
03 PM
07 PM
11 PM
°C
19.5
19.0
18.5

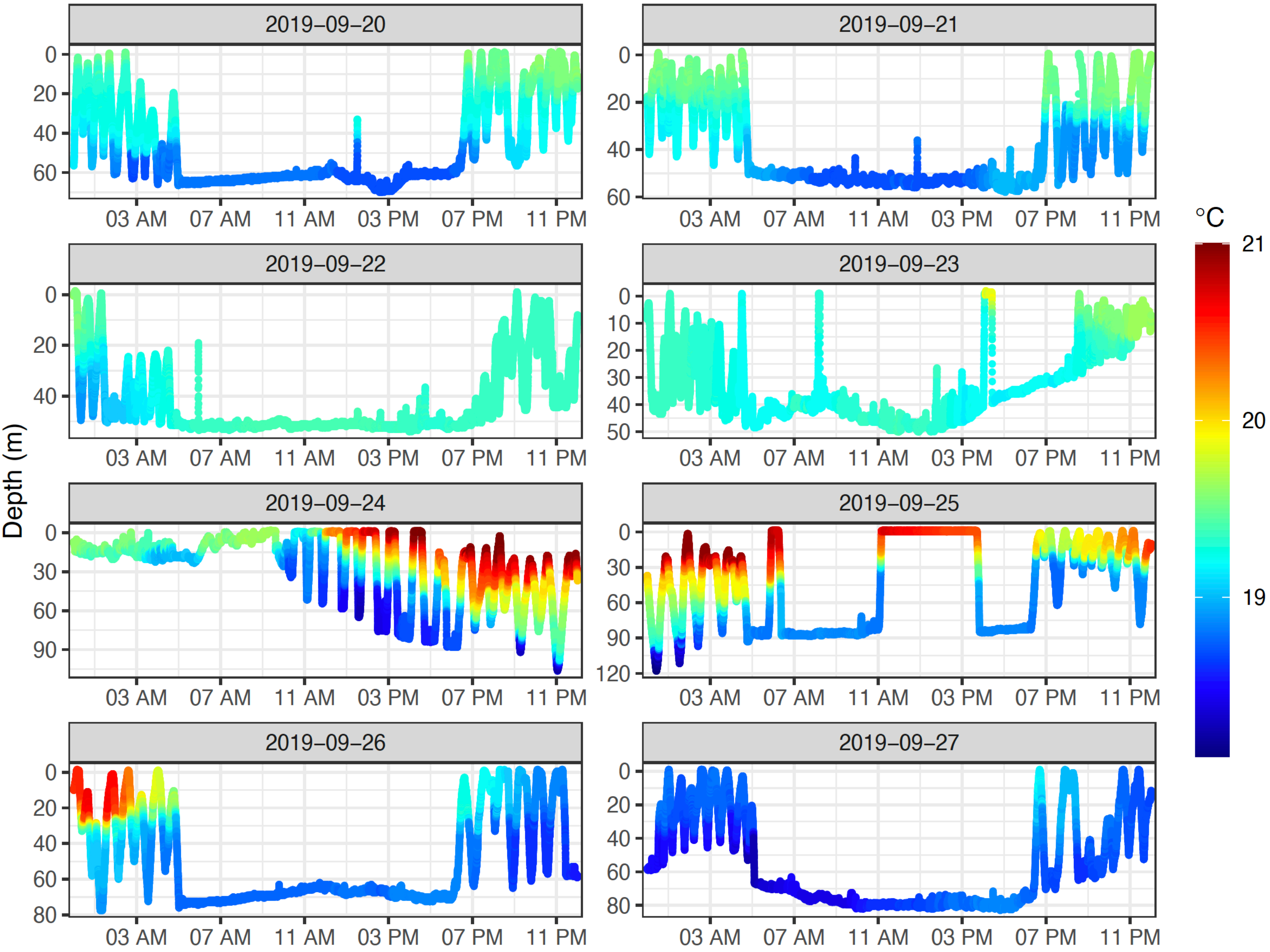
2019−09−20
2019−09−21
2019−09−22
2019−09−23
2019−09−24
2019−09−25
2019−09−26
2019−09−27
Depth (m)
03 AM
07 AM
11 AM
03 PM
07 PM
11 PM
°C
21
20
19

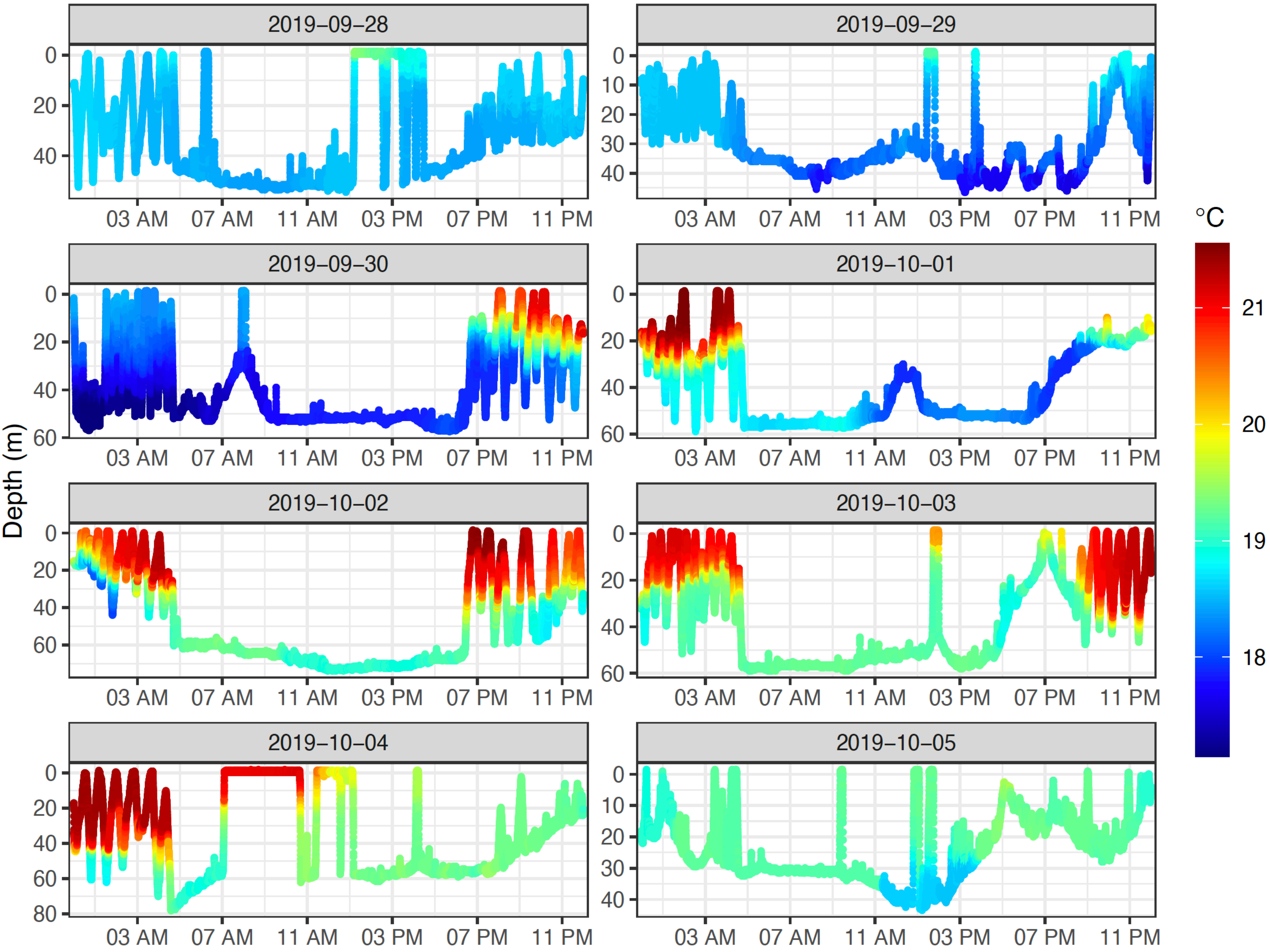

2019–09–28
2019–09–29
2019–09–30
2019–10–01
2019–10–02
2019–10–03
2019–10–04
2019–10–05
Depth (m)
03 AM
07 AM
11 AM
03 PM
07 PM
11 PM
°C
21
20
19
18

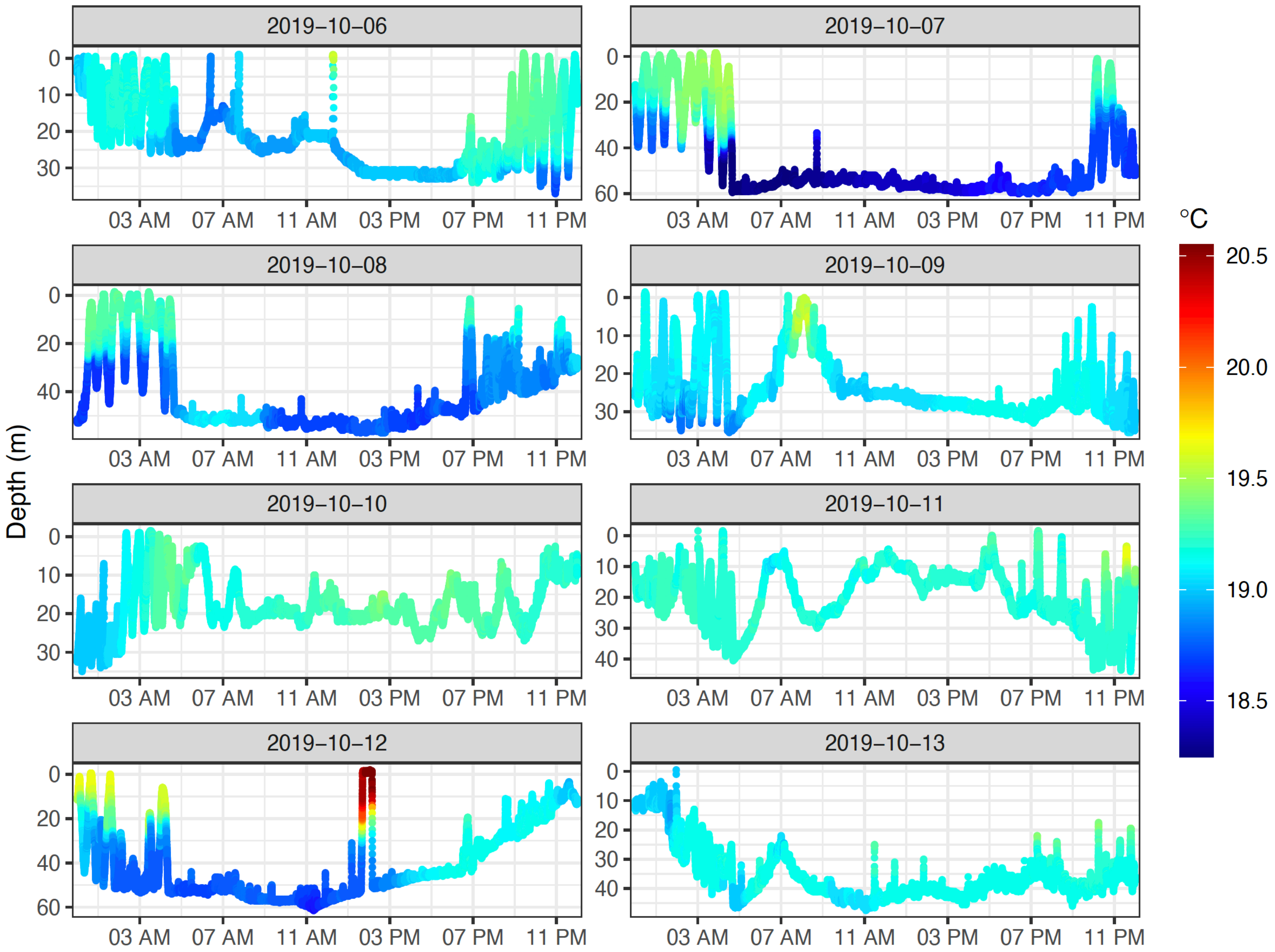

2019–10–06
2019–10–07
2019–10–08
2019–10–09
2019–10–10
2019–10–11
2019–10–12
2019–10–13
Depth (m)
03 AM
07 AM
11 AM
03 PM
07 PM
11 PM
°C
20.5
20.0
19.5
19.0
18.5

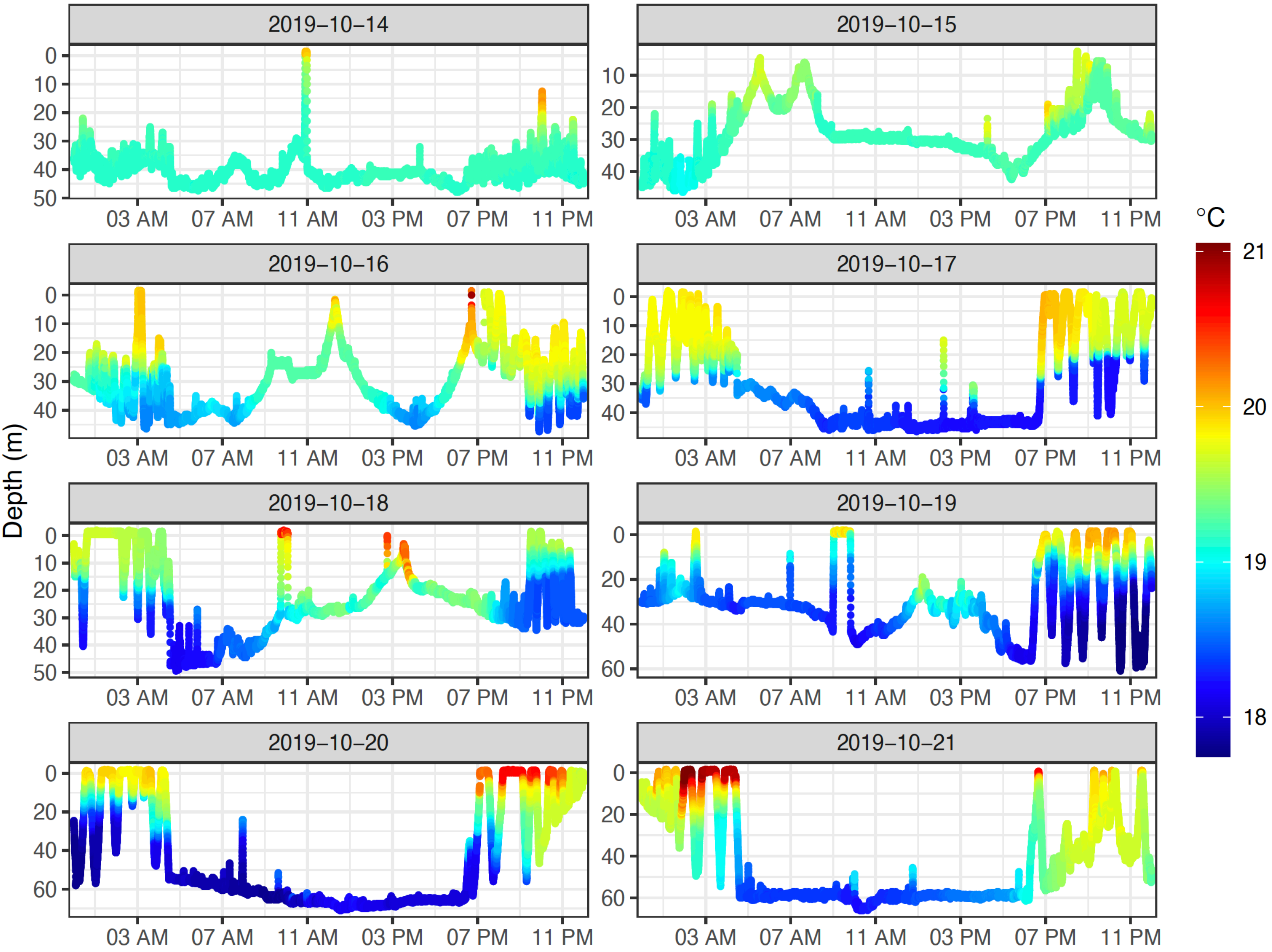

2019−10−14
2019−10−15
2019−10−16
2019−10−17
2019−10−18
2019−10−19
2019−10−20
2019−10−21
Depth (m)
03 AM
07 AM
11 AM
03 PM
07 PM
11 PM
°C
21
20
19
18

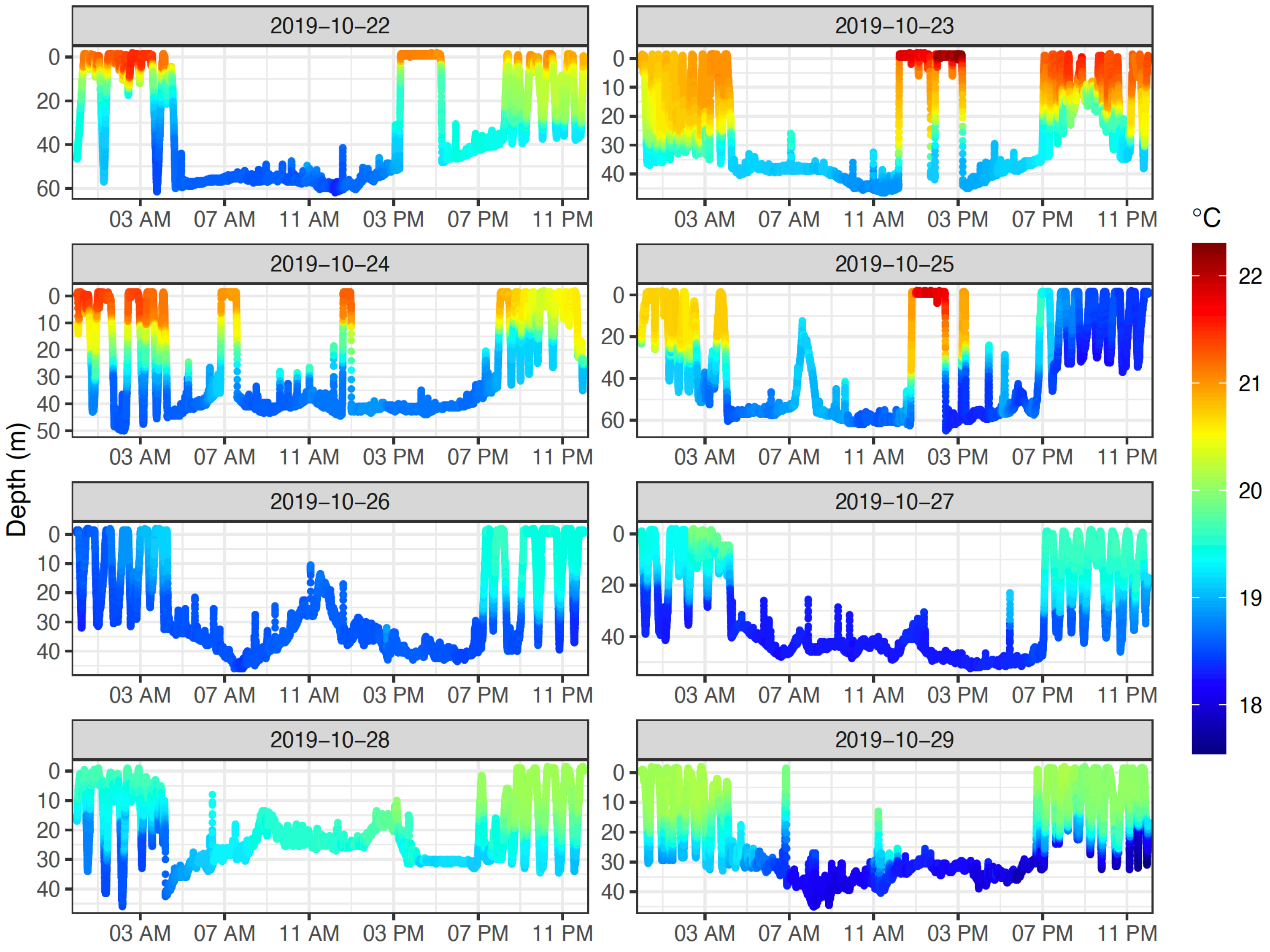
2019–10–22
2019–10–23
2019–10–24
2019–10–25
2019–10–26
2019–10–27
2019–10–28
2019–10–29
Depth (m)
°C
22
21
20
19
18
03 AM
07 AM
11 AM
03 PM
07 PM
11 PM

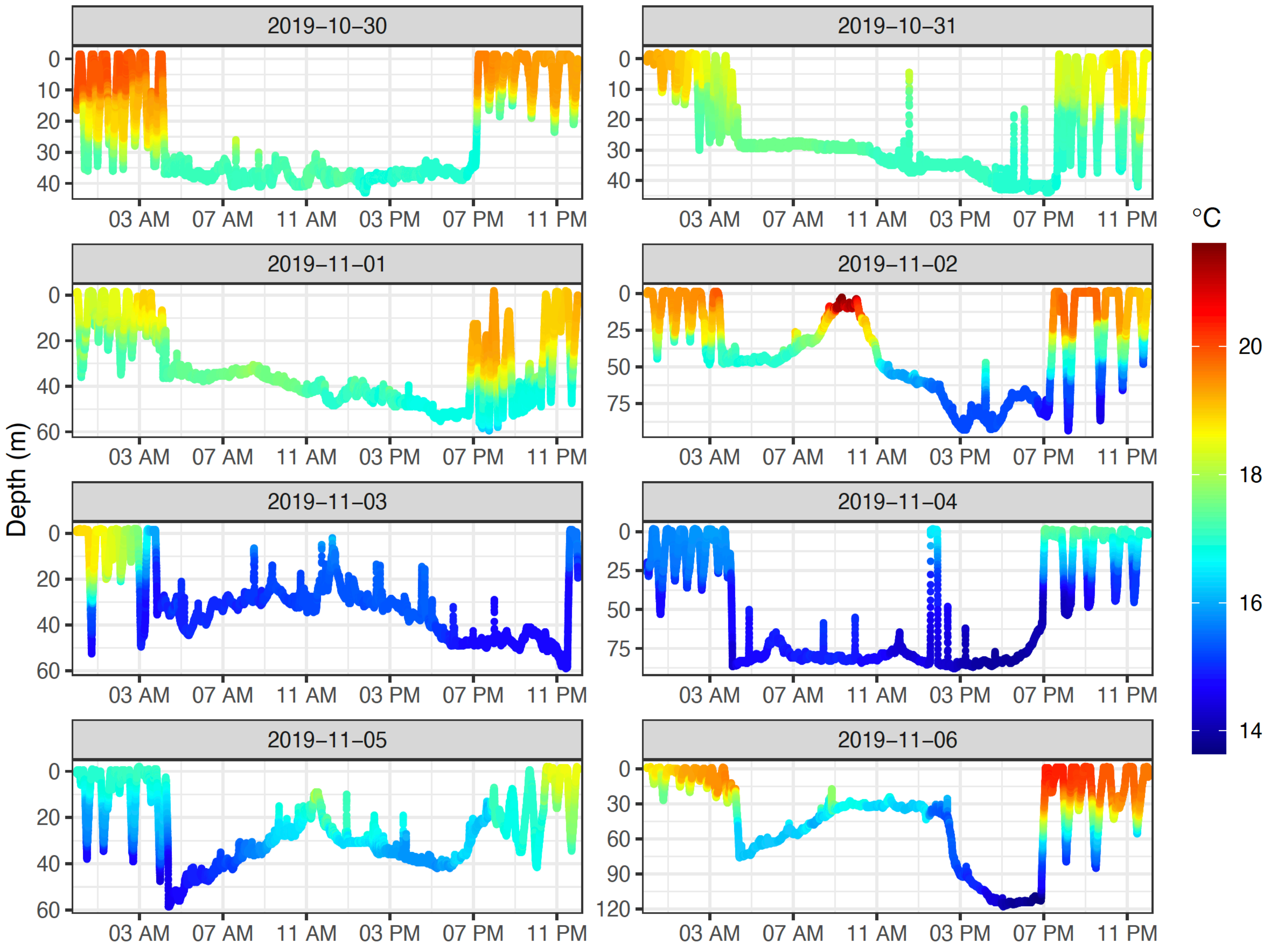
2019–10–30
2019–10–31
2019–11–01
2019–11–02
2019–11–03
2019–11–04
2019–11–05
2019–11–06
Depth (m)
03 AM
07 AM
11 AM
03 PM
07 PM
11 PM
°C
20
18
16
14

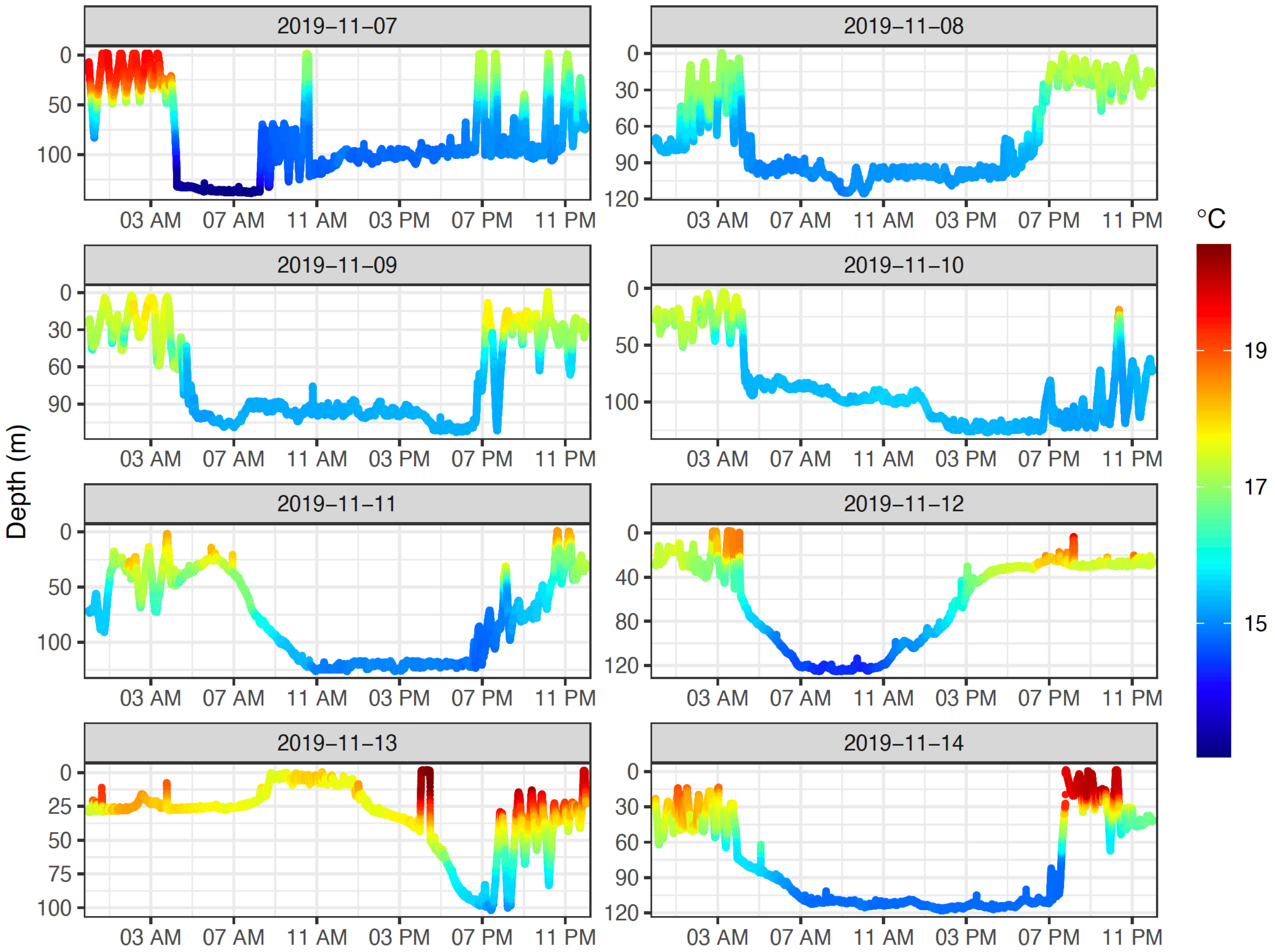
2019–11–07
2019–11–08
2019–11–09
2019–11–10
2019–11–11
2019–11–12
2019–11–13
2019–11–14
Depth (m)
03 AM
07 AM
11 AM
03 PM
07 PM
11 PM
°C
19
17
15

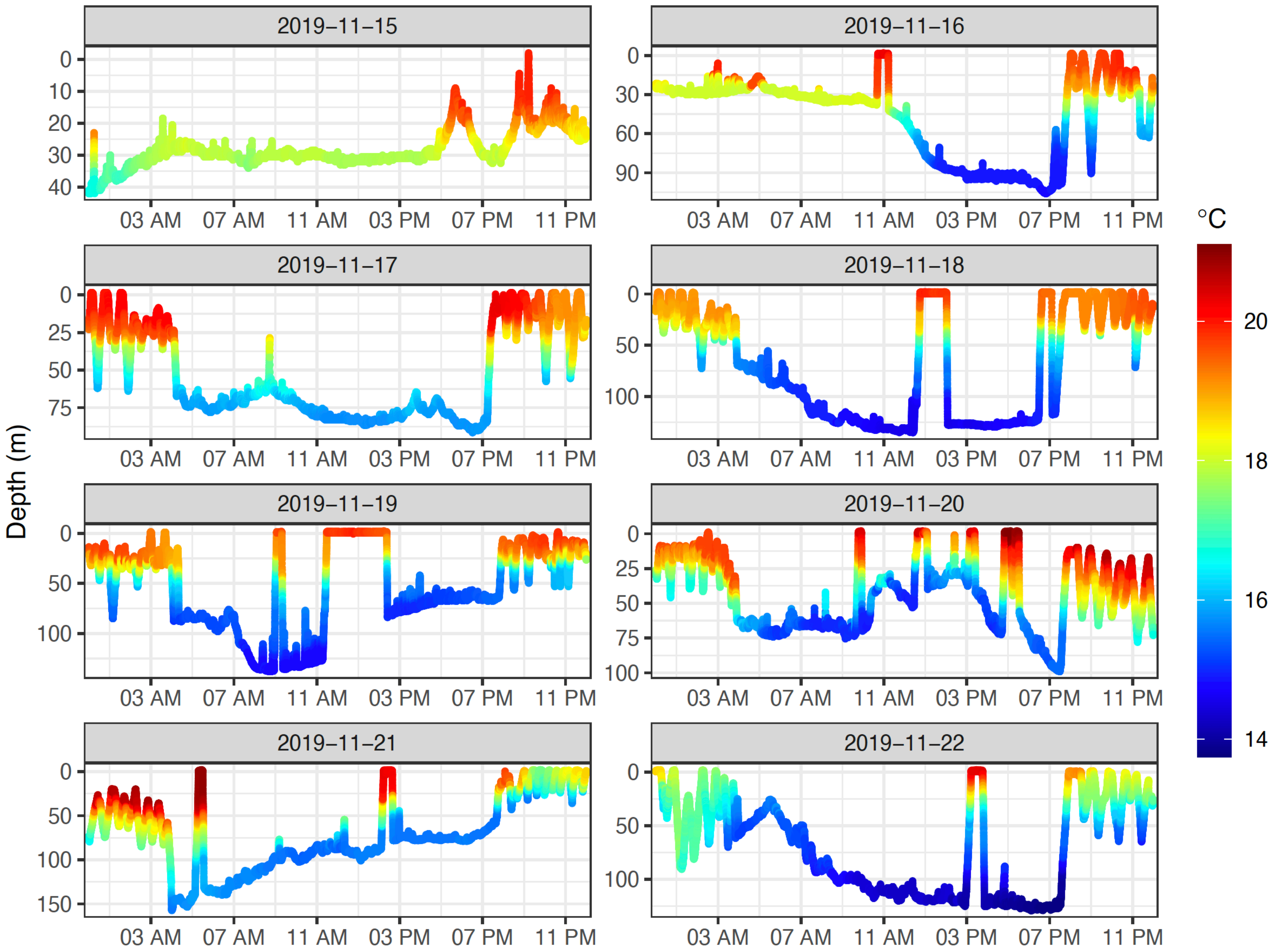

2019–11–15
2019–11–16
2019–11–17
2019–11–18
2019–11–19
2019–11–20
2019–11–21
2019–11–22
Depth (m)
03 AM
07 AM
11 AM
03 PM
07 PM
11 PM
°C
14
16
18
20

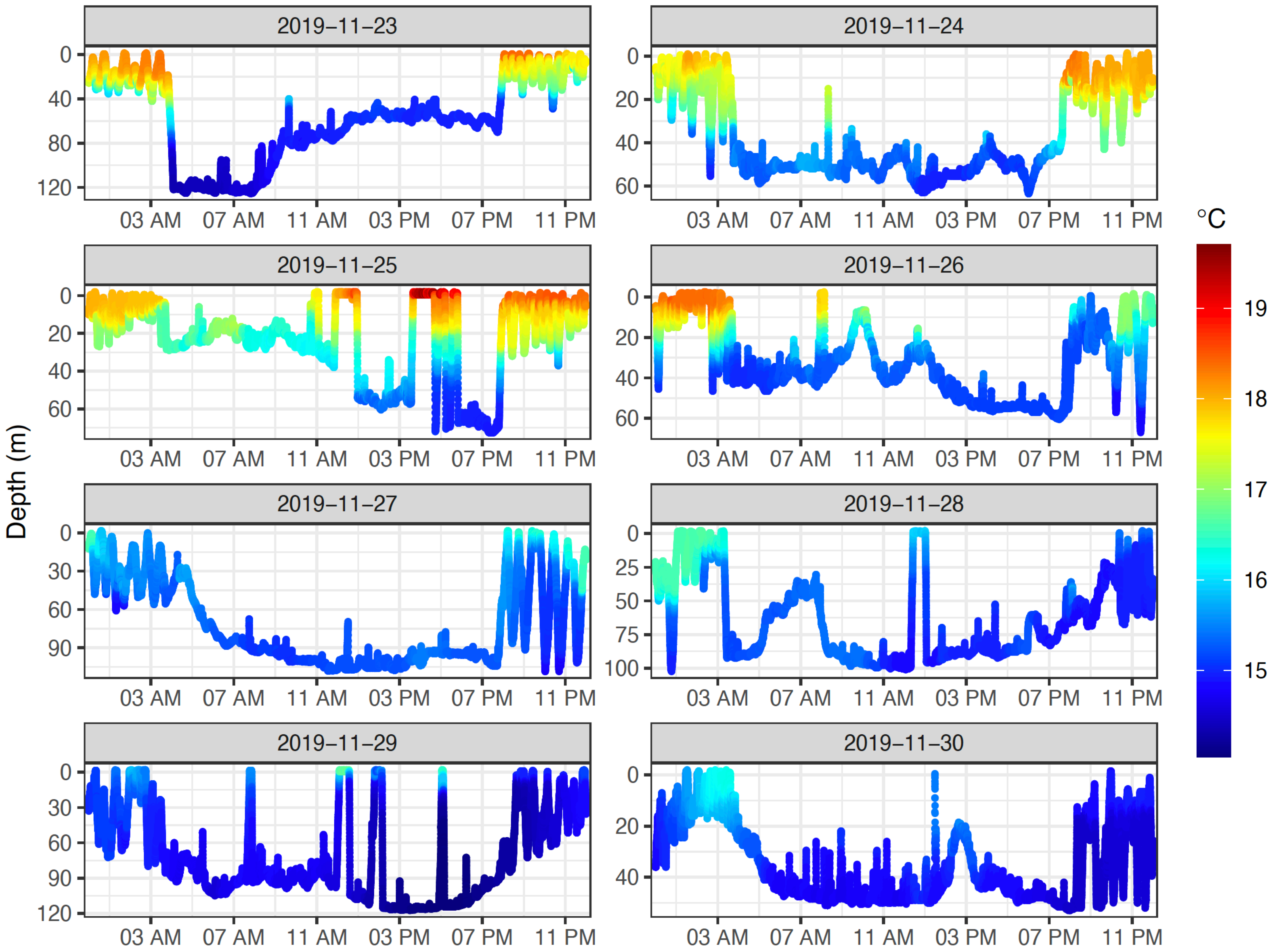
2019–11–23
2019–11–24
2019–11–25
2019–11–26
2019–11–27
2019–11–28
2019–11–29
2019–11–30
03 AM
07 AM
11 AM
03 PM
07 PM
11 PM
Depth (m)
°C
19
18
17
16
15

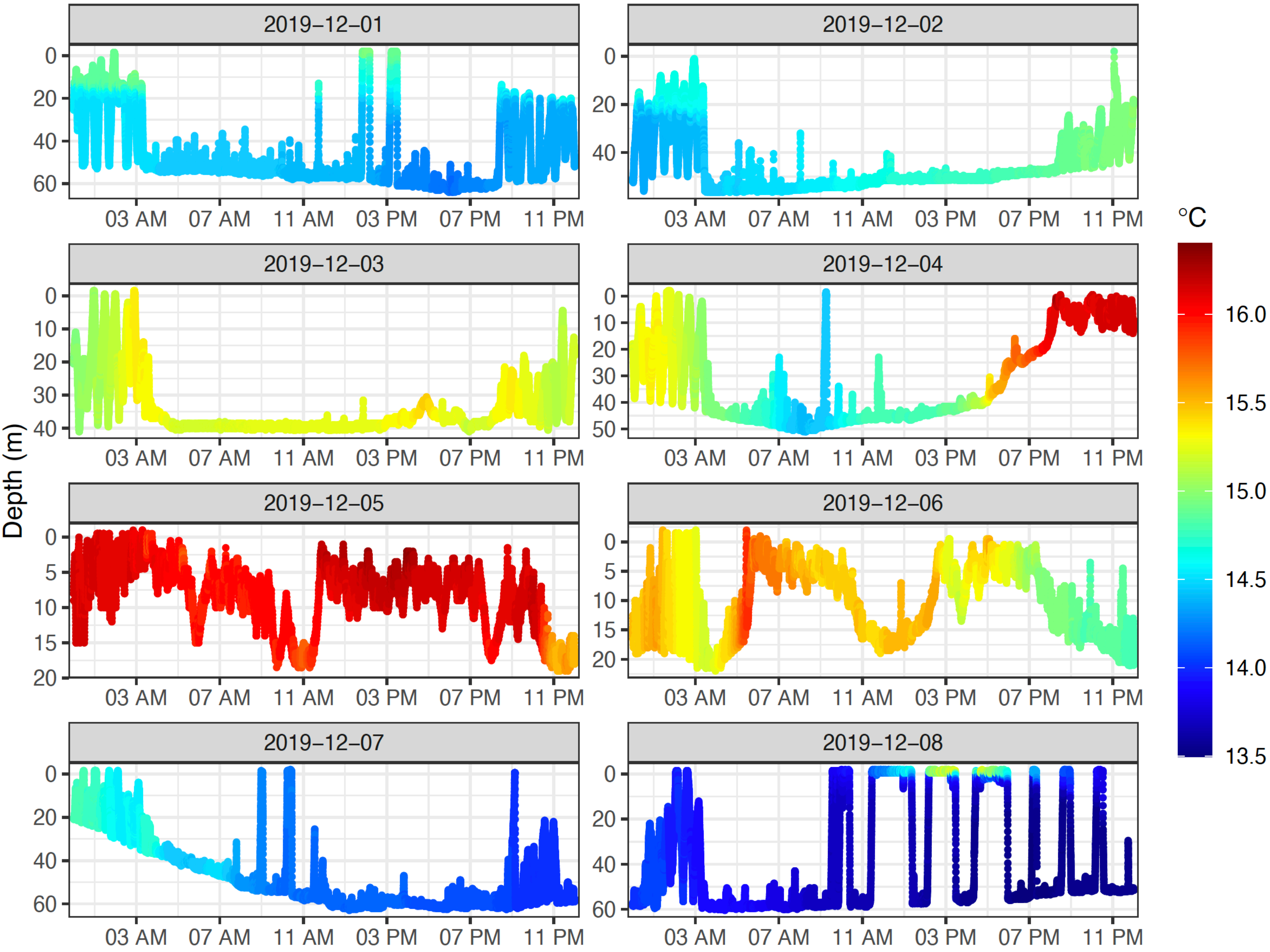

2019–12–01
2019–12–02
2019–12–03
2019–12–04
2019–12–05
2019–12–06
2019–12–07
2019–12–08
Depth (m)
03 AM
07 AM
11 AM
03 PM
07 PM
11 PM
°C
16.0
15.5
15.0
14.5
14.0
13.5

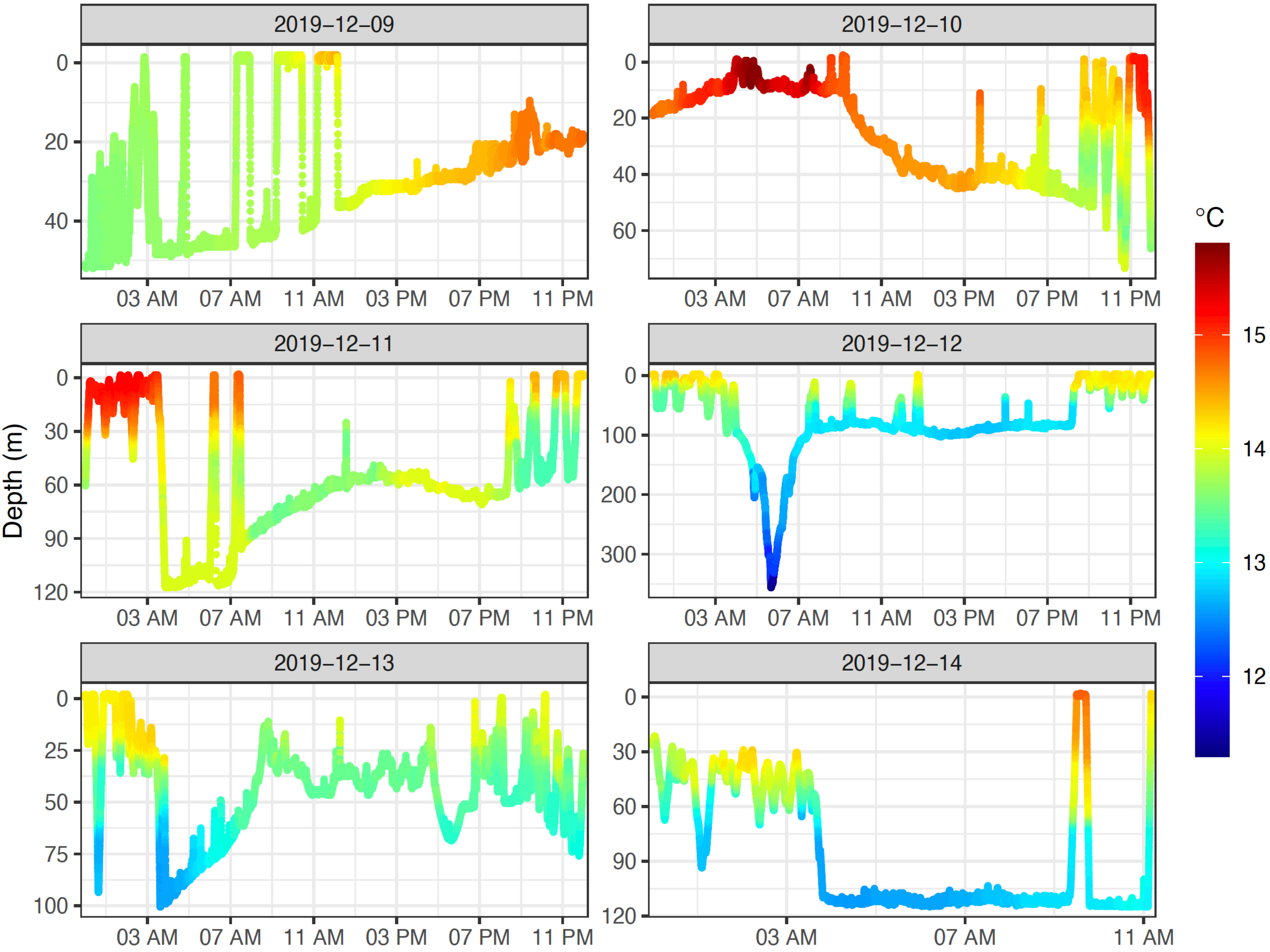
2019–12–09
2019–12–10
2019–12–11
2019–12–12
2019–12–13
2019–12–14
Depth (m)
°C
15
14
13
12
03 AM
07 AM
11 AM
03 PM
07 PM
11 PM

Shark 365

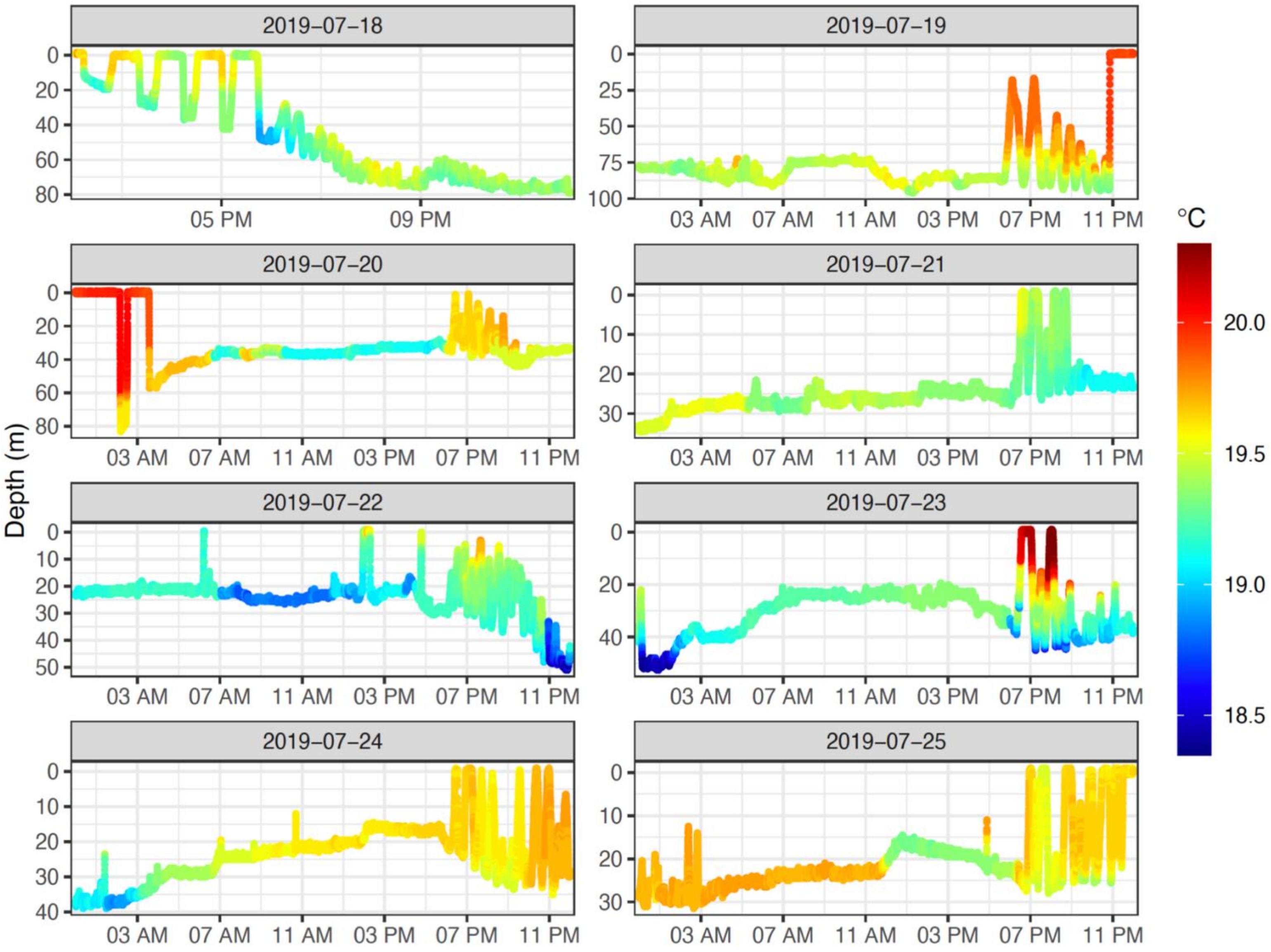
2019−07−18
2019−07−19
2019−07−20
2019−07−21
2019−07−22
2019−07−23
2019−07−24
2019−07−25
Depth (m)
°C
20.0
19.5
19.0
18.5
05 PM
09 PM
03 AM
07 AM
11 AM
03 PM
07 PM
11 PM

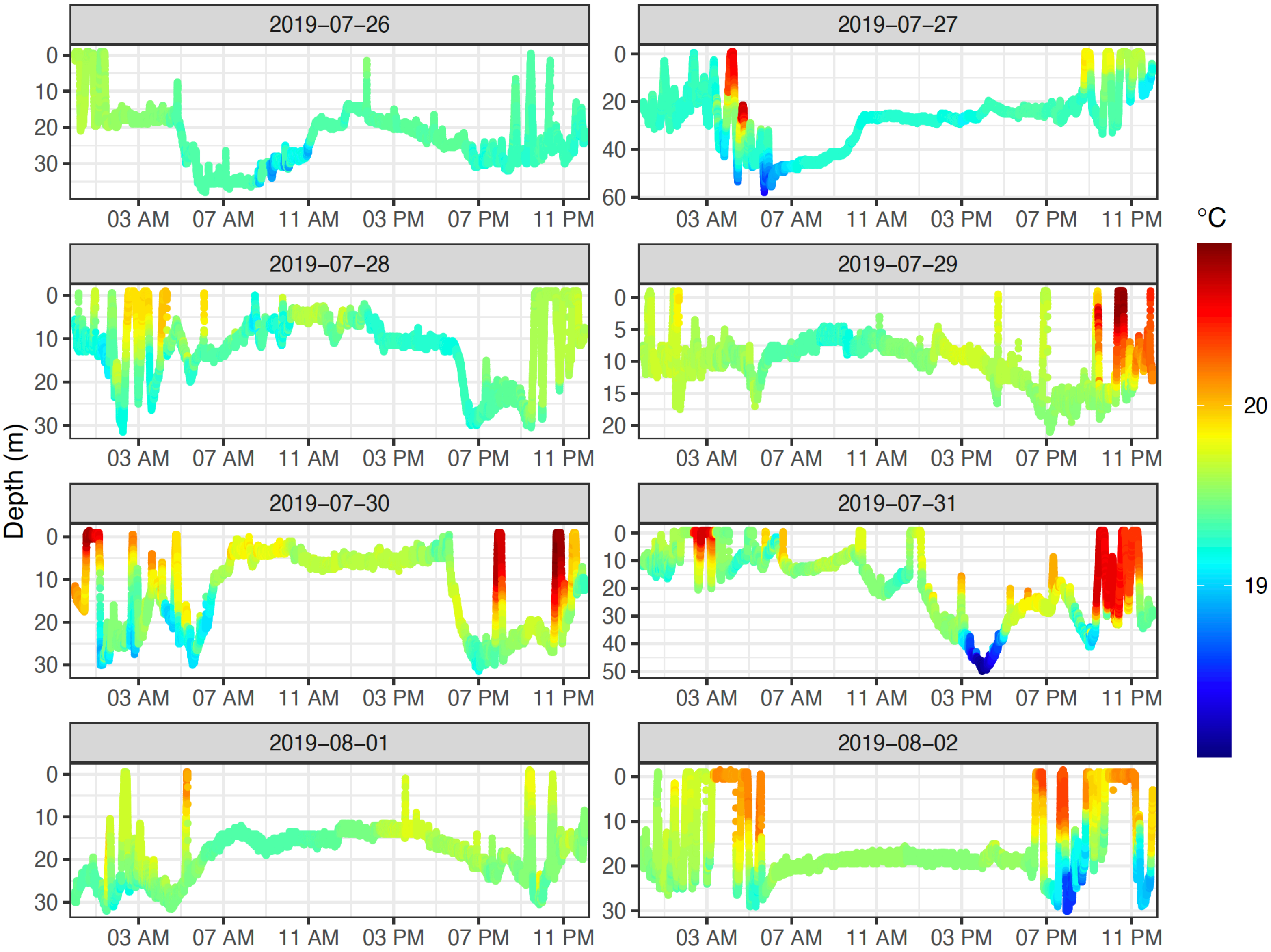

2019−07−26
2019−07−27
2019−07−28
2019−07−29
2019−07−30
2019−07−31
2019−08−01
2019−08−02
Depth (m)
03 AM
07 AM
11 AM
03 PM
07 PM
11 PM
°C
20
19

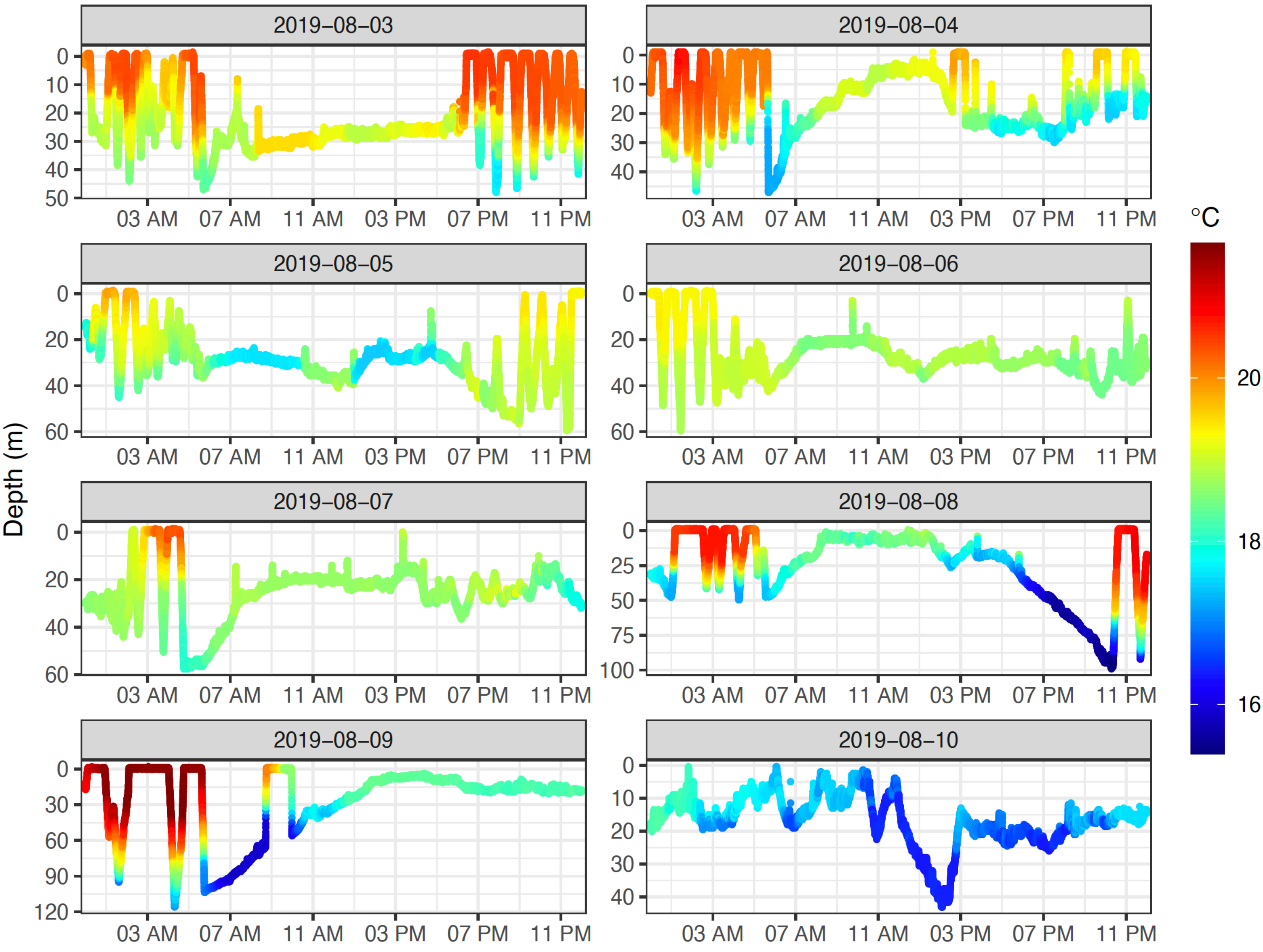

2019–08–03
2019–08–04
2019–08–05
2019–08–06
2019–08–07
2019–08–08
2019–08–09
2019–08–10
Depth (m)
03 AM
07 AM
11 AM
03 PM
07 PM
11 PM
°C
20
18
16

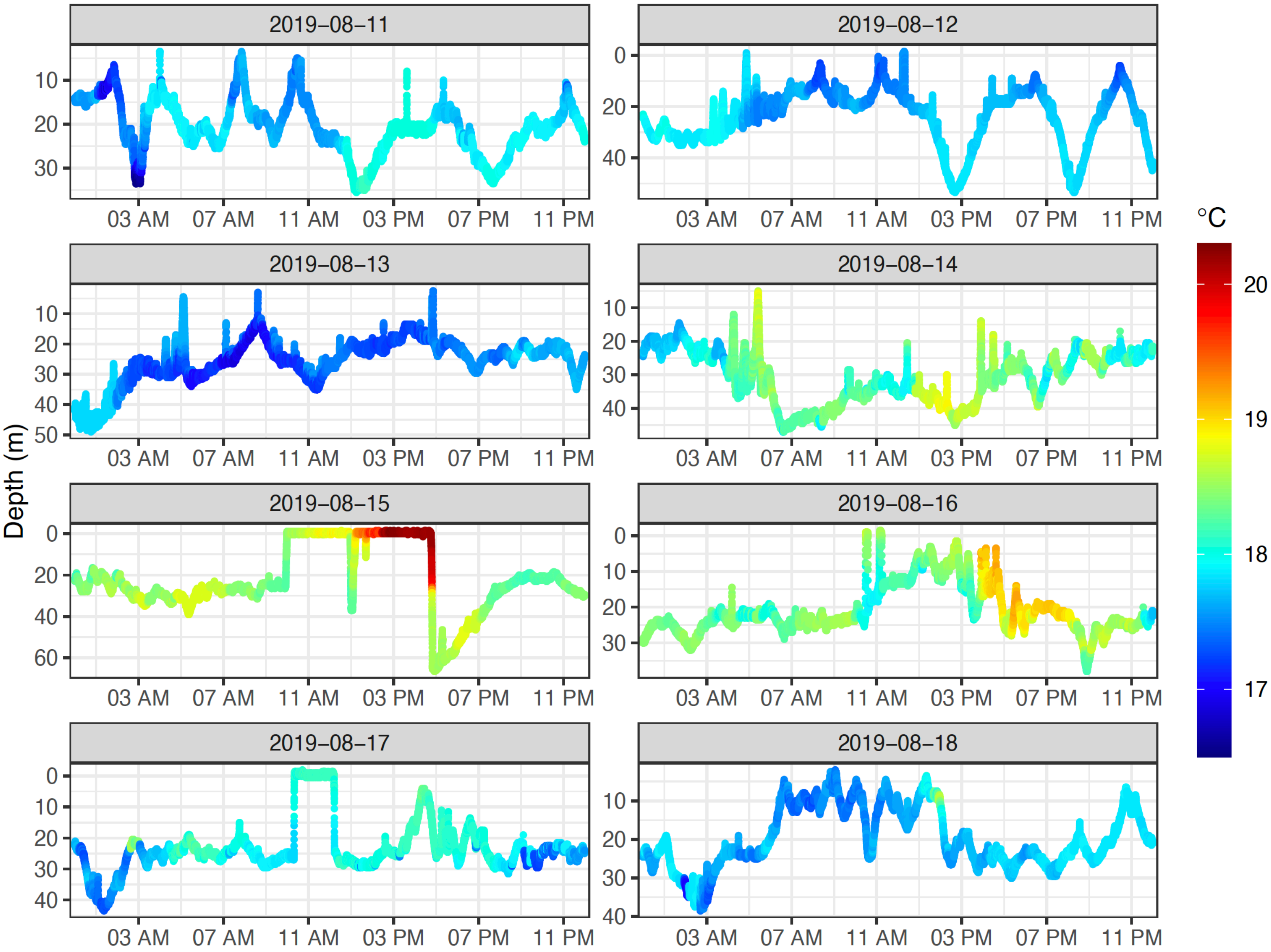

2019−08−11
2019−08−12
2019−08−13
2019−08−14
2019−08−15
2019−08−16
2019−08−17
2019−08−18
Depth (m)
03 AM
07 AM
11 AM
03 PM
07 PM
11 PM
°C
20
19
18
17

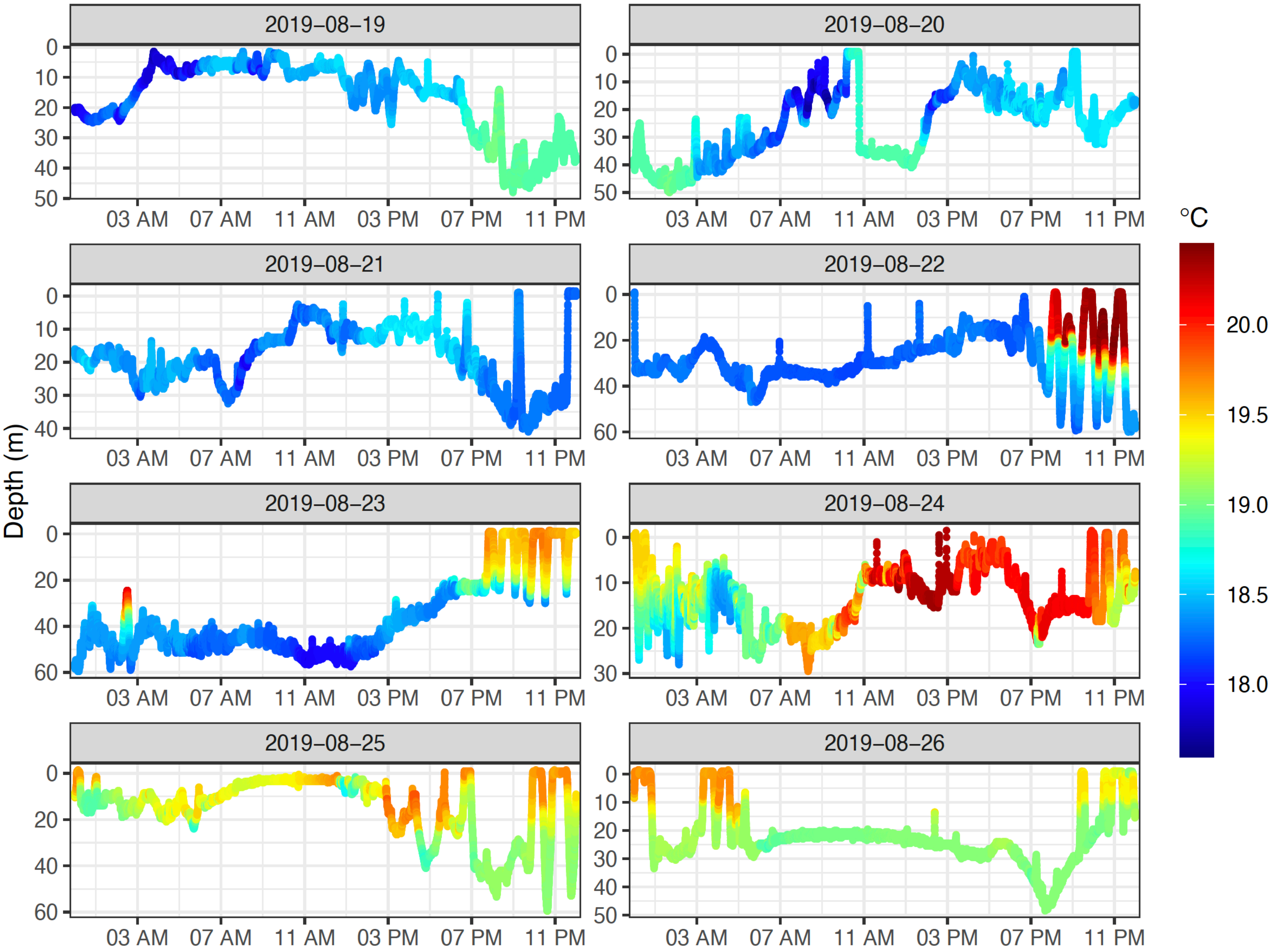

2019–08–19
2019–08–20
2019–08–21
2019–08–22
2019–08–23
2019–08–24
2019–08–25
2019–08–26
Depth (m)
03 AM
07 AM
11 AM
03 PM
07 PM
11 PM
°C
20.0
19.5
19.0
18.5
18.0

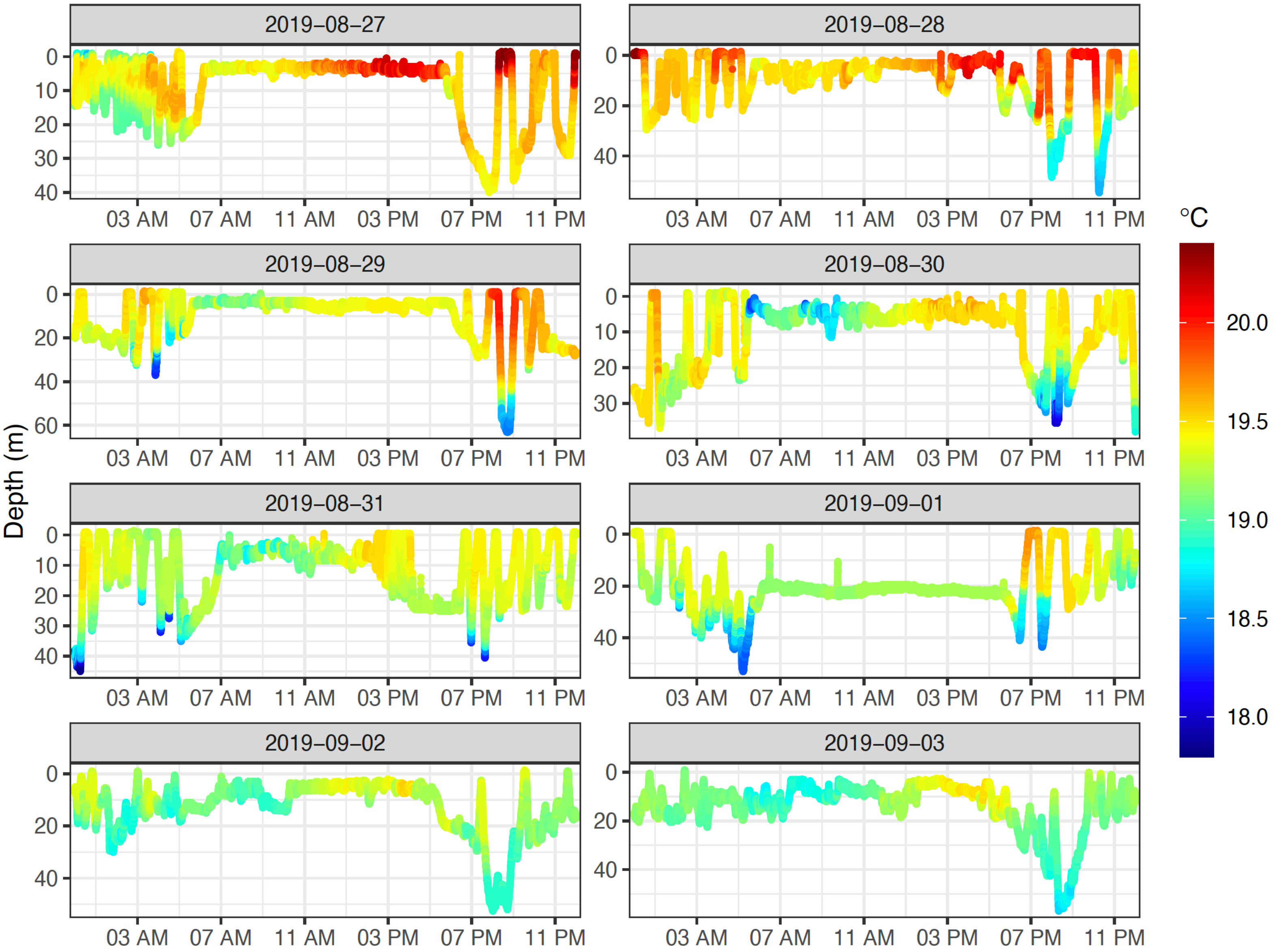
2019−08−27
2019−08−28
2019−08−29
2019−08−30
2019−08−31
2019−09−01
2019−09−02
2019−09−03
Depth (m)
03 AM
07 AM
11 AM
03 PM
07 PM
11 PM
°C
20.0
19.5
19.0
18.5
18.0

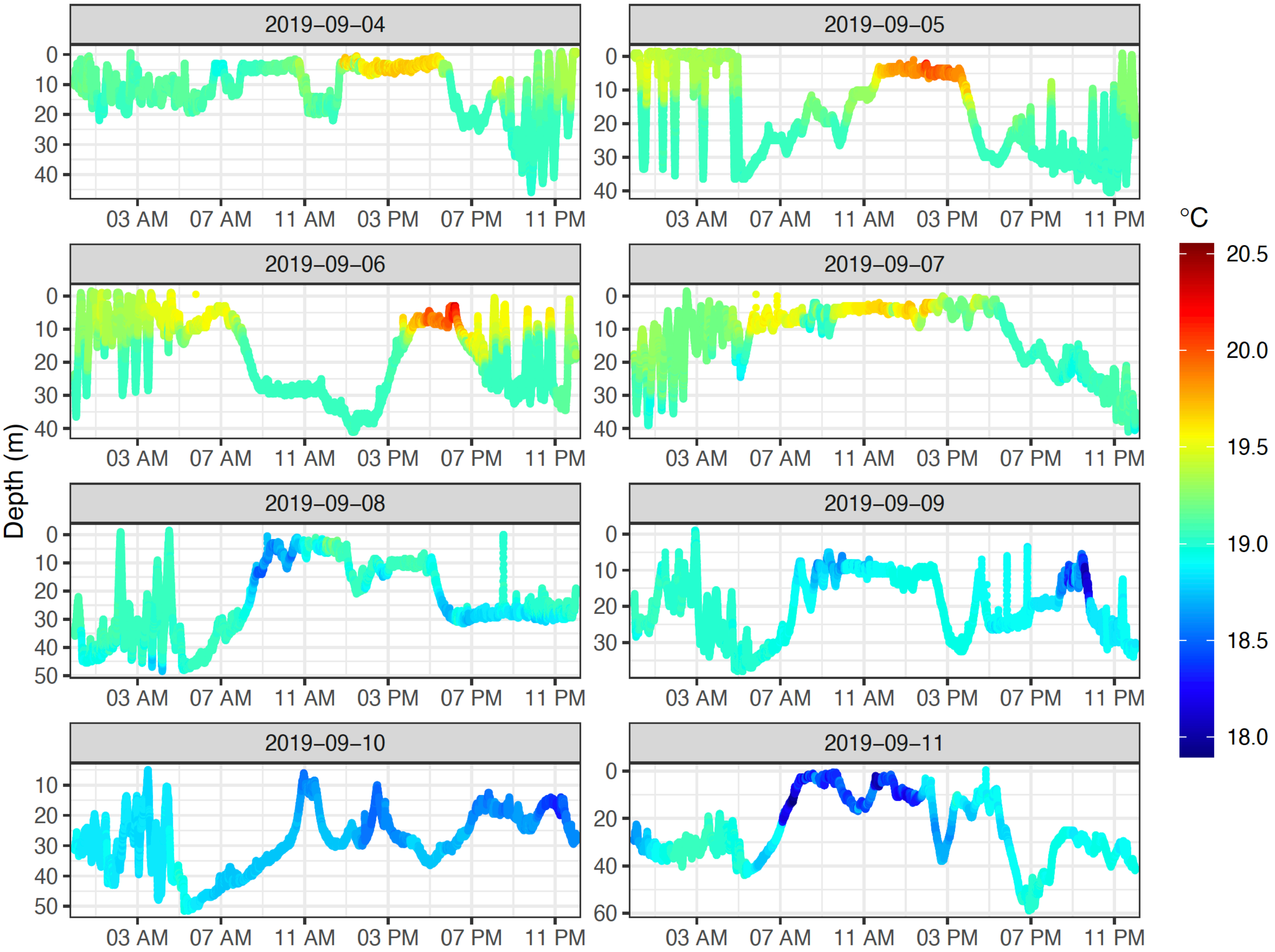
2019–09–04
2019–09–05
2019–09–06
2019–09–07
2019–09–08
2019–09–09
2019–09–10
2019–09–11
Depth (m)
03 AM
07 AM
11 AM
03 PM
07 PM
11 PM
°C
20.5
20.0
19.5
19.0
18.5
18.0

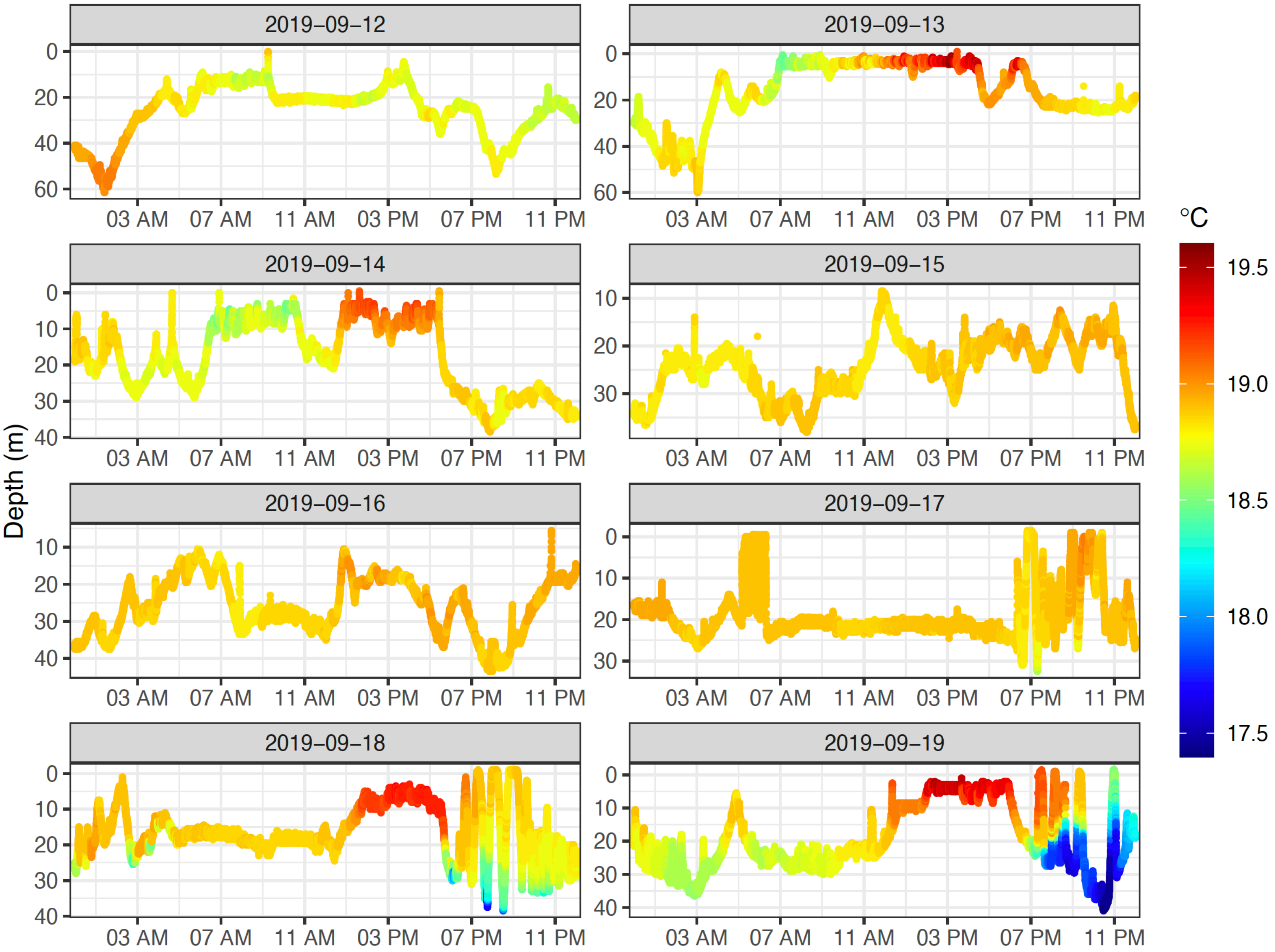

2019–09–12
2019–09–13
2019–09–14
2019–09–15
2019–09–16
2019–09–17
2019–09–18
2019–09–19
Depth (m)
03 AM
07 AM
11 AM
03 PM
07 PM
11 PM
°C
19.5
19.0
18.5
18.0
17.5

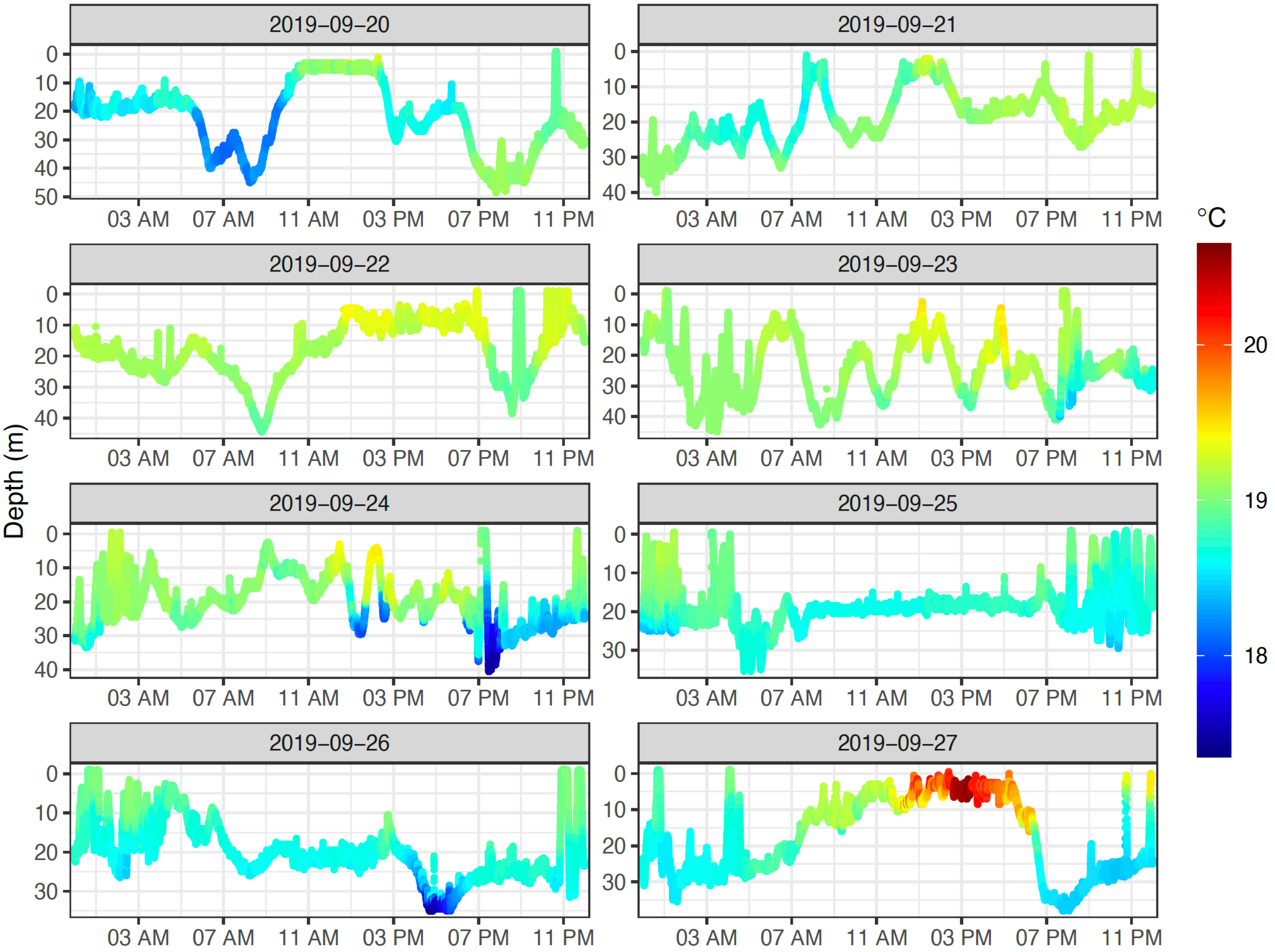

2019–09–20
2019–09–21
2019–09–22
2019–09–23
2019–09–24
2019–09–25
2019–09–26
2019–09–27
Depth (m)
03 AM
07 AM
11 AM
03 PM
07 PM
11 PM
°C
20
19
18

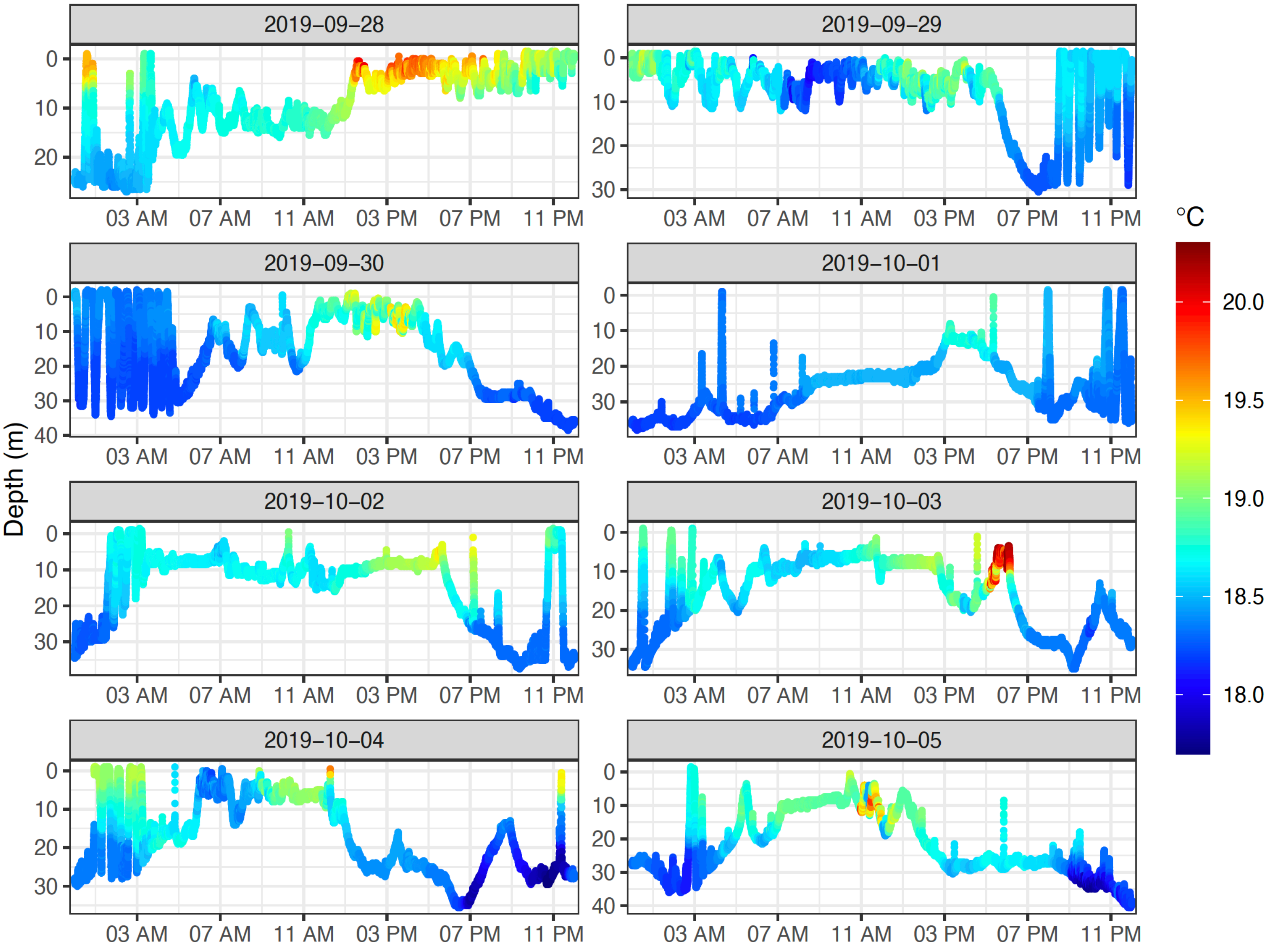

2019–09–28
2019–09–29
2019–09–30
2019–10–01
2019–10–02
2019–10–03
2019–10–04
2019–10–05
Depth (m)
0
10
20
30
40
03 AM
07 AM
11 AM
03 PM
07 PM
11 PM
°C
20.0
19.5
19.0
18.5
18.0

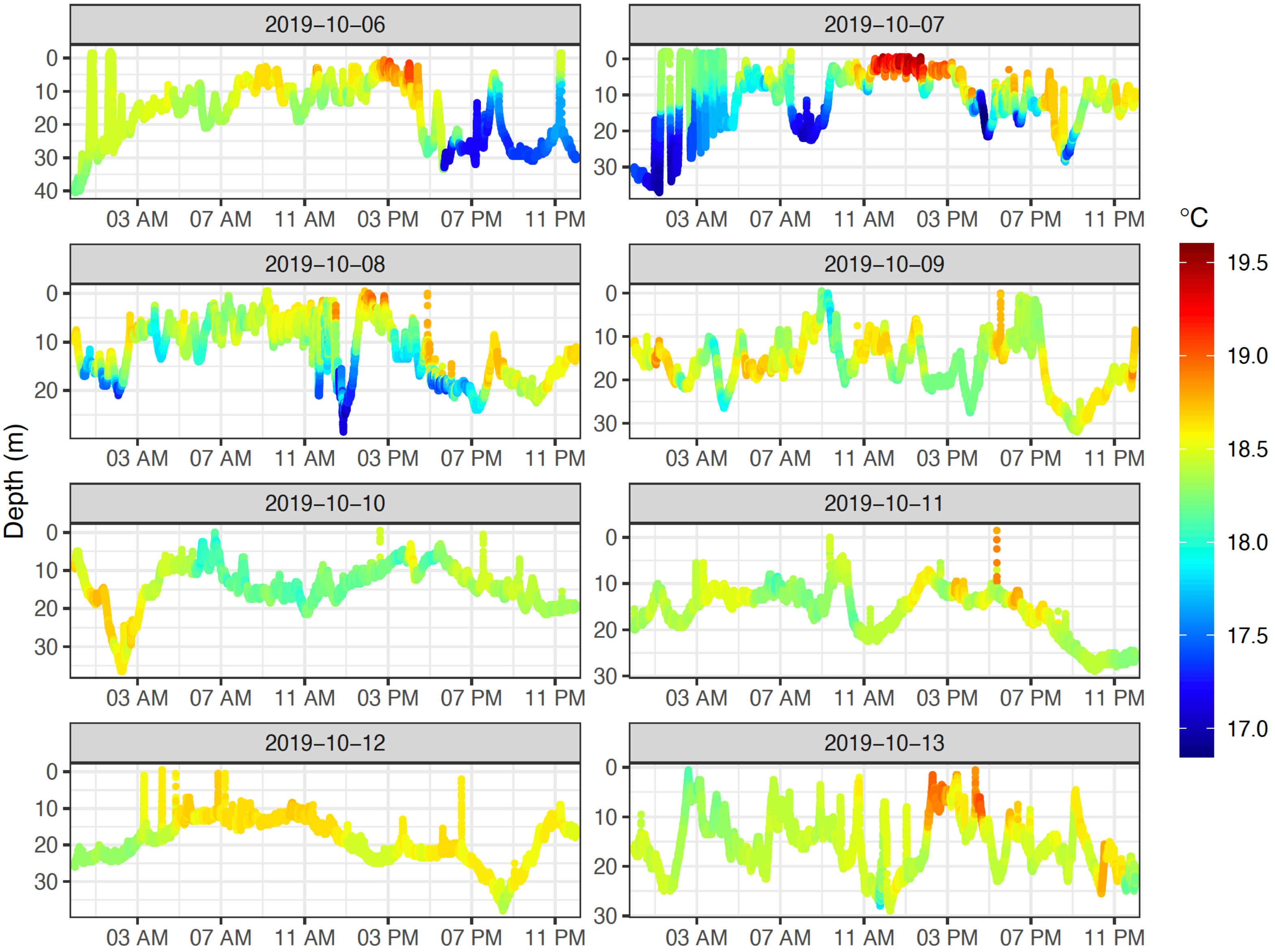
2019–10–06
2019–10–07
2019–10–08
2019–10–09
2019–10–10
2019–10–11
2019–10–12
2019–10–13
Depth (m)
03 AM
07 AM
11 AM
03 PM
07 PM
11 PM
°C
19.5
19.0
18.5
18.0
17.5
17.0

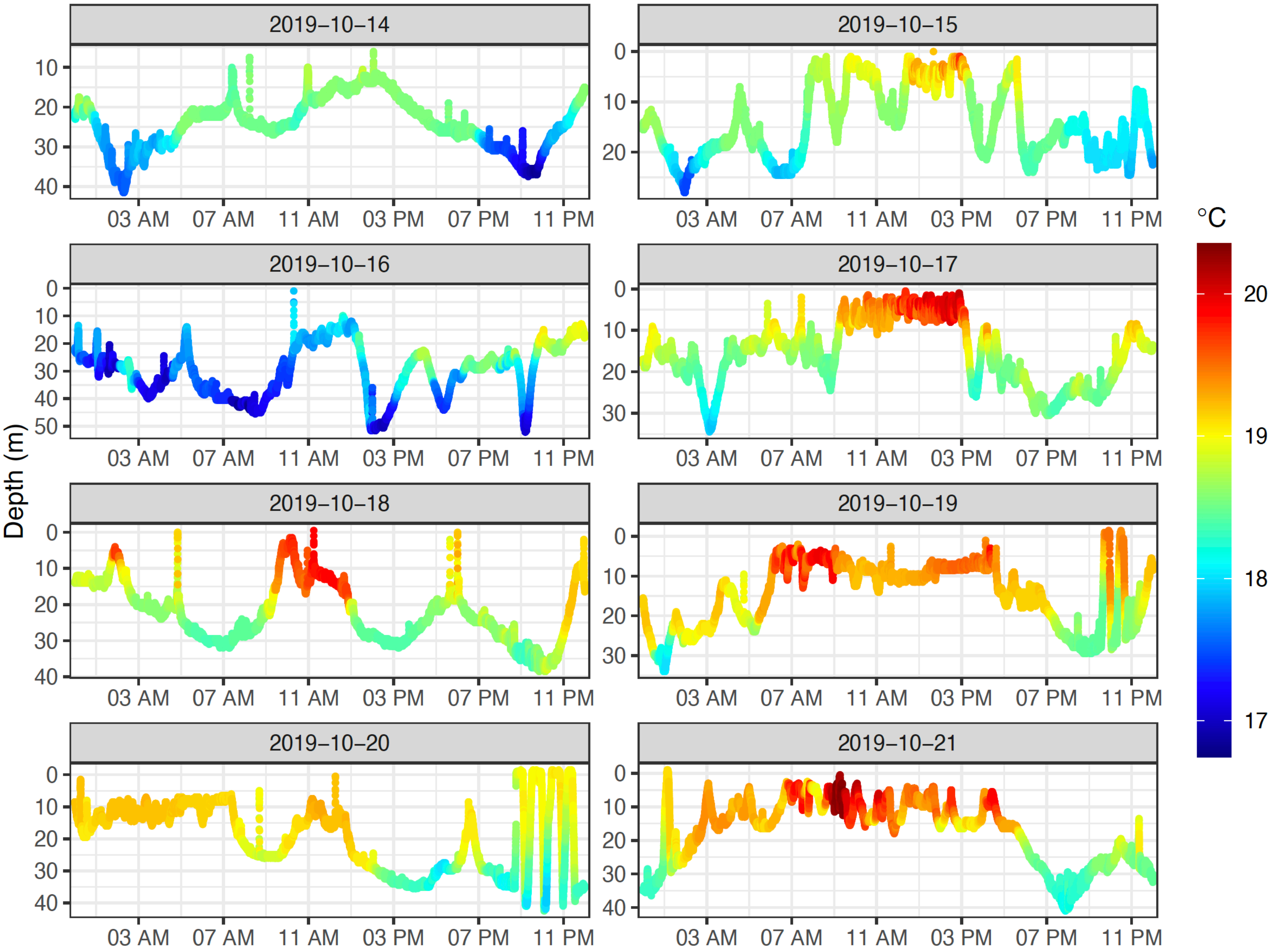

2019–10–14
2019–10–15
2019–10–16
2019–10–17
2019–10–18
2019–10–19
2019–10–20
2019–10–21
Depth (m)
03 AM
07 AM
11 AM
03 PM
07 PM
11 PM
°C
20
19
18
17

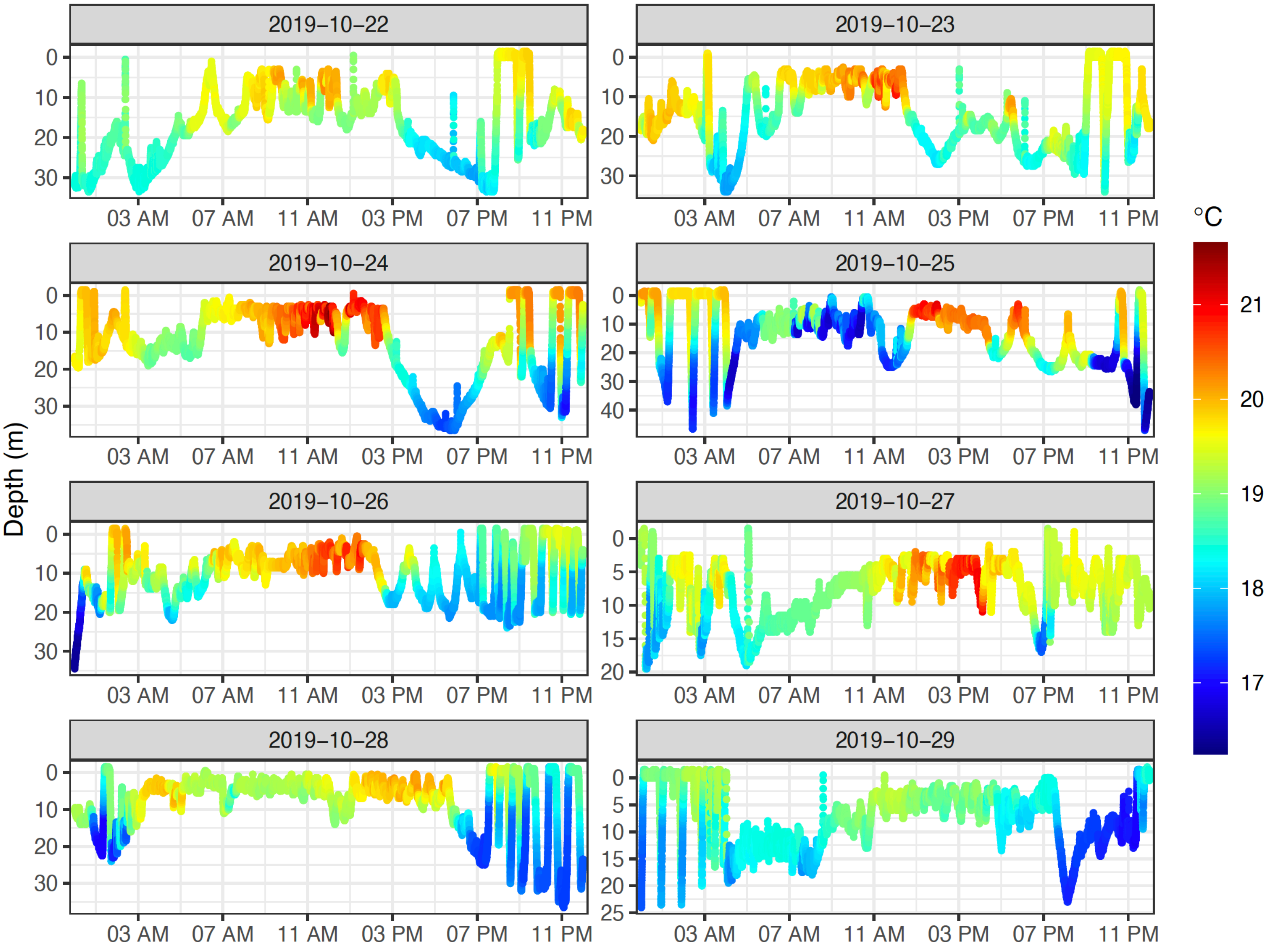

2019–10–22
2019–10–23
2019–10–24
2019–10–25
2019–10–26
2019–10–27
2019–10–28
2019–10–29
Depth (m)
03 AM
07 AM
11 AM
03 PM
07 PM
11 PM
°C
21
20
19
18
17

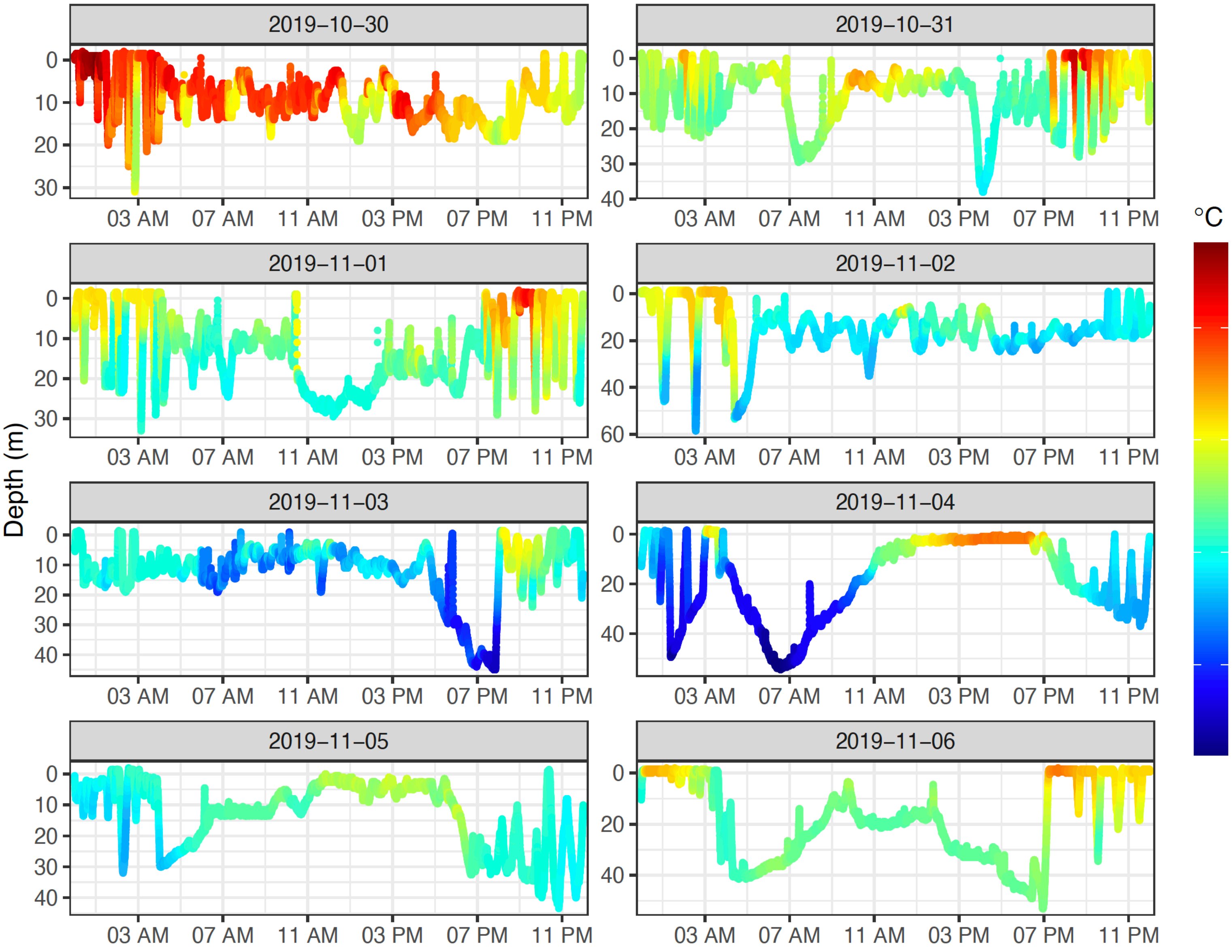
2019−10−30
2019−10−31
2019−11−01
2019−11−02
2019−11−03
2019−11−04
2019−11−05
2019−11−06
Depth (m)
03 AM
07 AM
11 AM
03 PM
07 PM
11 PM
°C
18
17
16
15

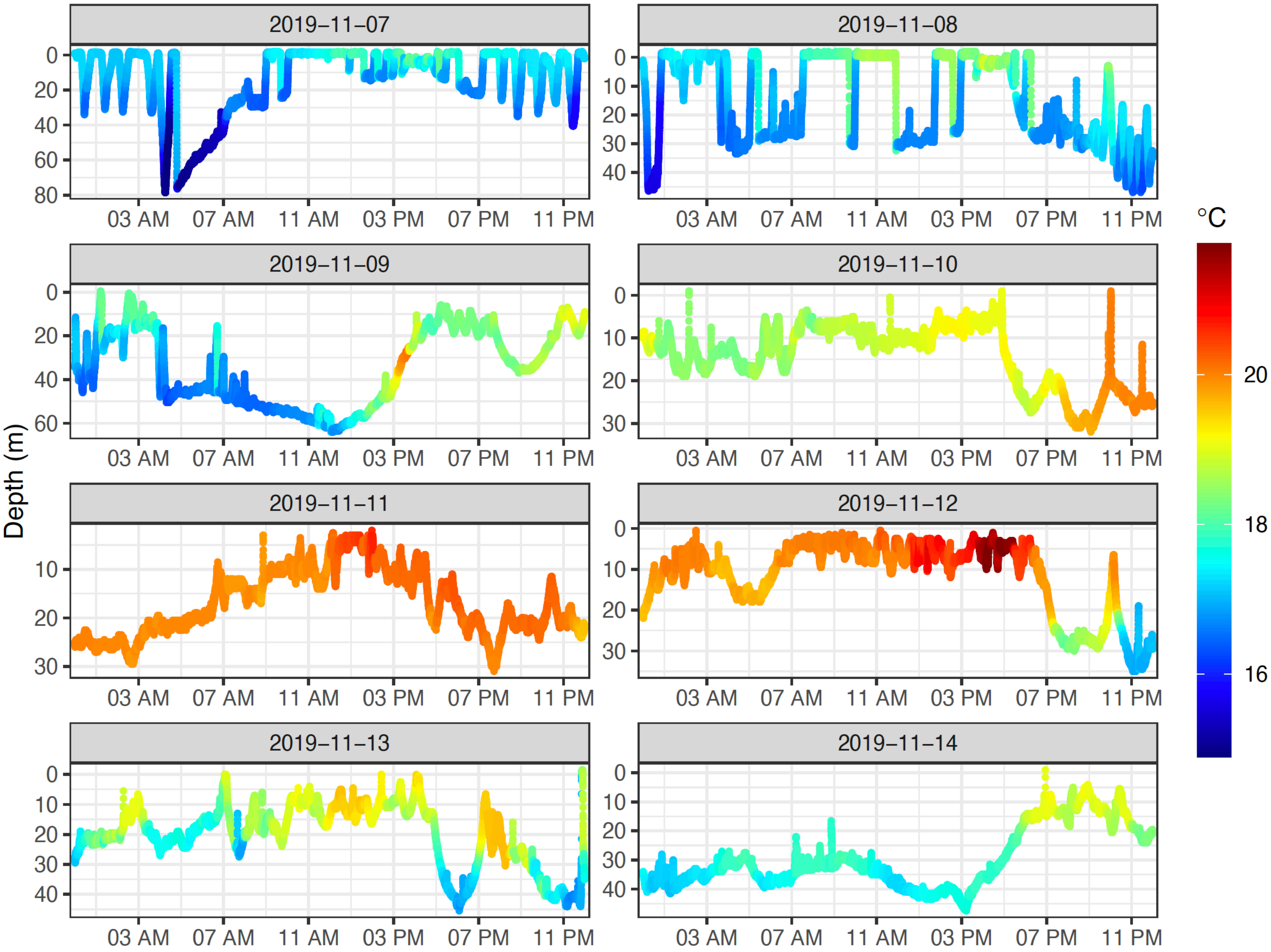
2019–11–07
2019–11–08
2019–11–09
2019–11–10
2019–11–11
2019–11–12
2019–11–13
2019–11–14
Depth (m)
03 AM
07 AM
11 AM
03 PM
07 PM
11 PM
°C
20
18
16

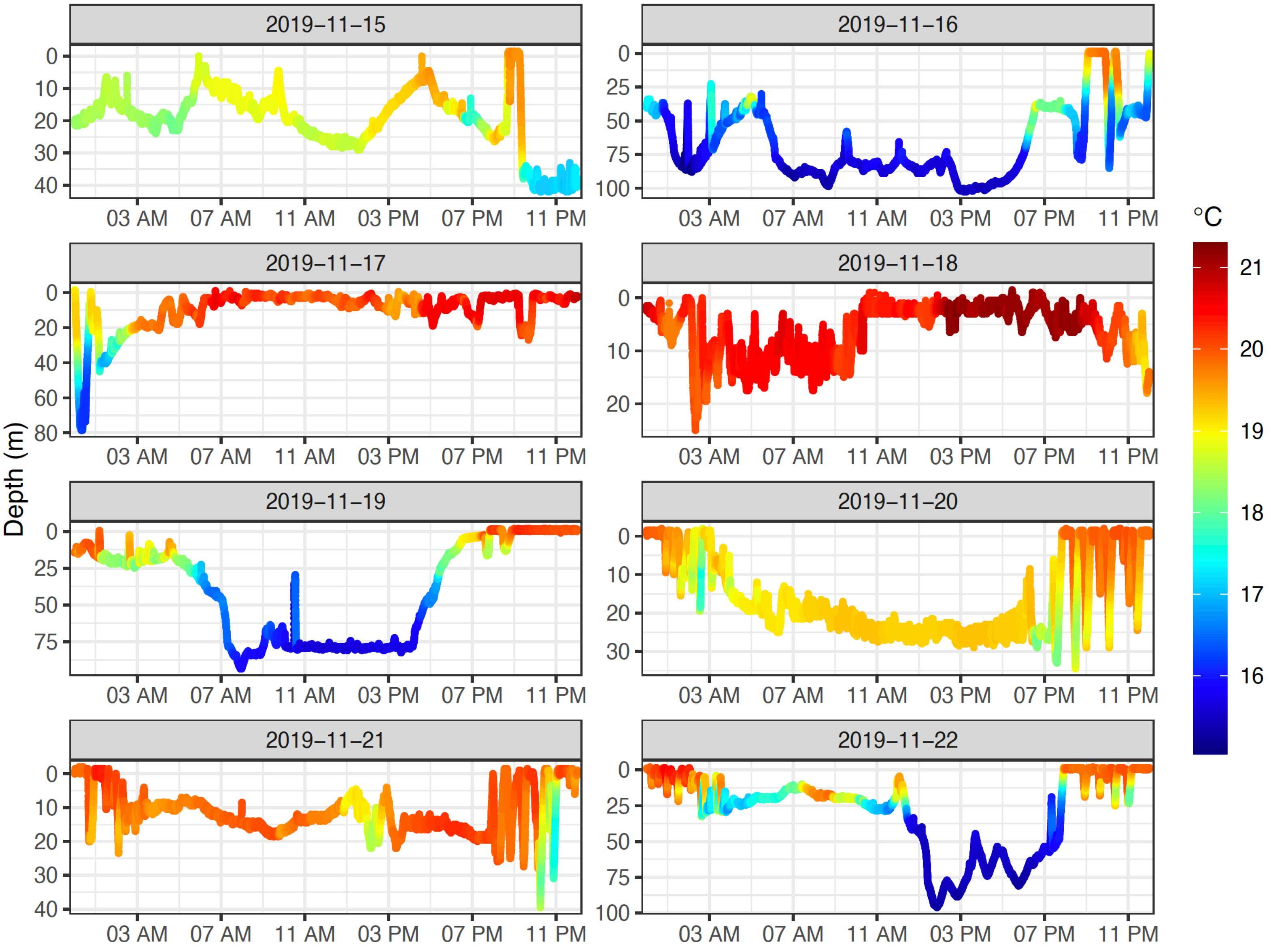

2019−11−15
2019−11−16
2019−11−17
2019−11−18
2019−11−19
2019−11−20
2019−11−21
2019−11−22
Depth (m)
°C
21
20
19
18
17
16
03 AM
07 AM
11 AM
03 PM
07 PM
11 PM

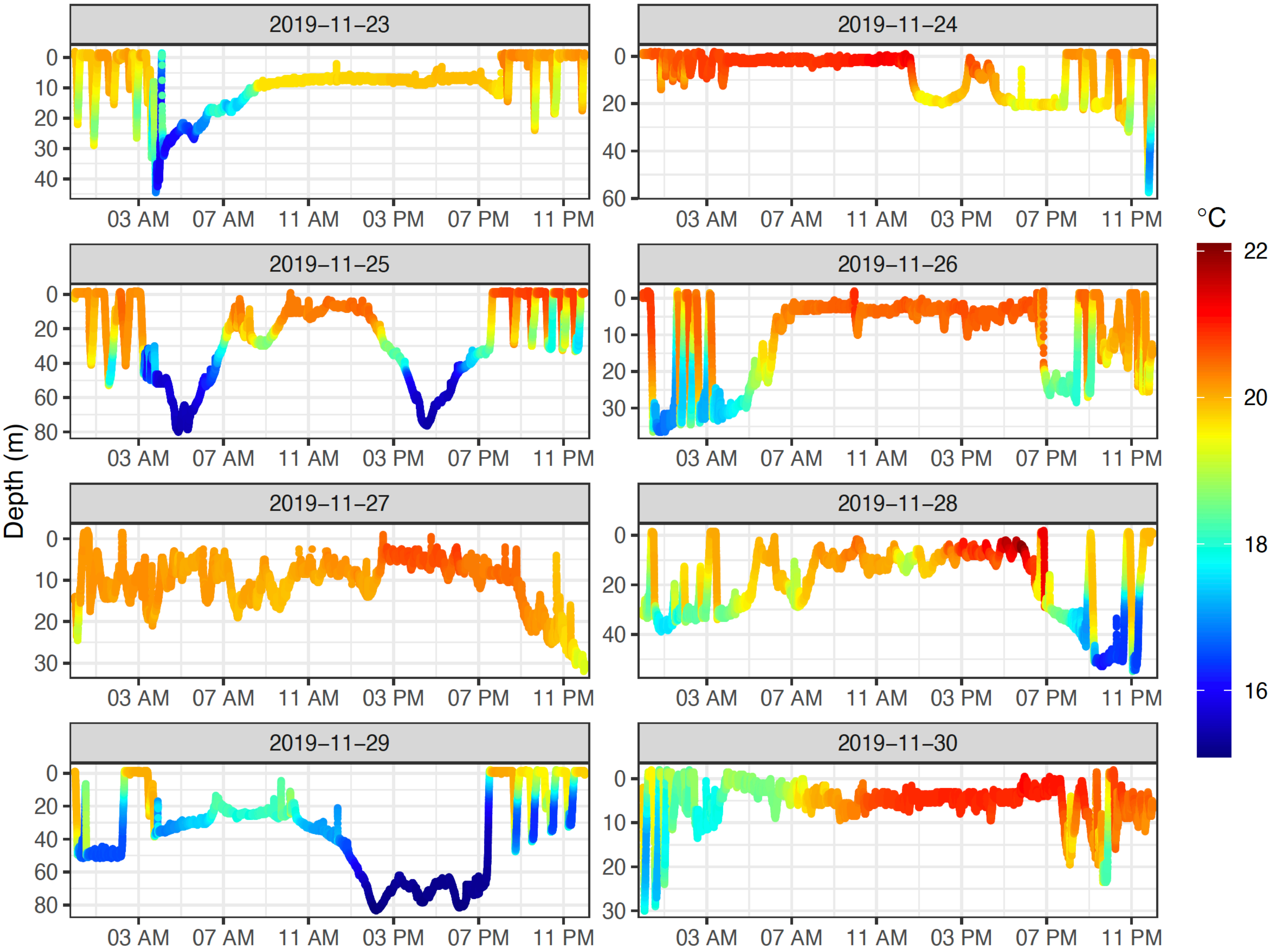

2019–11–23
2019–11–24
2019–11–25
2019–11–26
2019–11–27
2019–11–28
2019–11–29
2019–11–30
Depth (m)
03 AM
07 AM
11 AM
03 PM
07 PM
11 PM
°C
22
20
18
16

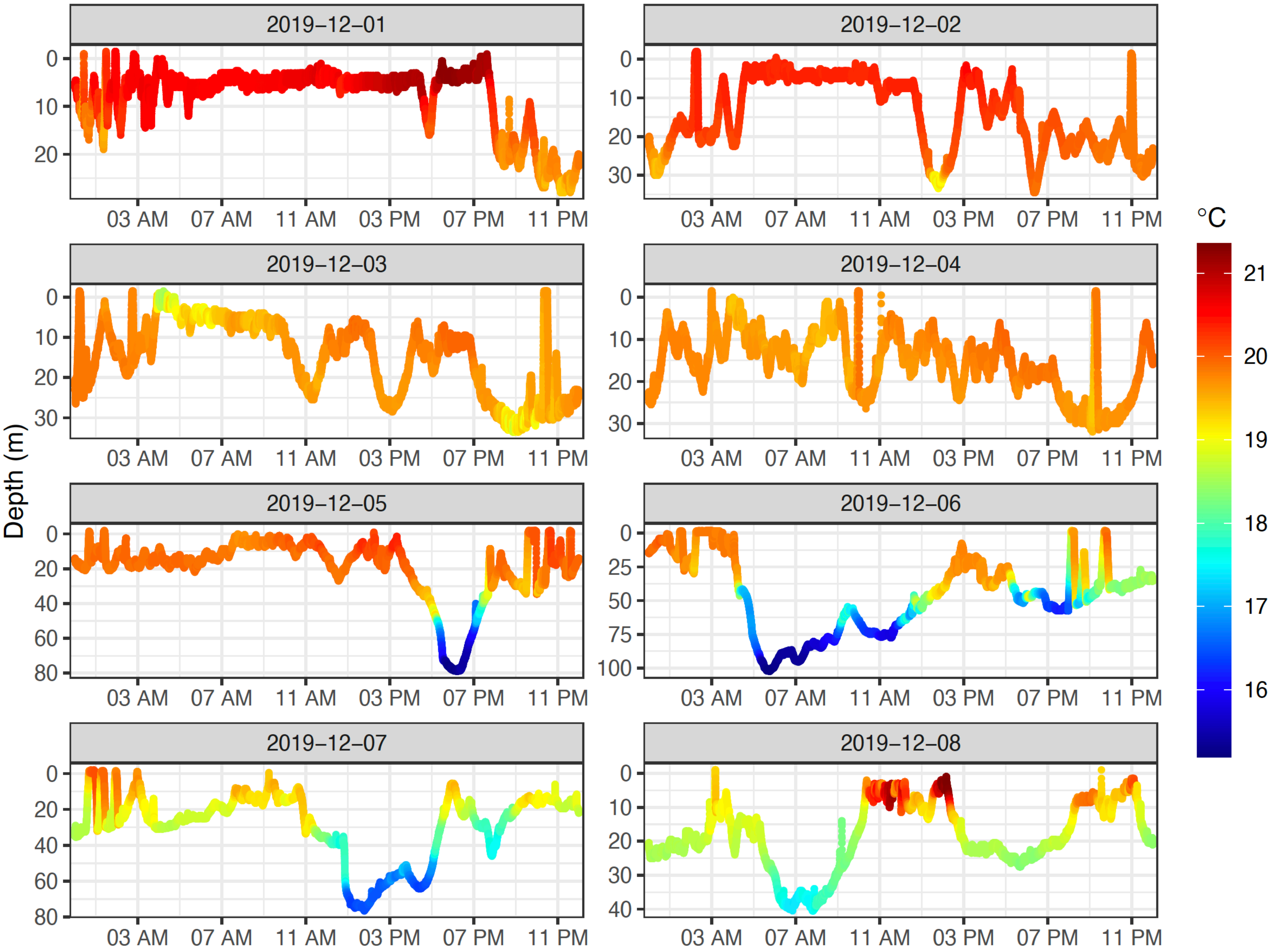

2019–12–01
2019–12–02
2019–12–03
2019–12–04
2019–12–05
2019–12–06
2019–12–07
2019–12–08
Depth (m)
03 AM
07 AM
11 AM
03 PM
07 PM
11 PM
°C
21
20
19
18
17
16

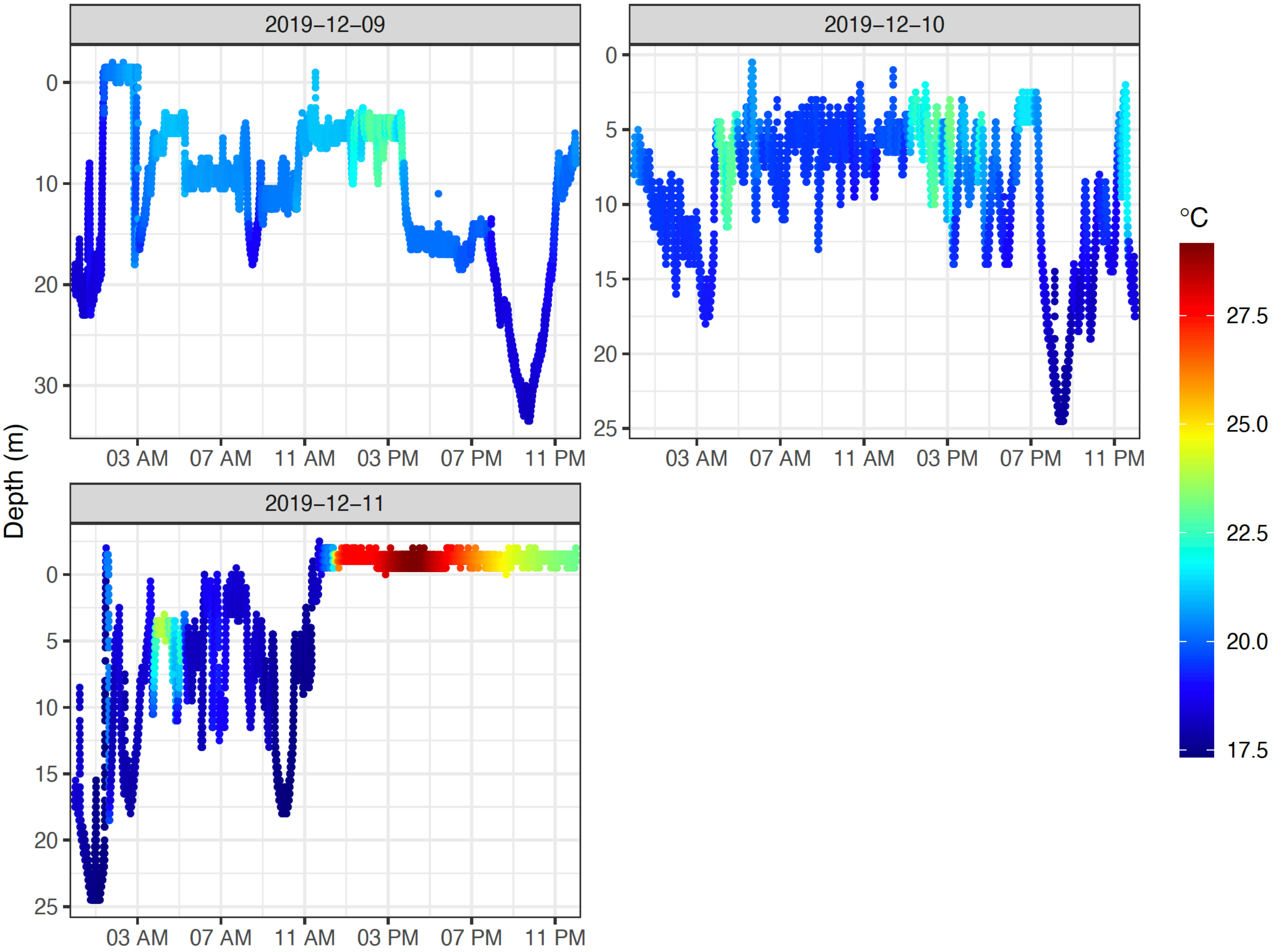

2019–12–09
2019–12–10
2019–12–11
Depth (m)
0
10
20
30
5
15
25
03 AM
07 AM
11 AM
03 PM
07 PM
11 PM
°C
27.5
25.0
22.5
20.0
17.5

Shark 366

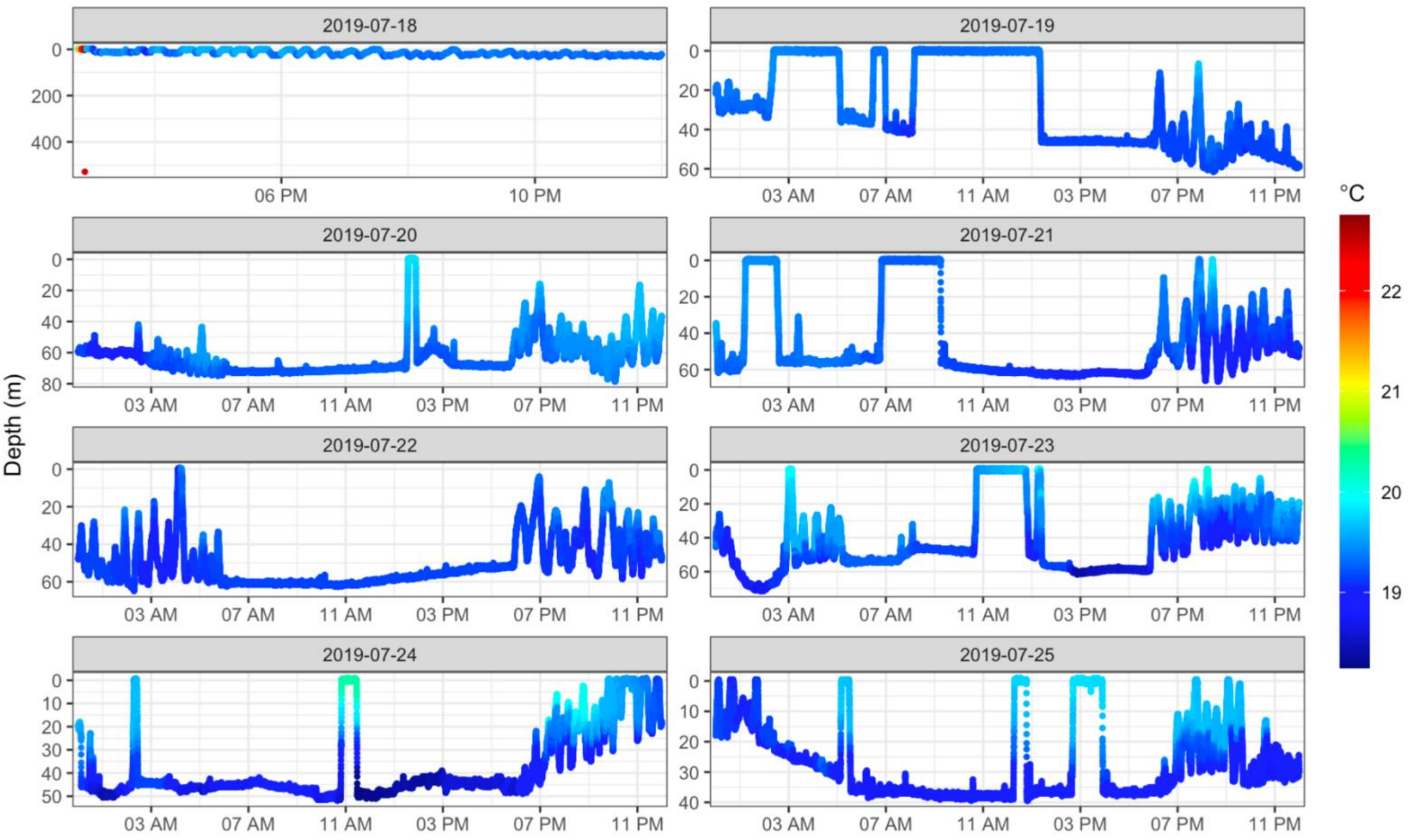

2019-07-18
2019-07-19
2019-07-20
2019-07-21
2019-07-22
2019-07-23
2019-07-24
2019-07-25
Depth (m)
°C
22
21
20
19
06 PM
10 PM
03 AM
07 AM
11 AM
03 PM
07 PM
11 PM

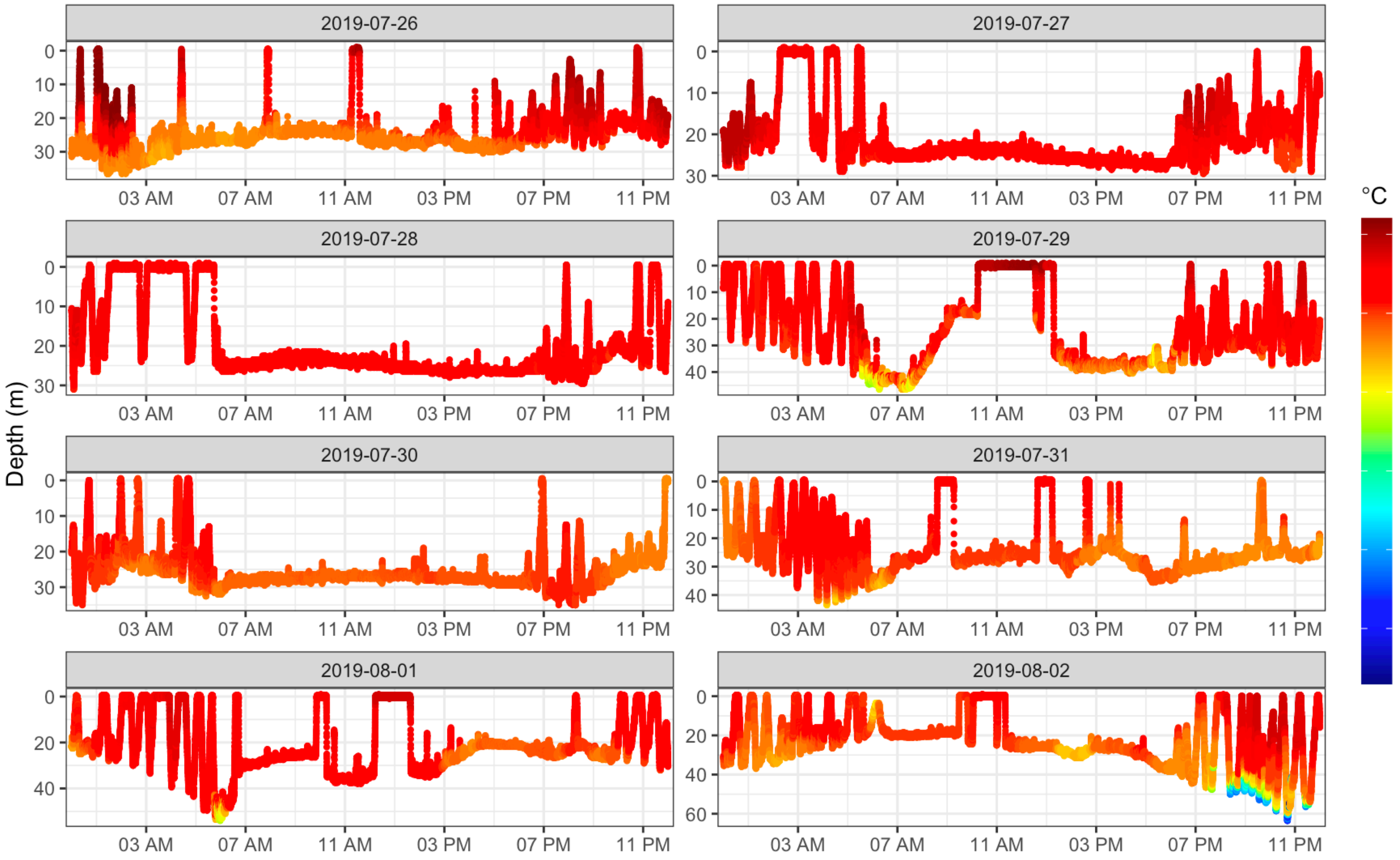
2019-07-26
2019-07-27
2019-07-28
2019-07-29
2019-07-30
2019-07-31
2019-08-01
2019-08-02
Depth (m)
03 AM
07 AM
11 AM
03 PM
07 PM
11 PM
°C
19.5
19.0
18.5
18.0
17.5
17.0

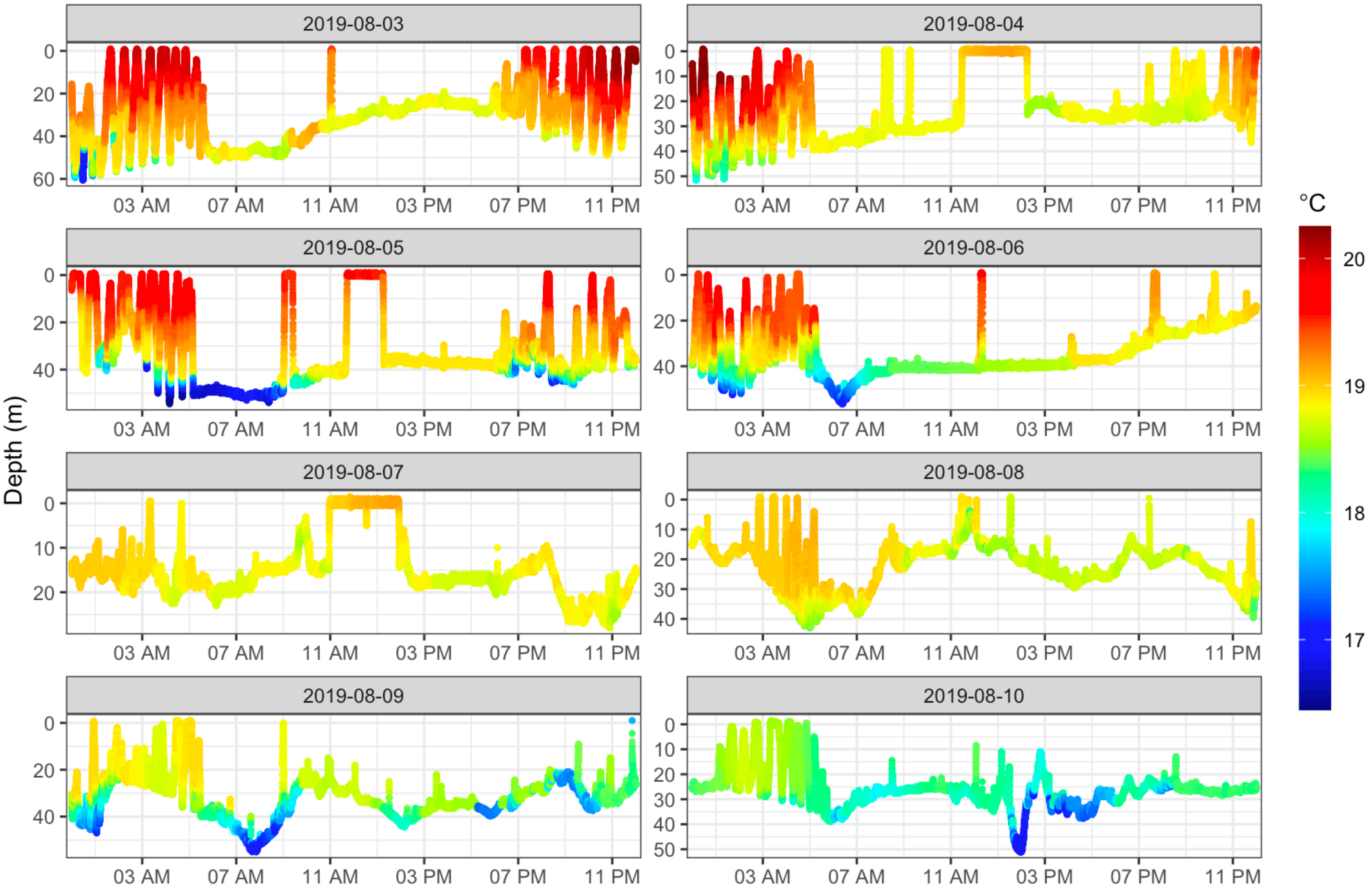
2019-08-03
2019-08-04
2019-08-05
2019-08-06
2019-08-07
2019-08-08
2019-08-09
2019-08-10
Depth (m)
03 AM
07 AM
11 AM
03 PM
07 PM
11 PM
°C
20
19
18
17

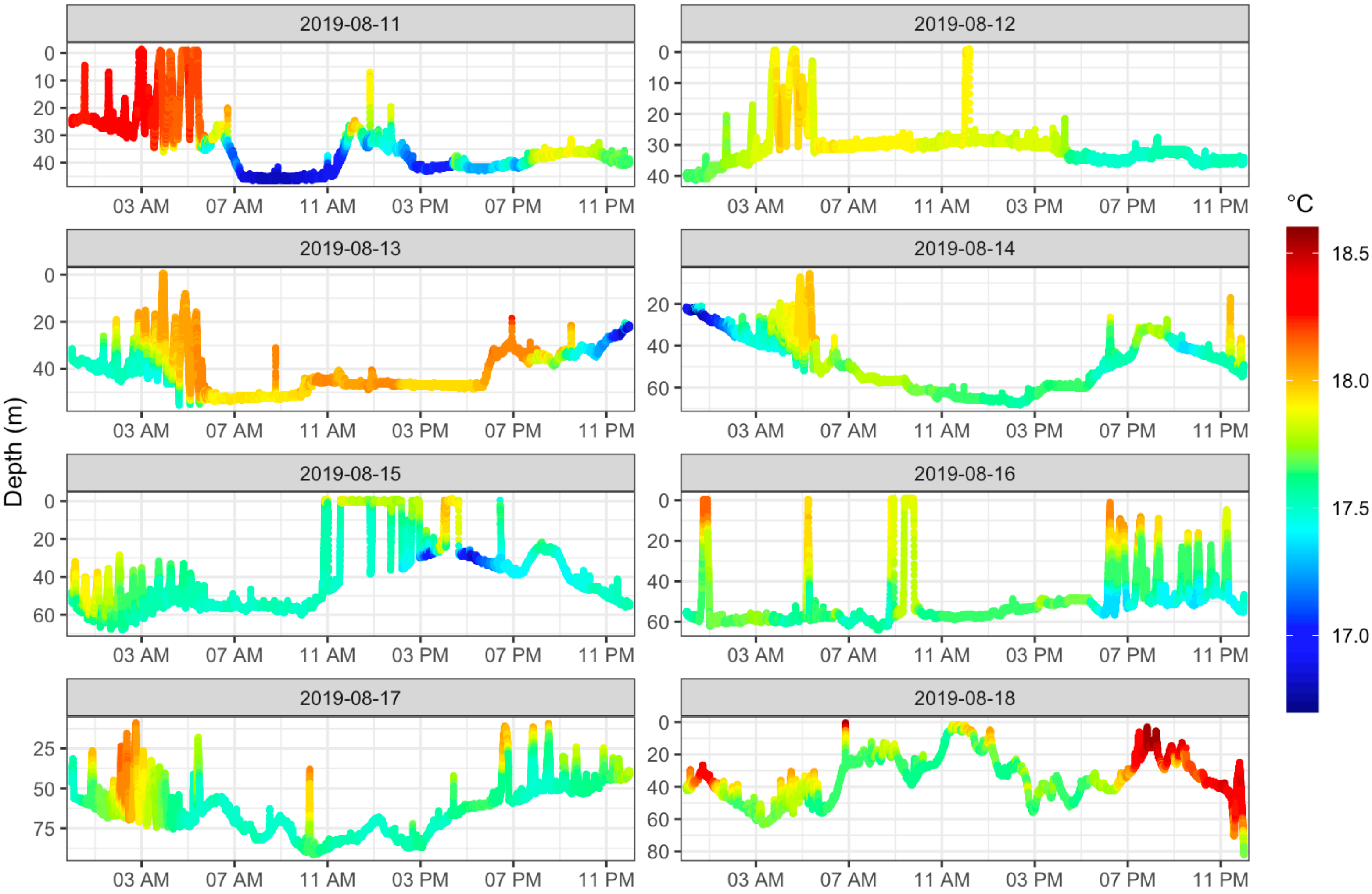

Depth (m)
2019-08-11
2019-08-12
2019-08-13
2019-08-14
2019-08-15
2019-08-16
2019-08-17
2019-08-18
03 AM
07 AM
11 AM
03 PM
07 PM
11 PM
°C
18.5
18.0
17.5
17.0

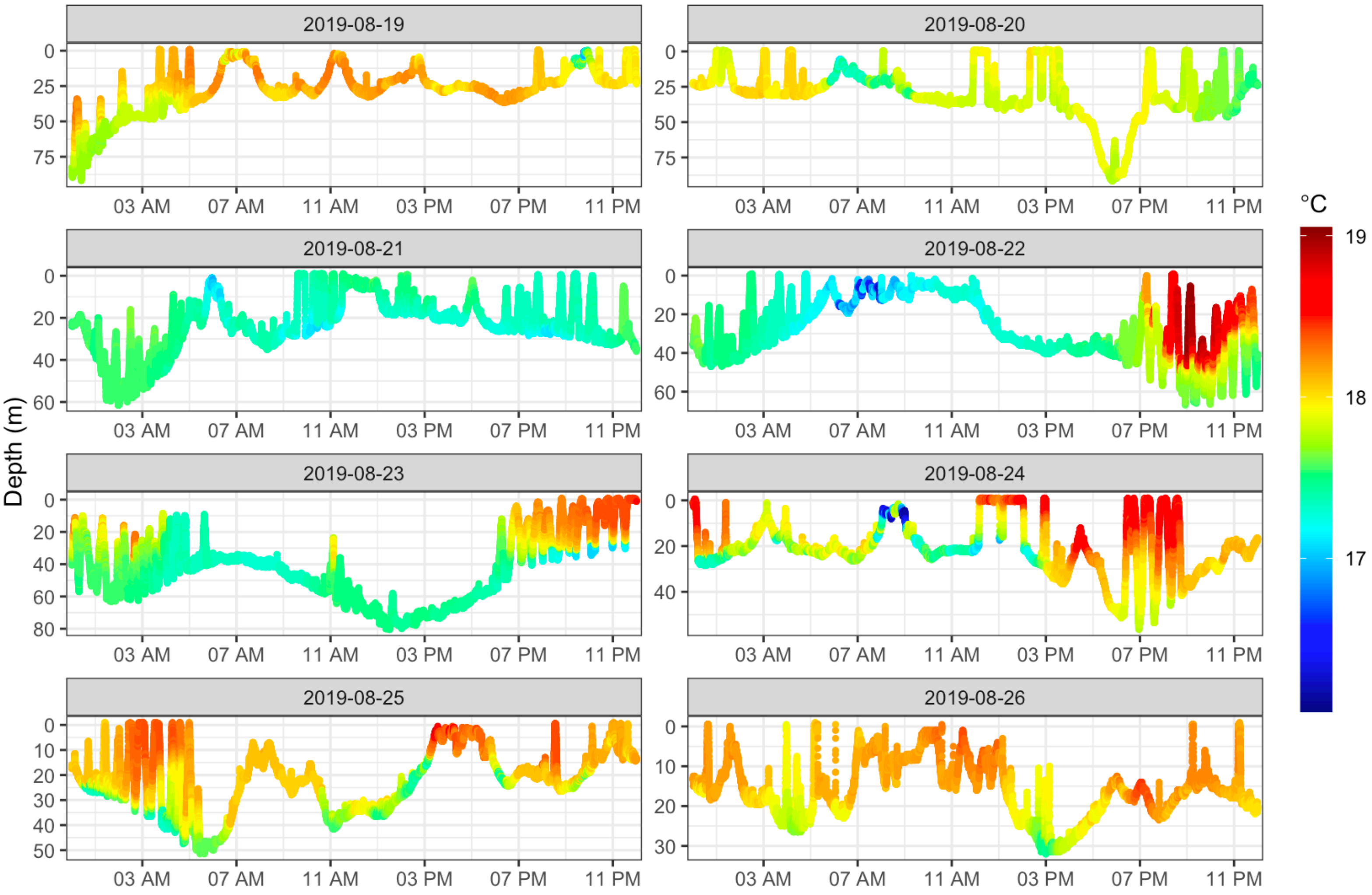
2019-08-19
2019-08-20
2019-08-21
2019-08-22
2019-08-23
2019-08-24
2019-08-25
2019-08-26
Depth (m)
°C
19
18
17
03 AM
07 AM
11 AM
03 PM
07 PM
11 PM

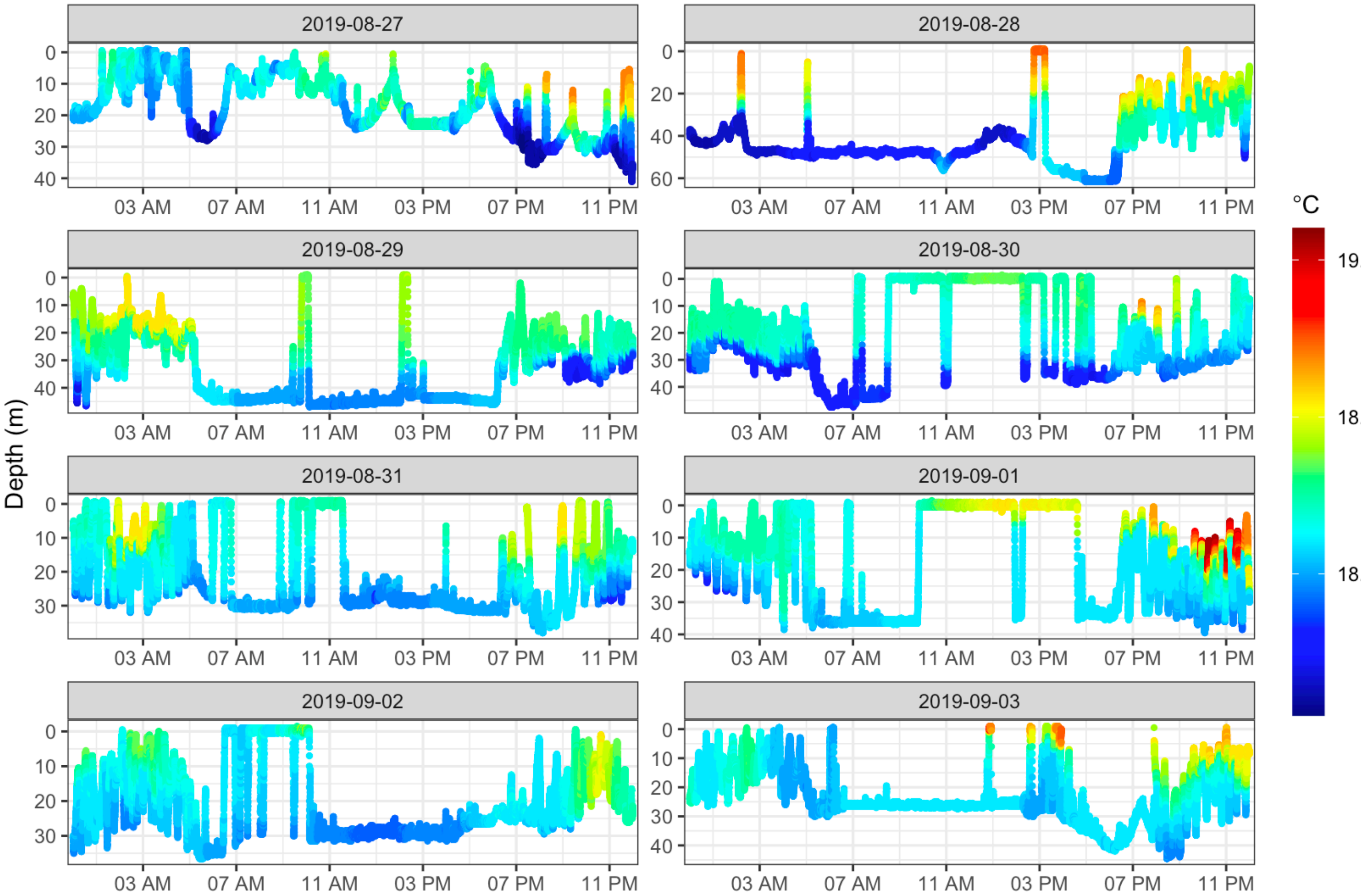
2019-08-27
2019-08-28
2019-08-29
2019-08-30
2019-08-31
2019-09-01
2019-09-02
2019-09-03
Depth (m)
03 AM
07 AM
11 AM
03 PM
07 PM
11 PM
°C
19.0
18.5
18.0

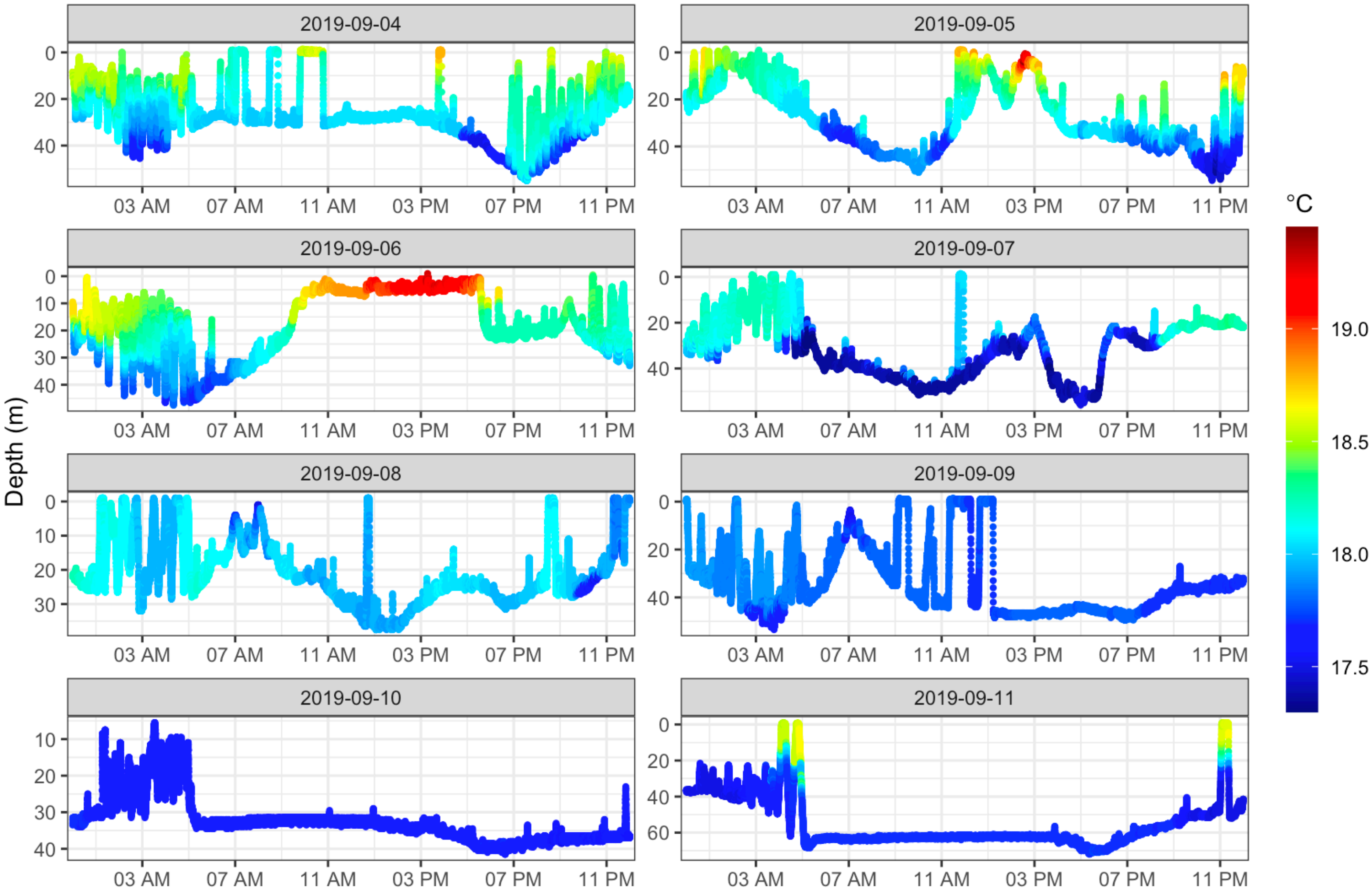

2019-09-04
2019-09-05
2019-09-06
2019-09-07
2019-09-08
2019-09-09
2019-09-10
2019-09-11
Depth (m)
°C
19.0
18.5
18.0
17.5
03 AM
07 AM
11 AM
03 PM
07 PM
11 PM

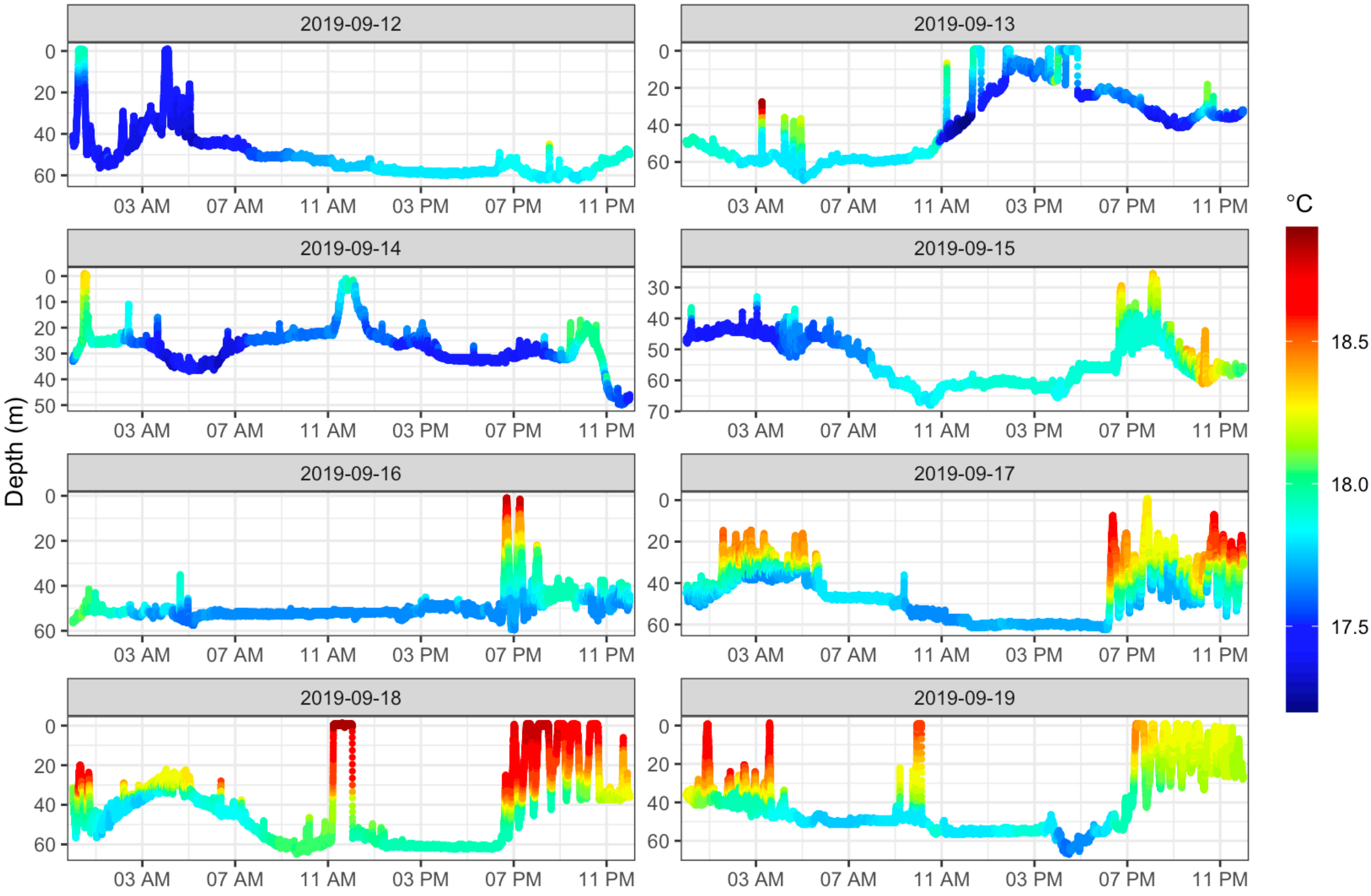
2019-09-12
2019-09-13
2019-09-14
2019-09-15
2019-09-16
2019-09-17
2019-09-18
2019-09-19
Depth (m)
03 AM
07 AM
11 AM
03 PM
07 PM
11 PM
°C
18.5
18.0
17.5

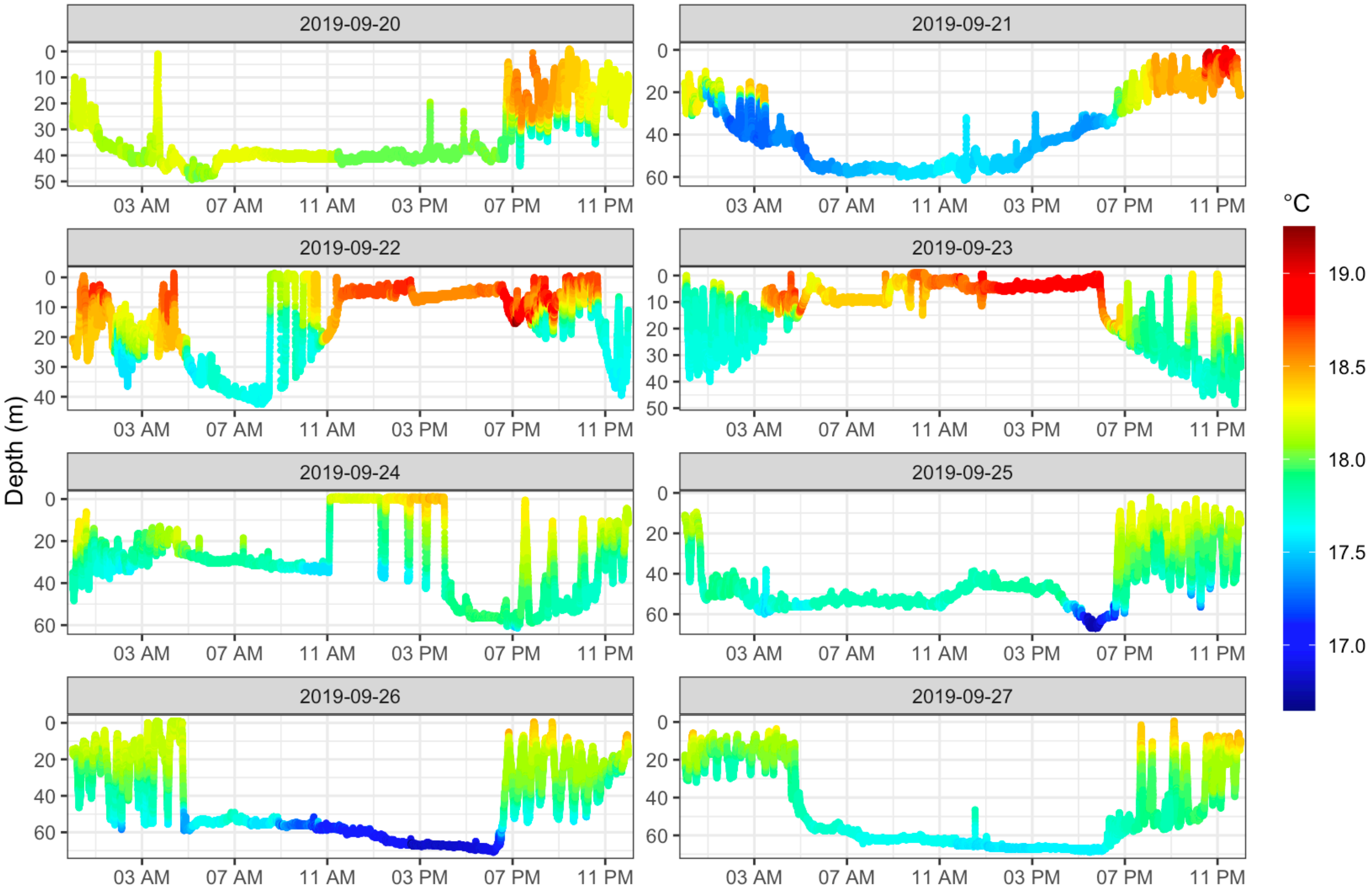

2019-09-20
2019-09-21
2019-09-22
2019-09-23
2019-09-24
2019-09-25
2019-09-26
2019-09-27
Depth (m)
03 AM
07 AM
11 AM
03 PM
07 PM
11 PM
°C
19.0
18.5
18.0
17.5
17.0

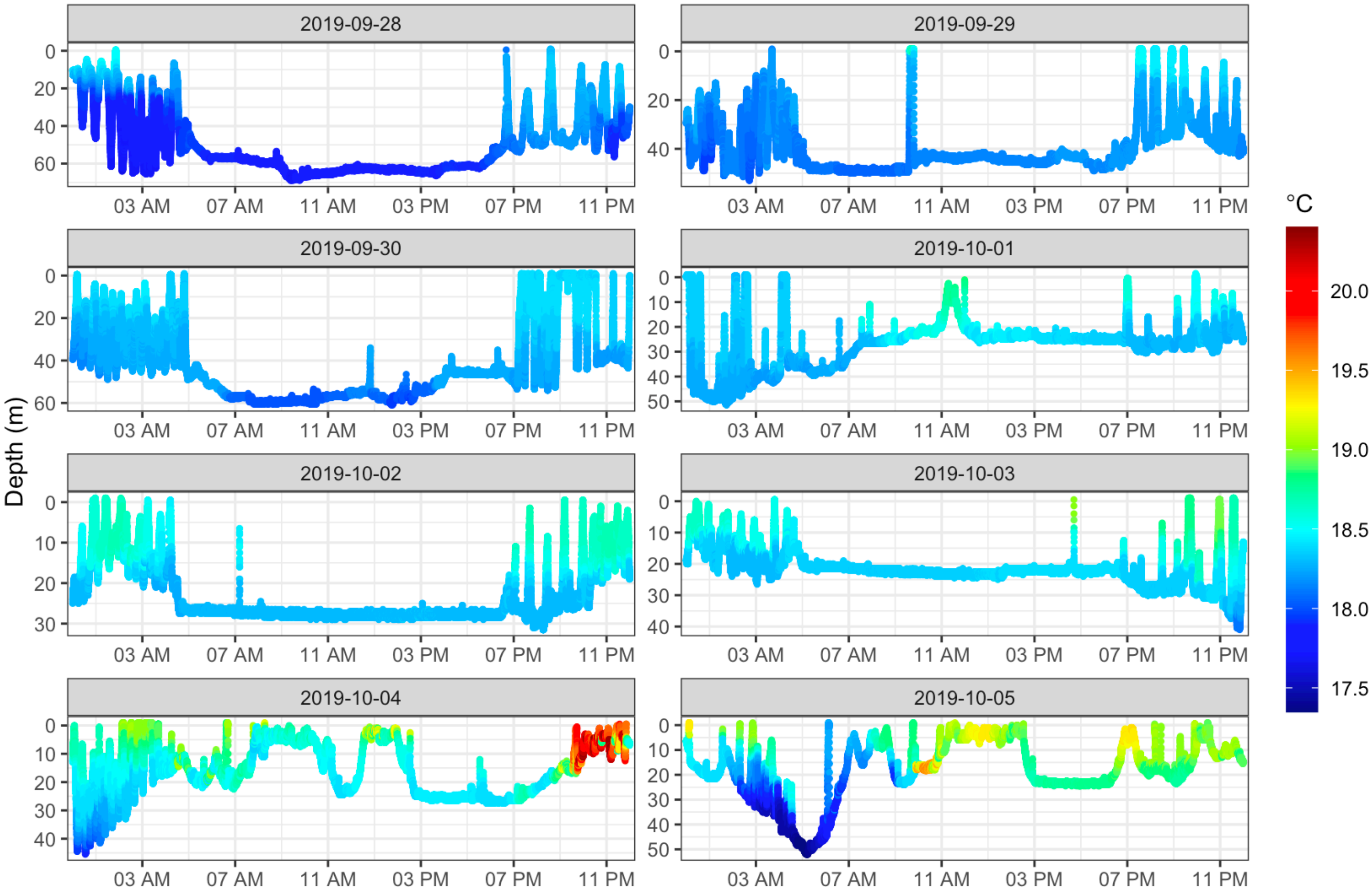
2019-09-28
2019-09-29
2019-09-30
2019-10-01
2019-10-02
2019-10-03
2019-10-04
2019-10-05
Depth (m)
03 AM
07 AM
11 AM
03 PM
07 PM
11 PM
°C
20.0
19.5
19.0
18.5
18.0
17.5

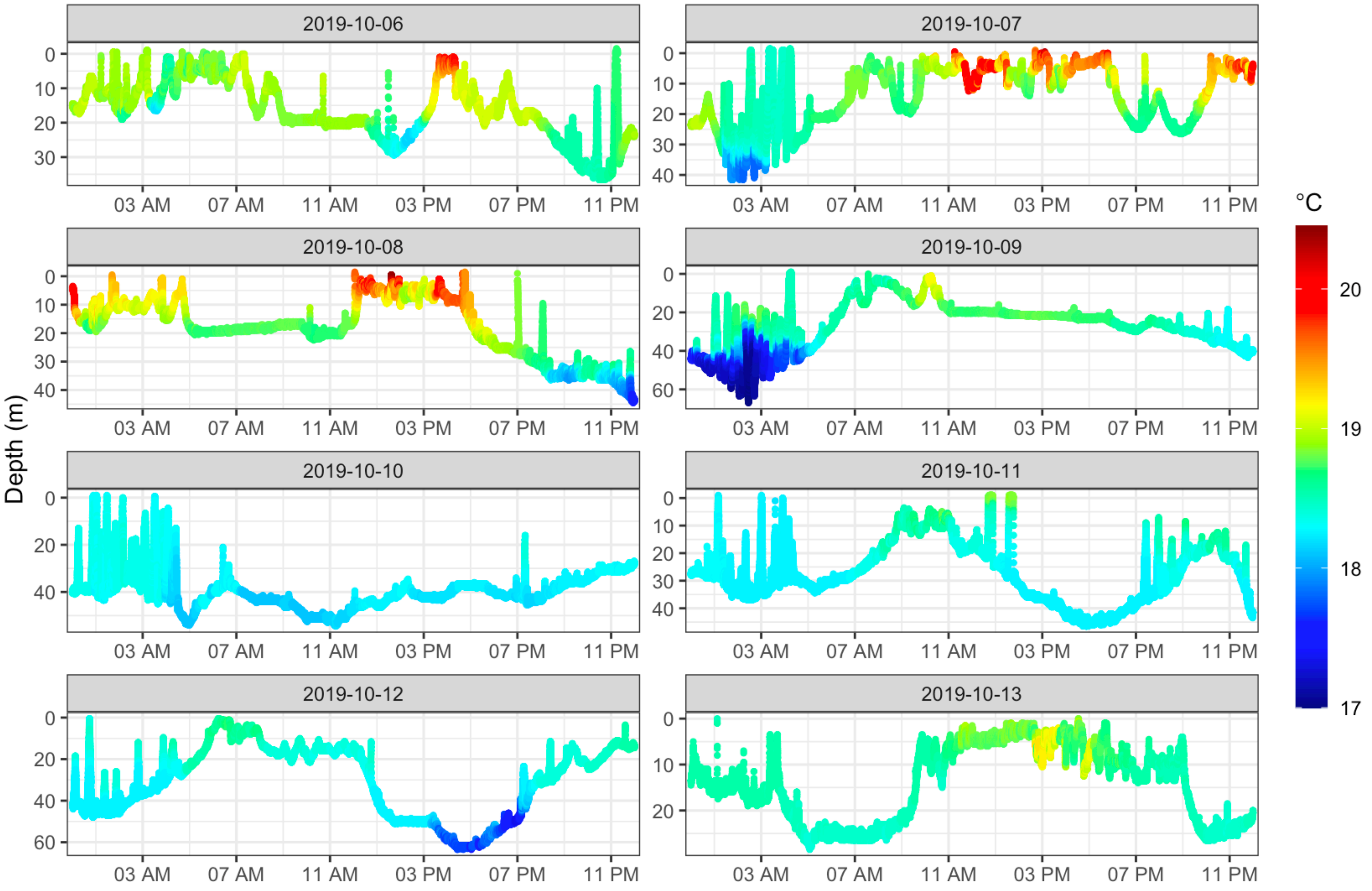

2019-10-06
2019-10-07
2019-10-08
2019-10-09
2019-10-10
2019-10-11
2019-10-12
2019-10-13
Depth (m)
03 AM
07 AM
11 AM
03 PM
07 PM
11 PM
°C
20
19
18
17

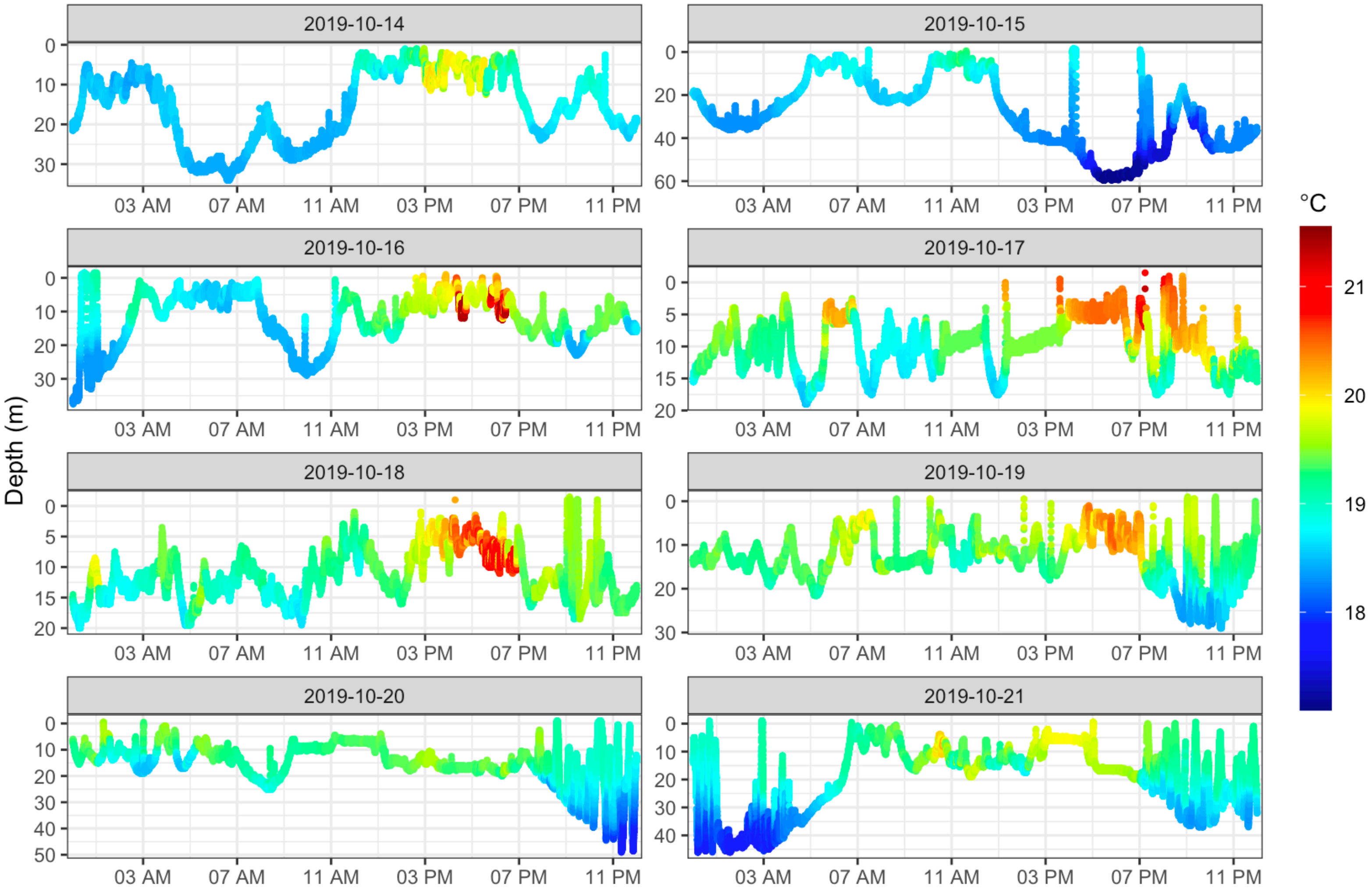

2019-10-14
2019-10-15
2019-10-16
2019-10-17
2019-10-18
2019-10-19
2019-10-20
2019-10-21
Depth (m)
03 AM
07 AM
11 AM
03 PM
07 PM
11 PM
°C
21
20
19
18

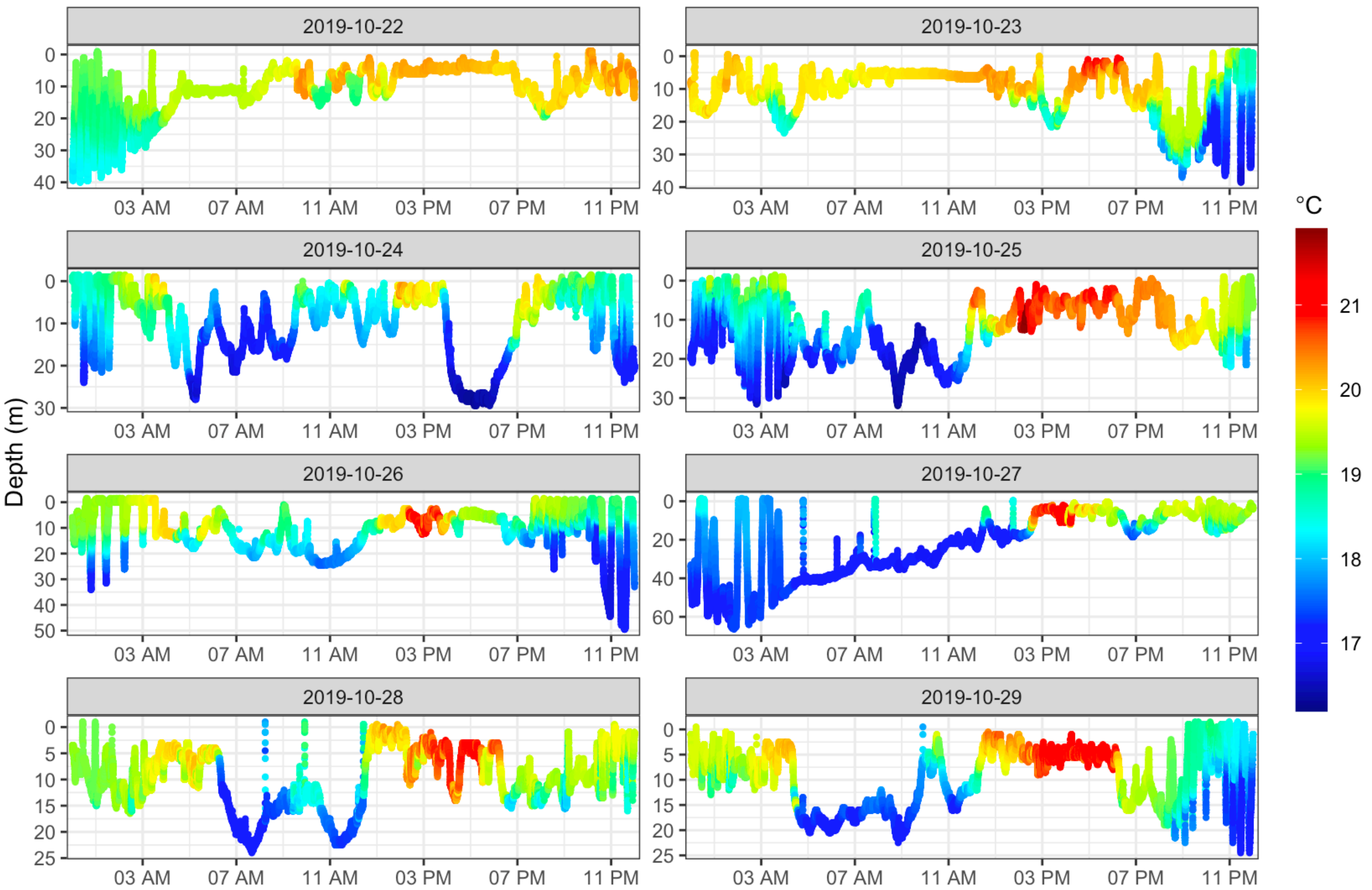

2019-10-22
2019-10-23
2019-10-24
2019-10-25
2019-10-26
2019-10-27
2019-10-28
2019-10-29
03 AM
07 AM
11 AM
03 PM
07 PM
11 PM
Depth (m)
°C
21
20
19
18
17

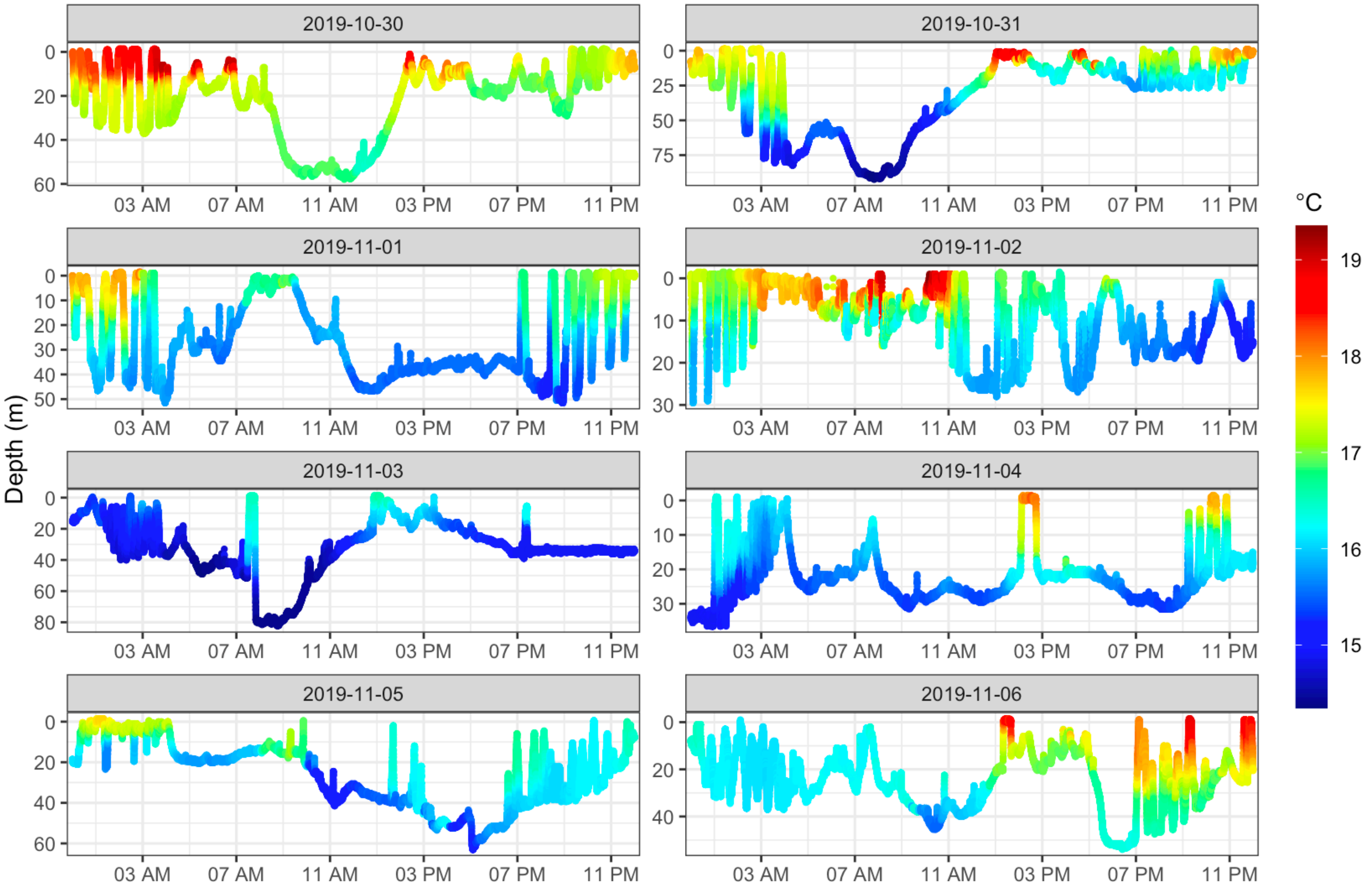
2019-10-30
2019-10-31
2019-11-01
2019-11-02
2019-11-03
2019-11-04
2019-11-05
2019-11-06
Depth (m)
03 AM
07 AM
11 AM
03 PM
07 PM
11 PM
°C
19
18
17
16
15

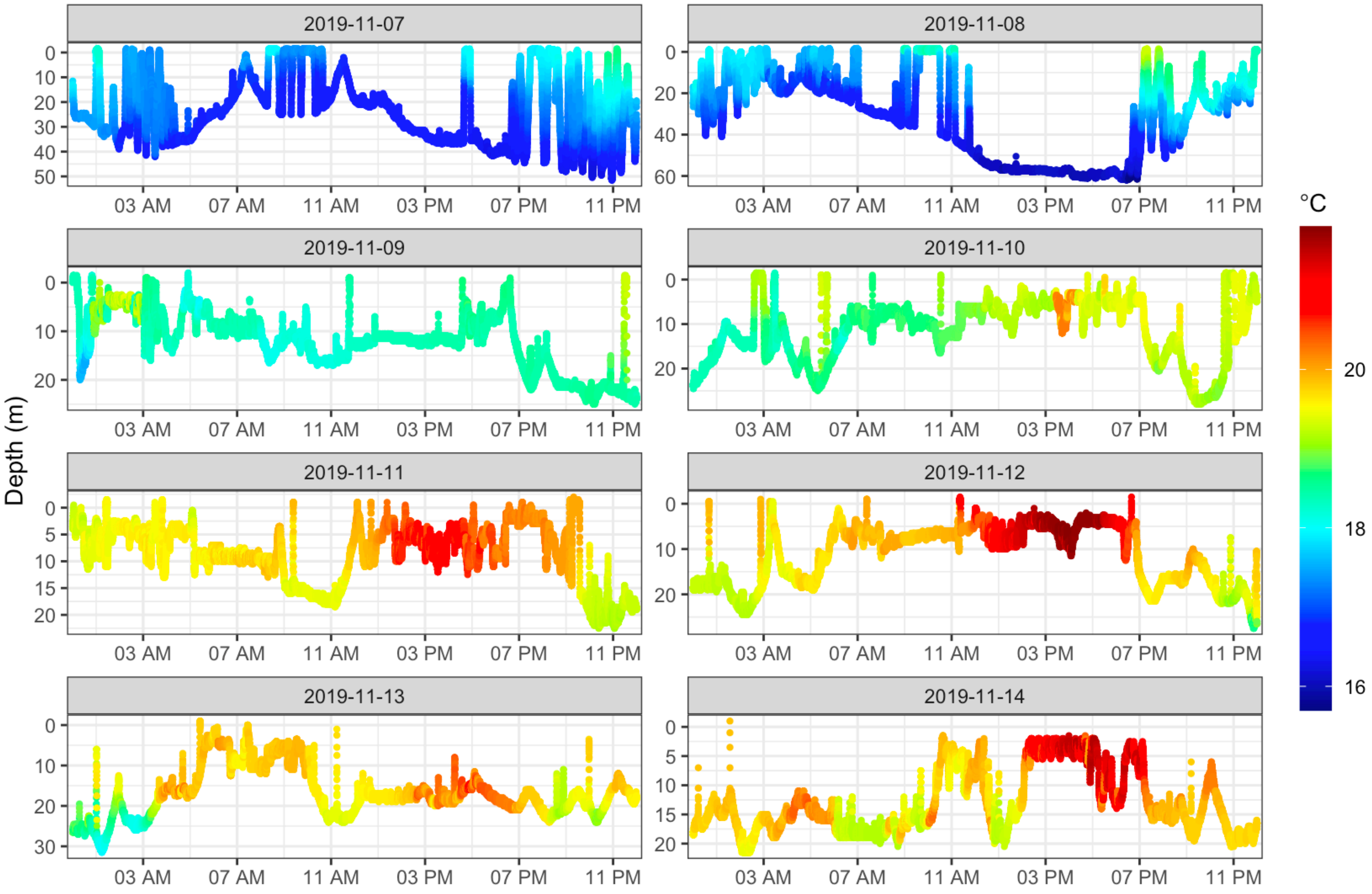

2019-11-07
2019-11-08
2019-11-09
2019-11-10
2019-11-11
2019-11-12
2019-11-13
2019-11-14
Depth (m)
03 AM
07 AM
11 AM
03 PM
07 PM
11 PM
°C
20
18
16

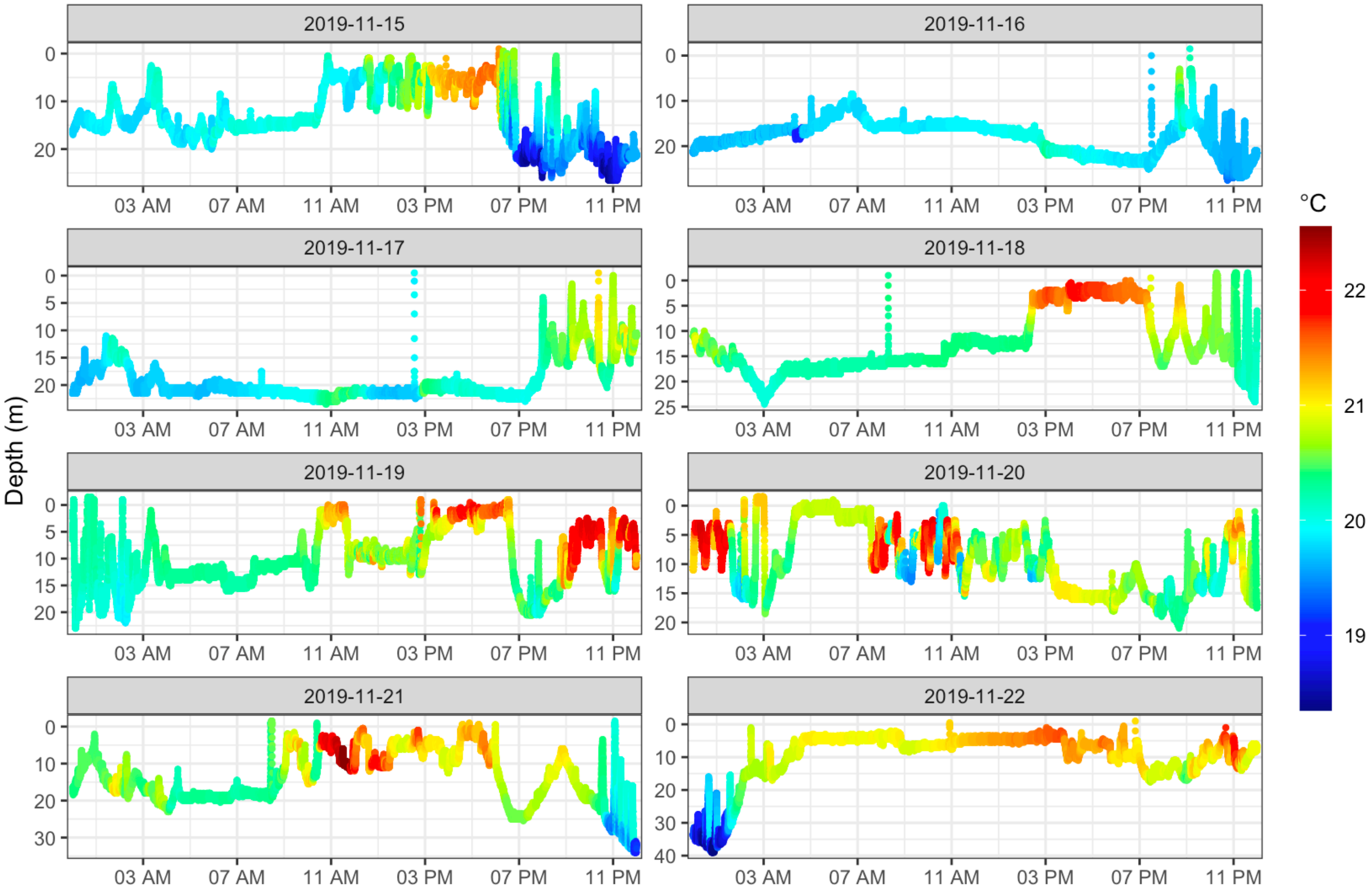

2019-11-15
2019-11-16
2019-11-17
2019-11-18
2019-11-19
2019-11-20
2019-11-21
2019-11-22
Depth (m)
03 AM
07 AM
11 AM
03 PM
07 PM
11 PM
°C
22
21
20
19

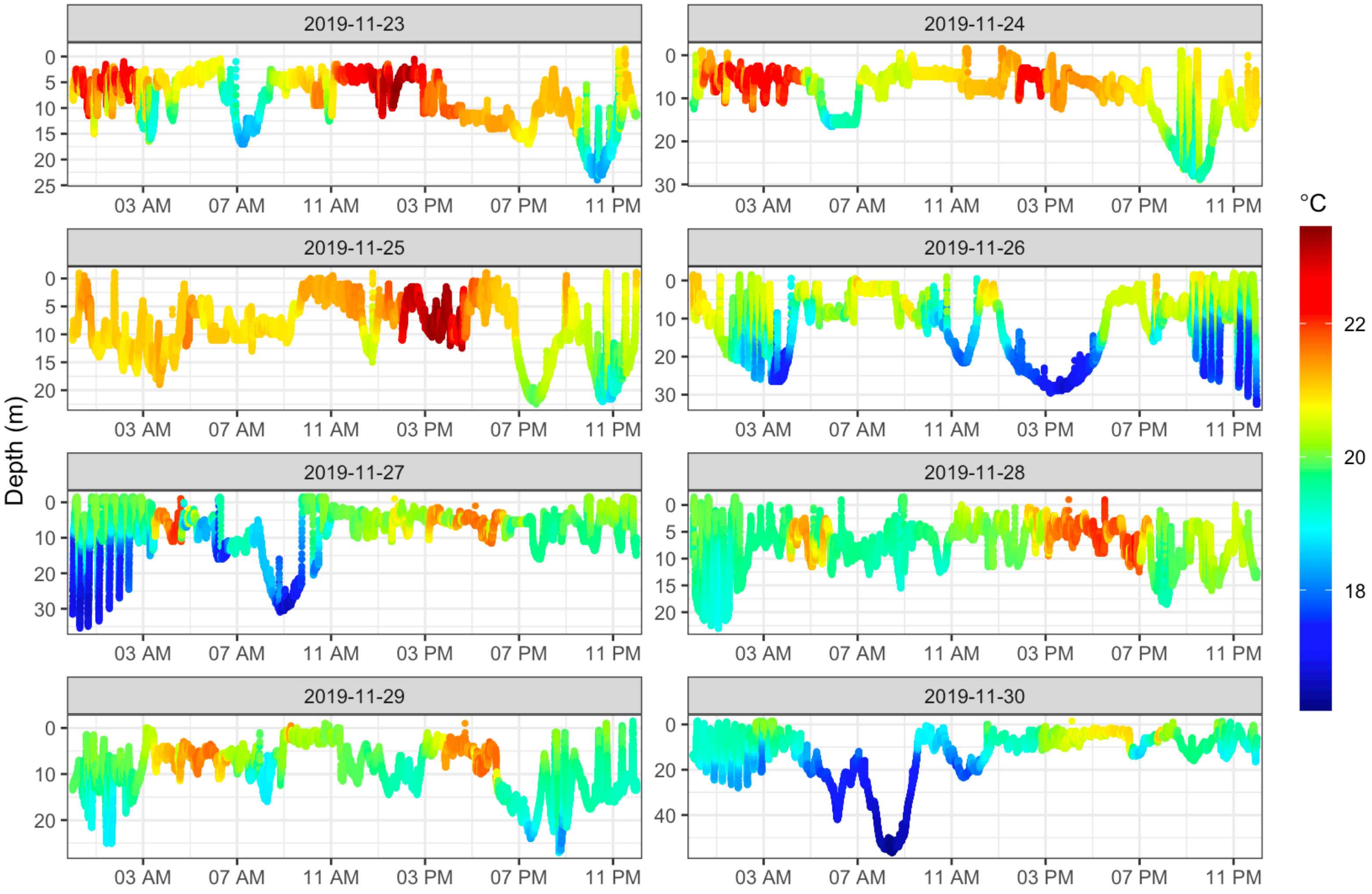
2019-11-23
2019-11-24
2019-11-25
2019-11-26
2019-11-27
2019-11-28
2019-11-29
2019-11-30
Depth (m)
03 AM
07 AM
11 AM
03 PM
07 PM
11 PM
°C
22
20
18

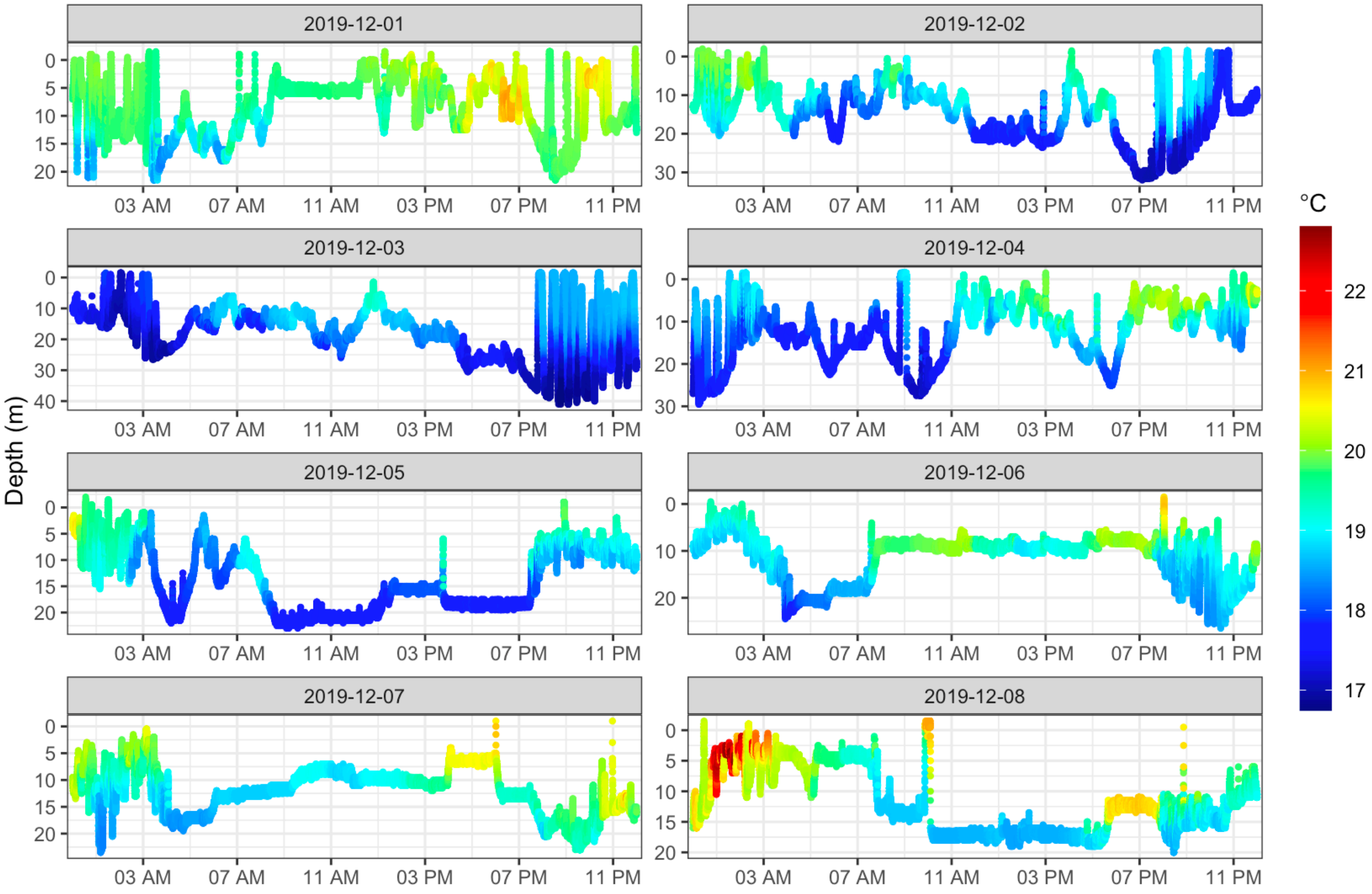
2019-12-01
2019-12-02
2019-12-03
2019-12-04
2019-12-05
2019-12-06
2019-12-07
2019-12-08
Depth (m)
°C
03 AM
07 AM
11 AM
03 PM
07 PM
11 PM

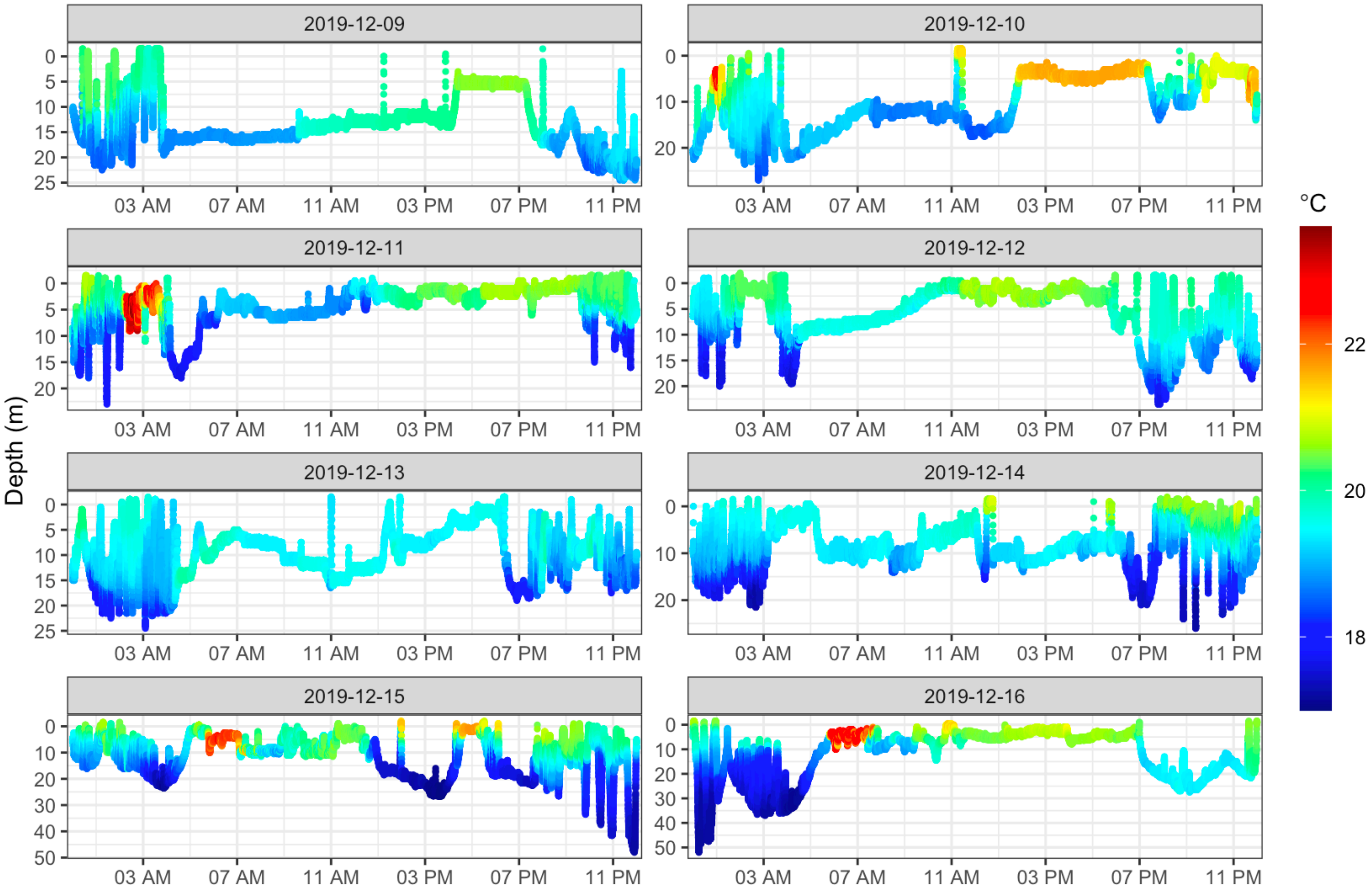

2019-12-09
2019-12-10
2019-12-11
2019-12-12
2019-12-13
2019-12-14
2019-12-15
2019-12-16
Depth (m)
03 AM
07 AM
11 AM
03 PM
07 PM
11 PM
°C
22
20
18

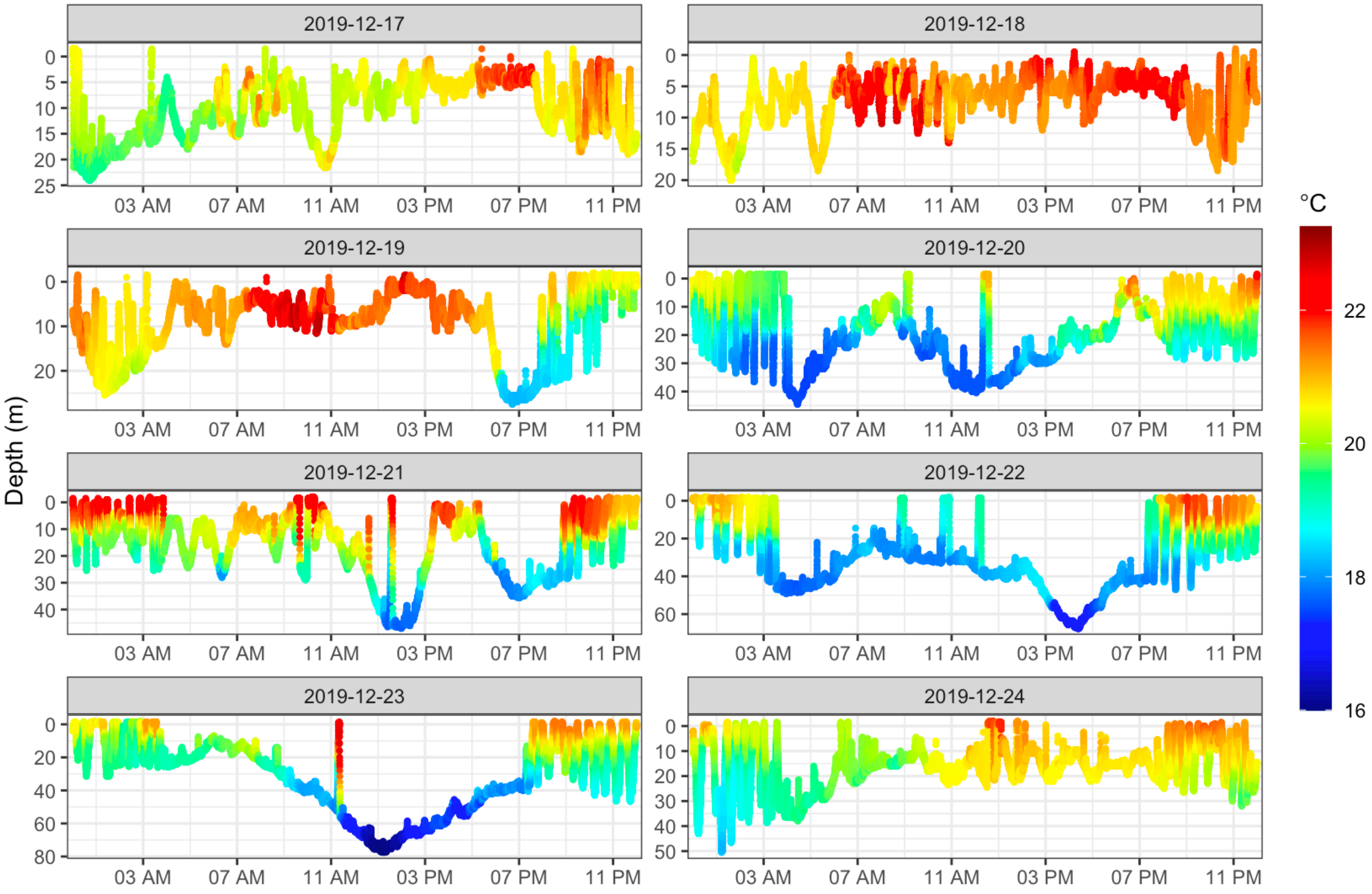

2019-12-17
2019-12-18
2019-12-19
2019-12-20
2019-12-21
2019-12-22
2019-12-23
2019-12-24
Depth (m)
°C
22
20
18
16
03 AM
07 AM
11 AM
03 PM
07 PM
11 PM

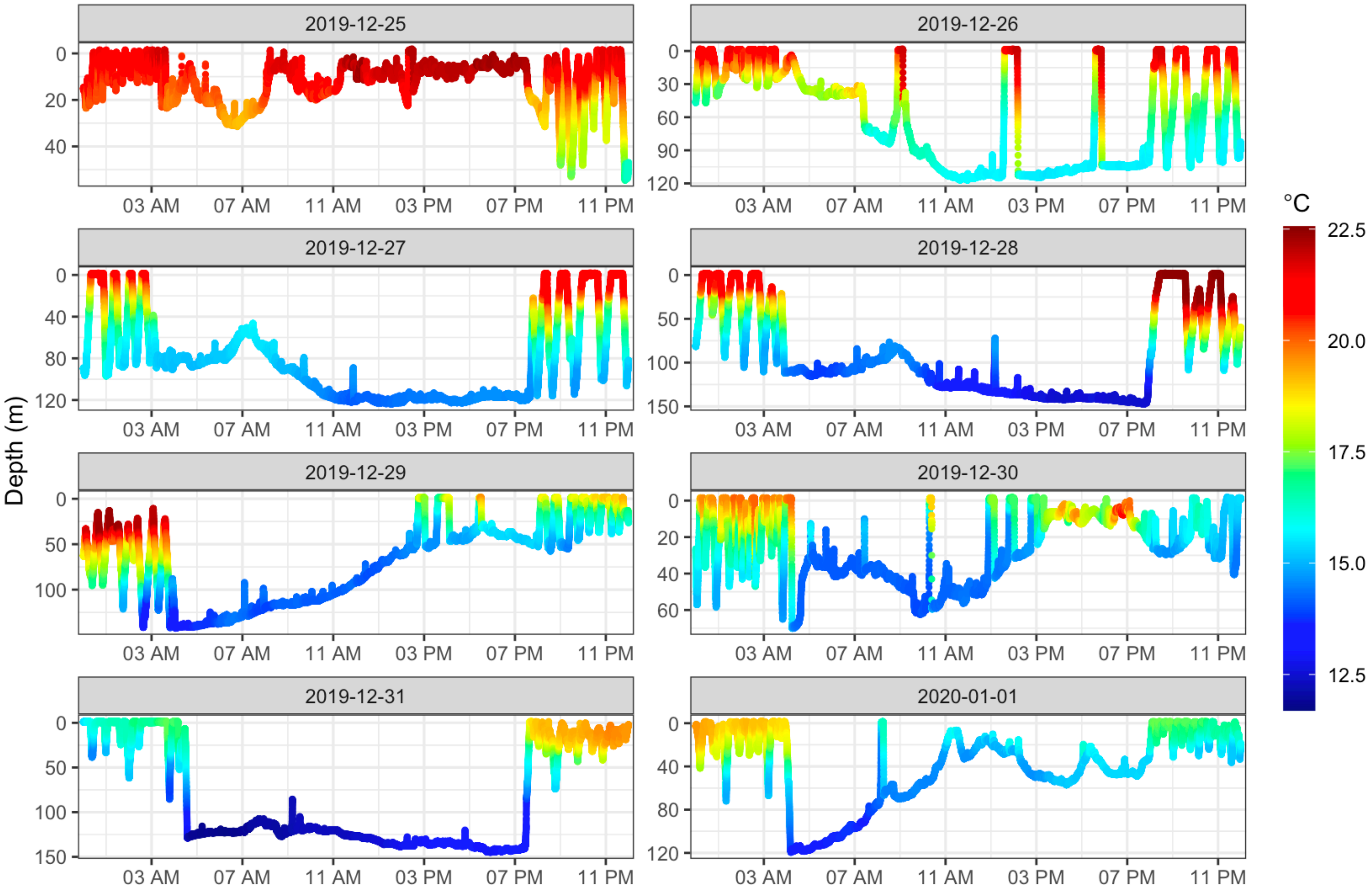
2019-12-25
2019-12-26
2019-12-27
2019-12-28
2019-12-29
2019-12-30
2019-12-31
2020-01-01
Depth (m)
03 AM
07 AM
11 AM
03 PM
07 PM
11 PM
°C
22.5
20.0
17.5
15.0
12.5

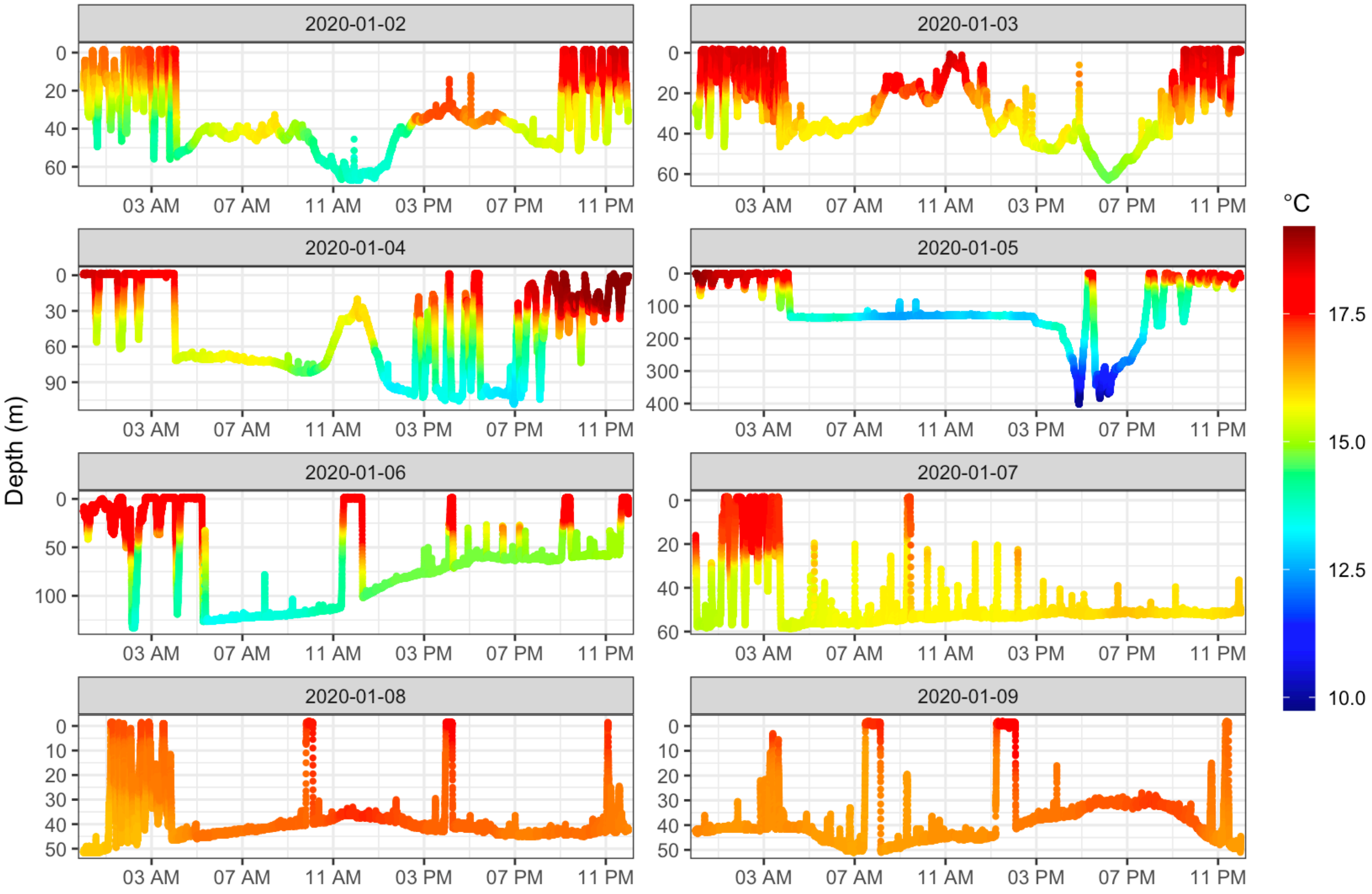

2020-01-02
2020-01-03
2020-01-04
2020-01-05
2020-01-06
2020-01-07
2020-01-08
2020-01-09
Depth (m)
°C
17.5
15.0
12.5
10.0
03 AM
07 AM
11 AM
03 PM
07 PM
11 PM

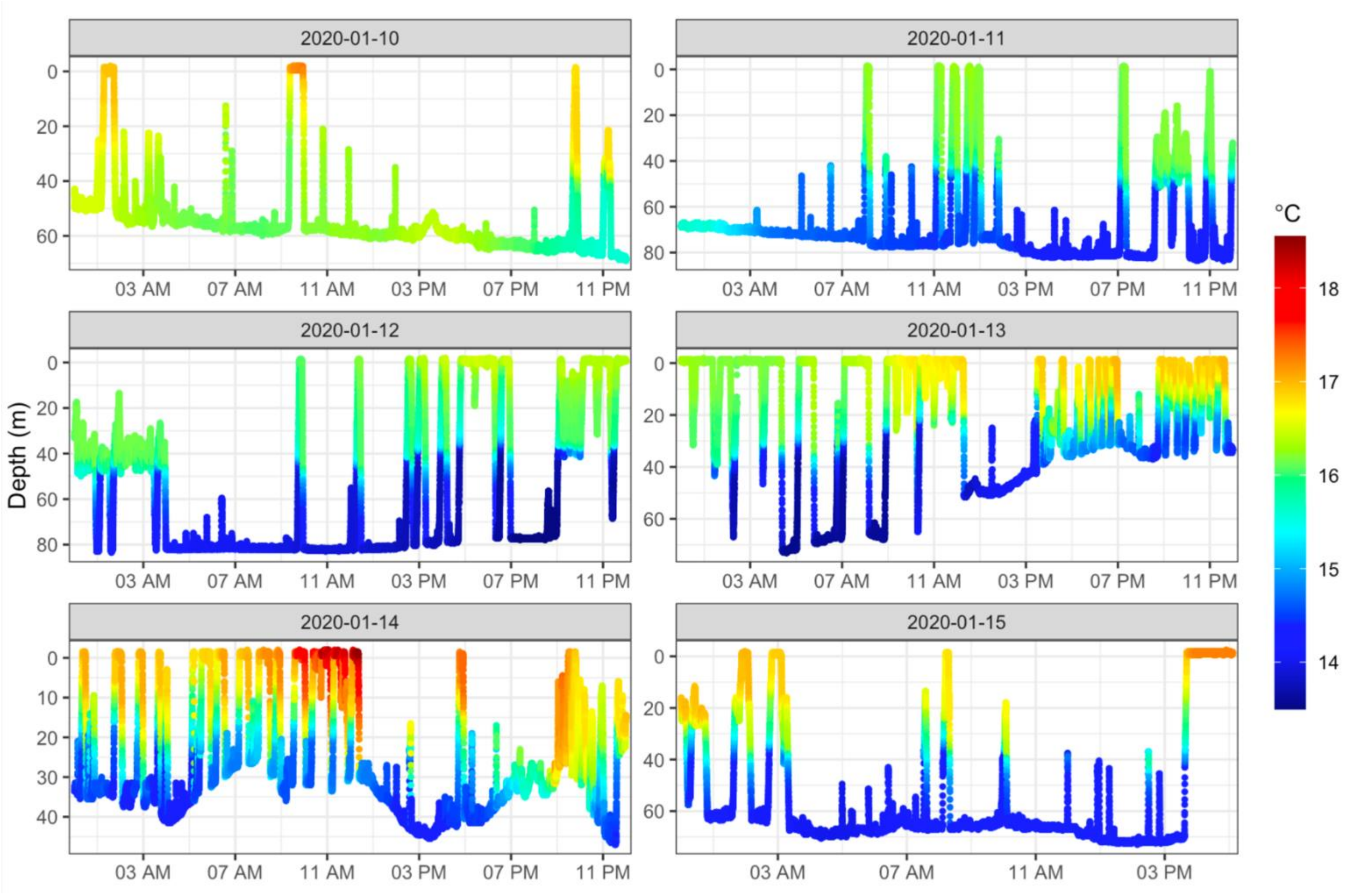

Figure S6. Dive profiles. Archival time-series depth and temperature data of sharks 180 (210 cm FL, female); 356 (184 cm FL, female); 365 (174 cm FL, male) and 366 (193 cm FL, male), respectively. Depth and temperature profiles recorded at 3-s intervals over the entire tracking period for each shark.

LITERATURE CITED

Pante E, Simon-Bouhet B (2013) Marmap: a package for importing, plotting and analyzing bathymetric and topographic data in R. PLoS One 8:e73051.
